# Supplementary material for: A framework for visualizing and describing city image promotion short video data based on microcube model
Source: PLoS One. 2025 Apr 10;20(4):e0317883. doi: 10.1371/journal.pone.0317883 (PMC11984742; doi:10.1371/journal.pone.0317883)
Supplement: Attachment — Submitted filename: A framework for visualizing PONE-D-24-25058_R1_reviewer.pdf [file pone.0317883.s002.pdf]

PLOS ONE

A framework for visualizing and describing city image promotion short video data based on microcube model

--Manuscript Draft--

**Manuscript Number:**	PONE-D-24-25058R1
**Article Type:**	Research Article
**Full Title:**	A framework for visualizing and describing city image promotion short video data based on microcube model
**Short Title:**	A framework for visualizing and describing city image promotion short video data
**Corresponding Author:**	Jing He<br>Institute for Advanced Studies in Humanities and Social Science, Beihang University<br>Hefei, CHINA
**Keywords:**	City Image Promotion; Short Video; Micro Perspective; Imagery Elements
**Abstract:**	Background: The rapid development of media technology and media environment provides rich resources and convenient ways to shape the image of the city. Short video has become an important help to shape the image of the city and build the city brand. How to use short video to shape the image of the city is the key link of urban construction. How: In order to realize comprehensive analysis of short video content, this paper collected a total of 20668 video screenshots for the Douyin platform as data samples, and used Python programming language and Pandas and Matplotlib libraries to process and visualize the data. From the microscopic point of view, a short video data analysis framework of city image promotion based on microcube model is constructed. Objective: To reveal the relationship between video content features and popularity by quantitative and visual methods, and to provide reference for optimizing urban brand promotion strategies. Conclusion: (1) The microcube model shows strong flexibility and applicability in short video content analysis, and can help reveal the complex relationship between short video content characteristics and communication effect. (2) Unexpected events and emotional resonance are key factors in the attractiveness and communication effect of short videos. Reasonable design of scene switching and element interaction significantly enhances the visual impact of short videos.
**Order of Authors:**	Jing He
	Zhuoluo Yang
	Jiayi Zhu
**Response to Reviewers:**	Review Comments to the Author<br><br>Please use the space provided to explain your answers to the questions above. You may also include additional comments for the author, including concerns about dual publication, research ethics, or publication ethics. (Please upload your review as an attachment if it exceeds 20,000 characters)<br>Reviewer #1:<br><br>Authors attempt to work on a very interesting topic regarding showcasing cities for the purpose of popularity and prosperity however the real problem is not clear as to why this work is necessary.<br>Response: Thank the reviewers for their careful reading and valuable comments on this article. We recognize that there is room for improvement in clarifying the context and necessity of the study. In the process of revision, we re-sorted out the problem consciousness and goal of the research, further supplemented the specific description of the research background and the potential application value of this research in the analysis of short video content of urban image promotion.<br><br>It is recommended that authors should remove the sections indicated in abstract but only retain the logical structure. Also, the introduction section looks shallow and the background to this study requires more robust introduction section reflecting the real

Powered by Editorial Manager® and ProduXion Manager® from Aries Systems Corporation

problem with global relevance for proper perspective.
Response: Thanks to the reviewers for their pertinent suggestions on this paper, especially for the guidance on simplifying the content of the abstract and perfecting the background of the introduction. We recognize that the current summary is somewhat redundant in the description of technical details, which may reduce the clarity of the core logical structure. According to the recommendations of the reviewers, we have deleted technical expressions in the revision and highlighted the logical thread in order to more directly demonstrate the research objectives and significance. In addition, as for the problems in the introduction, we are deeply aware that the original text lacks in the breadth and depth of the research background, especially in the context of globalization, the expression of relevance still needs to be strengthened. To this end, we supplement the status quo and challenges of global urban brand communication, and combine the communication characteristics of short video, a new medium, to clarify the practical significance and scope of application of this study. It is hoped that these modifications can better reflect the overall logic and research value of the paper.

It is important to reflect on how such framework could affect tourism either positively or negatively due to the fact that some cities are dream land to many globally and the moment they view such videos it could impact their decisions in both ways hence the framework must consider this effect.
Response: Thanks to the reviewers for their suggestions on this paper, especially the concern that the research framework may influence travel decisions. We recognize that in the context of globalization, the potential positive and negative effects of short video content on the tourism industry deserve further discussion. In the revision, we have added relevant background information in the introduction, clearly pointing out the two-way possibilities of short videos in attracting tourists and affecting the image of the city. At the same time, in the discussion part, the specific analysis of the influence of the framework on tourism decision-making is added, including the possibility of positive and negative effects and countermeasures.

The source of the TikTok video is missing even though authors stated that they were verified. Where were this data stored for the purpose of this research among other data integrity concerns.
Response: Thank the reviewers for their attention to the source and completeness of the data in this paper. These issues are crucial to the credibility of the research, and we have made the following improvements in the revision: In the part of methodology, we detailed the collection and verification process of TikTok video data, including screening criteria, verification mechanism and denoising methods, to ensure the authenticity and representativeness of the data, and added specific measures for data storage and integrity protection.

While commending the analysis of cases and extensive review carried out by authors figures e.g. figure 7 is not clear enough to relate with as much as readership requires great improvement. It is recommended that authors should device an alternative means of representing this fact.
Response: Figure 7 has been reuploaded and improved to clearly label the variable meanings of each column and row, while ensuring good readability in both digital and print media by increasing color contrast and labeling.

For clarity it is important for the authors to discuss results separately from conclusion as it looks mixed up and vague thereby making it difficult to see in clear terms what achievement is recorded by this work.
Response: Thanks to the reviewers for their meticulous feedback on the structure of this paper. We recognize that mixing "results" with "conclusions" can affect readers' understanding of research findings and summaries. To this end, during the revision process, we reorganized the paper to include a separate "results" section to elaborate on specific findings based on the data analysis; At the same time, the "conclusion" part is adjusted to summarize the significance, practical application, limitations and future direction of the research.

Grammatical flow should be checked by authors for consistency.
Response: Thanks to the reviewers for their valuable comments on the grammatical coherence of this paper. We are deeply aware of the importance of grammatical accuracy and fluency in the presentation of academic articles. Based on your

Powered by Editorial Manager® and ProduXion Manager® from Aries Systems Corporation

suggestions, we have made checked by authors for consistency to the full text.
Reviewer #2:
A framework for visualizing and describing city image promotion short video data based on microcube model.
Reviewed by: Riyaz Abro.
Observations (If appropriate & Applicable).
• Abstract:
o Which software was used to analyse the data, include timeframe and time limit of the data that from what time and period the data was collected.
Response: Thanks to the reviewers for their valuable suggestions on the summary. We recognize that clarity about the software tools used for data analysis and the time frame of the data helps improve transparency and reproducibility. In response to your suggestions, we have supplemented the data sources and research methods in the Summary and Methods section. Regarding the software used for data analysis, we utilized Python with pandas for data manipulation, scikit-learn for statistical analysis, and matplotlib for visualization. Additionally, deep learning techniques based on PyTorch were employed. Detailed descriptions related to the data are provided in Chapter 4 and Table 1.

• Introduction:
o Already given variables in keywords are different than the defined ones
Response: "City image publicity" has been changed to "city image promotion", and "microscopic content analysis" have been modified to "Micro Perspective".

• Literature Review:
o Given variables must be defined (if possible).
Response: We have revised the manuscript to include precise definitions of the key variables, such as "unexpected events," "emotional resonance," "scene transition," "elemental amplification," "element interaction," and "screen style." These definitions have been integrated into the literature review section to enhance clarity and provide a better understanding of their role in our research framework.

• Precise Conclusion:
o Limitation must be defined, hypotheses must be generated separately, however, paper is very much innovative and be published with minor correction as suggested and if deemed appropriate.
Response: Thank you very much for your insightful comments regarding the need for a precise conclusion. Based on your suggestions, we have further refined the "Conclusion and Discussion" section to clearly define the limitations of our study and have also generated the hypotheses separately. By doing so, we aim to provide a more structured and clear presentation of our research contributions.
Reviewer #3:
1.Explain the immersion methos or techniques based on micro cube model
Response: The immersion method based on the microcube model aims to analyze and enhance the viewer's immersive experience by breaking down short video content into multiple small content cube units. This model refines the video content by segmenting, assigning, and visualizing key elements within the short video, thereby exploring the impact of these elements on audience emotions and engagement.

2. Also explain how all the parameters are interrelated with each other
Response: The parameters identified in this study—unexpected events, emotional resonance, scene transition, element amplification, element interaction, and screen style—are deeply interrelated, working together to enhance the effectiveness of short videos in promoting city images.
A video that starts with an unexpected event can use emotional resonance to keep viewers engaged, turning their initial curiosity into a deeper emotional connection. Effective use of scene transitions combined with amplified elements can enhance the narrative clarity and visual impact, making the content more memorable. A well-defined screen style enhances element interactions by creating a cohesive visual experience that resonates with viewers, making the city image more distinctive. Each variable influences the others. For example, an unexpected event can be more impactful if accompanied by an emotionally resonant scene, amplified elements, and smooth transitions. Together, these parameters create a holistic effect, making the short video more engaging, memorable, and effective in conveying a city’s image. The absence of

Powered by Editorial Manager® and ProduXion Manager® from Aries Systems Corporation

any one variable can reduce the overall effectiveness, as each plays a crucial role in maintaining viewer interest and enhancing the video's communicative power.

3.One absence of parameter will affect the performance of the short videos
Response: Yes, the absence of any parameter will impact the overall performance of the short video. Including unexpected events in short videos can spark curiosity and quickly capture the audience's interest. Emotional resonance is a key factor in maintaining audience engagement, as emotion-driven content is critical for successful video dissemination. Proper scene transitions help maintain the video's pacing and narrative coherence, allowing viewers to immerse themselves more easily. Element amplification aims to capture viewers' attention through visual impact. Element interaction enhances the video's fun and dynamic presentation, making it more engaging and livelier. A unique screen style helps a video stand out among numerous others, creating a distinct memory point.
Reviewer #4:
This paper uses the Microcube model framework to calculate the unit video content and analyze the packaging time in each microcube, which provides a novel perspective for city image promotion and has certain research value, but there are still the following shortcomings, which I hope to improve:
1. Literature review part of the literature, mainly focused on China literature research, the lack of a broader field and country, the review of relevant research results. In view of the globalization background of short video and urban communication and promotion, the research results of international scholars in the fields of short video content and communication mode should be introduced appropriately. This will help to enhance the global perspective and academic depth of this study, and more comprehensively demonstrate the applicability and innovation of the study on a global scale.
Response: Thanks to the reviewers for their suggestions on the improvement of relevant work parts of this paper. We recognize that the addition of new academic research to each research topic not only improves the depth of the literature review, but also enhances the academic and theoretical support of the paper. According to your suggestions, we have added references from relevant global scholars' research results in six dimensions, namely, accident, emotional resonance, scene switching, element amplification, element interaction, and picture style.

2. Short videos are divided into six categories, but no specific classification basis is provided. The current classification may not fully cover the diversity of short videos, especially when the differences in content and communication targets increase. It is suggested to provide clearer classification standards and consider how to incorporate other types of video content into the classification system to enhance the scientificity and applicability of classification.
Response: We have revised the manuscript and provided detailed definitions of the six imagery elements. These elements are not intended for classifying short videos but were selected due to their significant impact on short video content. Therefore, they serve as the core research dimensions in the analysis framework for city image promotion short videos. These elements were derived from a comprehensive analysis of existing literature and empirical data, allowing us to better reveal the influence of short video content characteristics on city image promotion.

3. The correspondence between color and concept needs to be explained: the study used color to distinguish different elements when discussing short video content, but did not explain in detail how the color corresponds to specific elements.
Response: In the manuscript, we described how specific elements are represented using colors. For example, experimentally, we designed an emotion grid chart with nine emotions: moved, praised, funny, happy, neutral, angry, sad, shocked, and helpless. We used #EE6363, #EEDFCC, #FFA54F, #FFEC8B, #FFFFF0, #00F5FF, #63B8FF, #00FF7F, and #CD69C9 as RGB color controls, respectively, as shown in Figure 2(a). We designed an imagery elements grid with six elements: technology elements, food elements, natural landscape elements, urban landscape elements, people elements, animal elements. We used #00FF7F, #FFEC8B, #EE6363, #00F5FF, #EE82EE, #FFA54F as RGB color controls, as shown in Figure 4(a). And a grid diagram of the main colors of the screen style was designed, and #0000FF, #BEBEBE, #FFFACD, #6495ED, and #FFC0CB were used as RGB color controls for dark, gray, light, medium, and bright tones, respectively, as shown in Figure 6(a).

	4. There is a lack of transparency in the process of constructing the micro-cube model. The micro-cube model is one of the core methods in this study, but at present, the description of the model construction process is abstract, and the existing algorithm (data slicing? Or is it an optimization algorithm for slicing? Or OLAP and multidimensional data modeling?) or self-construction algorithm, the paper does not explain in detail, and the listed algorithms lack detailed explanation. In order to improve the transparency and reproducibility of the model, I hope the author can explain it in detail and provide specific procedures, formulas and data support for the model construction. This will facilitate colleagues to better understand and verify the operability and effectiveness of the model.<br>Response: Thank you for the your suggestion. The model construction process has been addressed in the manuscript. Specifically, we adopt a micro perspective to refine the video content by establishing a content microcube model that utilizes data slicing techniques to segment the complete short video into multiple fragmented units based on time intervals. Each microcube encapsulates the content per unit of time, as illustrated in Figure 1(a).<br><br>5. Scientific methods in the process of short video classification: At present, short video classification methods lack systematic scientific basis and rely on subjective judgment, which may affect the accuracy of classification and the rigor of research results. It is suggested that a more systematic classification method should be introduced in the classification process.<br>Response: Thank you to the reviewer for your valuable suggestions on this paper. To improve efficiency, we used deep learning techniques for short video classification. However, the focus of this study is on the application of the microcube model, rather than exploring how to use deep learning techniques for video classification. Therefore, the classification process is not elaborated in the manuscript, and only the classification results are presented. We hope the reviewer understands the primary focus and direction of our research. Once again, thank you for your suggestions.
Additional Information:	
**Question**	**Response**
**Financial Disclosure**<br><br>Enter a financial disclosure statement that describes the sources of funding for the work included in this submission. Review the submission guidelines for detailed requirements. View published research articles from *PLOS ONE* for specific examples.<br><br>This statement is required for submission and **will appear in the published article** if the submission is accepted. Please make sure it is accurate.	Yes

**Funded studies**<br>Enter a statement with the following details:<br>• Initials of the authors who received each award<br>• Grant numbers awarded to each author<br>• The full name of each funder<br>• URL of each funder website<br>• Did the sponsors or funders play any role in the study design, data collection and analysis, decision to publish, or preparation of the manuscript?<br><br>Did you receive funding for this work?	
Please add funding details.<br>as follow-up to "**Financial Disclosure**<br><br>Enter a financial disclosure statement that describes the sources of funding for the work included in this submission. Review the submission guidelines for detailed requirements. View published research articles from *PLOS ONE* for specific examples.<br><br>This statement is required for submission and **will appear in the published article** if the submission is accepted. Please make sure it is accurate.<br><br>**Funded studies**<br>Enter a statement with the following details:<br>• Initials of the authors who received each award<br>• Grant numbers awarded to each author<br>• The full name of each funder<br>• URL of each funder website<br>• Did the sponsors or funders play any role in the study design, data collection and analysis, decision to publish, or preparation of the manuscript?<br><br>Did you receive funding for this work?"	This study was supported by Research Fund of Guangxi Key Lab of Multi-source Information Mining & Security (No.MIMS22-11), Key Laboratory of Spatial Data Mining & Information Sharing of Ministry of Education, Fuzhou University (No.2023LSDMIS02).
Please select the country of your main research funder (please select carefully as in some cases this is used in fee calculation).	CHINA - CN

as follow-up to "**Financial Disclosure**<br><br>Enter a financial disclosure statement that describes the sources of funding for the work included in this submission. Review the submission guidelines for detailed requirements. View published research articles from *PLOS ONE* for specific examples.<br><br>This statement is required for submission and **will appear in the published article** if the submission is accepted. Please make sure it is accurate.<br><br>**Funded studies**<br>Enter a statement with the following details:<br>• Initials of the authors who received each award<br>• Grant numbers awarded to each author<br>• The full name of each funder<br>• URL of each funder website<br>• Did the sponsors or funders play any role in the study design, data collection and analysis, decision to publish, or preparation of the manuscript?<br><br>Did you receive funding for this work?"	
**Competing Interests**<br><br>Use the instructions below to enter a competing interest statement for this submission. On behalf of all authors, disclose any competing interests that could be perceived to bias this work—acknowledging all financial support and any other relevant financial or non-financial competing interests.<br><br>This statement is required for submission and **will appear in the published article** if the submission is accepted. Please make sure it is accurate and that any funding sources listed in your Funding Information later in the submission form are also declared in your Financial Disclosure statement.	The authors have declared that no competing interests exist.

View published research articles from [*PLOS ONE*] for specific examples.<br><br>**NO authors have competing interests**<br><br>Enter: *The authors have declared that no competing interests exist.*<br><br>**Authors with competing interests**<br><br>Enter competing interest details beginning with this statement:<br><br>*I have read the journal's policy and the authors of this manuscript have the following competing interests: [insert competing interests here]*<br><br>* typeset	
**Ethics Statement**<br><br>Enter an ethics statement for this submission. This statement is required if the study involved:<br><br>• Human participants<br>• Human specimens or tissue<br>• Vertebrate animals or cephalopods<br>• Vertebrate embryos or tissues<br>• Field research<br><br>Write "N/A" if the submission does not require an ethics statement.<br><br>General guidance is provided below. Consult the submission guidelines for detailed instructions. **Make sure that all information entered here is included in the Methods section of the manuscript.**	N/A

Powered by Editorial Manager® and ProduXion Manager® from Aries Systems Corporation

**Format for specific study types**<br><br>**Human Subject Research (involving human participants and/or tissue)**<br>• Give the name of the institutional review board or ethics committee that approved the study<br>• Include the approval number and/or a statement indicating approval of this research<br>• Indicate the form of consent obtained (written/oral) or the reason that consent was not obtained (e.g. the data were analyzed anonymously)<br><br>**Animal Research (involving vertebrate animals, embryos or tissues)**<br>• Provide the name of the Institutional Animal Care and Use Committee (IACUC) or other relevant ethics board that reviewed the study protocol, and indicate whether they approved this research or granted a formal waiver of ethical approval<br>• Include an approval number if one was obtained<br>• If the study involved *non-human primates*, add *additional details* about animal welfare and steps taken to ameliorate suffering<br>• If anesthesia, euthanasia, or any kind of animal sacrifice is part of the study, include briefly which substances and/or methods were applied<br><br>**Field Research**<br><br>Include the following details if this study involves the collection of plant, animal, or other materials from a natural setting:<br>• Field permit number<br>• Name of the institution or relevant body that granted permission	
**Data Availability**<br><br>Authors are required to make all data underlying the findings described fully available, without restriction, and from the time of publication. PLOS allows rare exceptions to address legal and ethical concerns. See the PLOS Data Policy and FAQ for detailed information.	Yes - all data are fully available without restriction

A Data Availability Statement describing where the data can be found is required at submission. Your answers to this question constitute the Data Availability Statement and **will be published in the article**, if accepted.<br><br>**Important:** Stating 'data available on request from the author' is not sufficient. If your data are only available upon request, select 'No' for the first question and explain your exceptional situation in the text box.<br><br>Do the authors confirm that all data underlying the findings described in their manuscript are fully available without restriction?	
**Describe where the data may be found in full sentences. If you are copying our sample text, replace any instances of XXX with the appropriate details.**<br><br>• If the data are **held or will be held in a public repository**, include URLs, accession numbers or DOIs. If this information will only be available after acceptance, indicate this by ticking the box below. For example: *All XXX files are available from the XXX database (accession number(s) XXX, XXX.*).<br>• If the data are all contained **within the manuscript and/or Supporting Information files**, enter the following: *All relevant data are within the manuscript and its Supporting Information files.*<br>• If neither of these applies but you are able to provide **details of access elsewhere**, with or without limitations, please do so. For example:<br><br>*Data cannot be shared publicly because of [XXX]. Data are available from the XXX Institutional Data Access / Ethics Committee (contact via XXX) for researchers who meet the criteria for access to confidential data.*<br><br>*The data underlying the results presented in the study are available from (include the name of the third party*	All relevant data are within the manuscript

and contact information or URL).<br>• This text is appropriate if the data are owned by a third party and authors do not have permission to share the data.<br><br>* typeset	
Additional data availability information:	

Powered by Editorial Manager® and ProduXion Manager® from Aries Systems Corporation

Manuscript

Click here to access/download;Manuscript;Revised Manuscript-Clean.docx

A framework for visualizing and describing city image promotion short video data based on microcube model

Jing He[1,a*], Zhuoluo Yang[2,b] and Jiayi Zhu[1,c]

[1] Institute for Advanced Studies in Humanities and Social Science, Beihang University, Beijing 100183, China
[2] State Key Laboratory of Media Convergence and Communication, Communication University of China, Beijing 100024, China
*Corresponding author: [a]Email: 18610560816@163.com
[b]Email: 287773664@qq.com
[c]Email: bhhejing@buaa.edu.cn

Abstract: Background: The rapid development of media technology and media environment provides rich resources and convenient ways to shape the image of the city. Short video has become an important help to shape the image of the city and build the city brand. How to use short video to shape the image of the city is the key link of urban construction. How: In order to realize comprehensive analysis of short video content, this paper collected a total of 20668 video screenshots for the Douyin platform as data samples, and used Python programming language and Pandas and Matplotlib libraries to process and visualize the data. From the microscopic point of view, a short video data analysis framework of city image promotion based on microcube model is constructed. Objective: To reveal the relationship between video content features and popularity by quantitative and visual methods, and to provide reference for optimizing urban brand promotion strategies. Conclusion: (1) The microcube model shows strong flexibility and applicability in short video content analysis, and can help reveal the complex relationship between short video content characteristics and communication effect. (2) Unexpected events and emotional resonance are key factors in the attractiveness and communication effect of short videos. Reasonable design of scene switching and element interaction significantly enhances the visual impact of short videos.

Keywords: City Image Promotion. Short Video. Micro Perspective. Imagery Elements

1 Introduction

City image promotion has become an important issue in city management and economic development. Especially in today's rapid development of digital media, short video stands out among many communication methods in the field of new media, combining the "mirror" of the city in the form of fragmentation, uniqueness and fluidity, and rapidly spawning a variety of new Internet celebrity cities, which has great influence in attracting potential tourists, and also provides an important platform for the export of urban culture and international brand image. However, the performance of short video propaganda is obviously different in different cities, and there is a lack of systematic methods to evaluate the specific impact of video content on the visibility and communication effect of cities. Therefore, how to analyze short video content from the perspective of data and visualization and provide optimization suggestions for urban publicity strategies has become an important issue that needs to be explored. Most of the existing researches focus on the communication characteristics or creative skills of short video, and lack of quantitative content analysis to analyze the specific role of short video in shaping city image. For example, how to analyze short video content more scientifically and extract the key factors affecting the dissemination effect? Starting from

micro perspective, this study proposes a short video analysis framework to explore the correlation between short video content features and communication effects. This analysis framework can not only provide ideas for the brand communication of Chinese cities, but also provide reference for the optimization practice of urban image publicity under the background of globalization.

2 Related work

At present, domestic and foreign scholars mainly focus on the content production, communication characteristics, development status and ethical dilemmas of short videos, and relatively little research is conducted based on the screen perspective of short videos. After reviewing and summarizing the existing related literature, it is found that short video screen research mainly focuses on six types.

(1) Unexpected events

The advent of the mobile Internet era means the advent of the era of attention economy, where short videos with novel content can get the audience's attention first and content that exceeds the audience's expectation can rapidly seize the market share. Unexpected events refer to unpredictable and unrepeatable scenes that are presented in short videos, becoming key highlights to attract more audience attention. Huang Feng (2021) suggests that the unexpected events in life are irreproducible and will be presented in the short video screen to become a great highlight and gain more attention from the audience. Qin Feng et al. (2022) found that differentiated video material, ups and downs, and innovative elements would reduce viewers' boredom by analyzing short video user comments. M Li et al. (2023) pointed out that content involving unexpected scenes (such as emergencies or unexpected turns) in videos has a much higher forwarding volume than that of ordinary videos. This "surprise effect" suggests that unexpected events play a significant role in attracting attention.

(2) Emotional resonance

Unlike traditional media, where "content is king", new media focus more on "people-oriented", attaching importance to people's emotional expression and emphasizing immersion experience. Emotional resonance involves evoking emotional connections with audience through video content, thus enhancing engagement and loyalty. Cheng Qianqian (2022) proposes that adding regional cultural elements of revolutionary base areas such as dialects in short videos of red documents can create a familiar cultural atmosphere for viewers, reinforce the sense of regional belonging while restoring history, and enhance viewers' immersion and sense of immersion. Huang Xiaoyin (2019) argues that emotion is currently recognized as the basis for sustaining users and is the strongest link between users and the platform, whether it can inspire users' emotional resonance has also become an important indicator to judge whether media products have influence. As the head IP of food short videos, "Eclipse" , uses the cooking process of food to convey the attitude of "living well" and inspires users to identify with and "be in" the emotional level by constructing a specific daily life space. This will lead to the emotional resonance of users. B Wang (2024) found that short videos can significantly increase users' willingness to share by enhancing positive emotional expression, especially in content involving cultural identity and community belonging.

(3) Scene transition

The smallest unit of the video is the shot, and scene transition refers to techniques that enable smooth transitions between consecutive shots to ensure continuity of time and space. According to Sun Zhenhu (2020), the reasonable use of transitions can make the content presentation rational and the story elaboration clear, so that the audience can easily understand and accept it. Yan Hanmei (2022) introduces three types of techniques to achieve transitions from the perspective of screen editing: fluent editing, matching editing and jump cutting, and selects appropriate editing techniques to meet the best

viewing experience of viewers according to different short video production needs. The reasonable use of transitions can also make the news report free from traditional forms. For example, the short video of "One Day Patrol of Civil River Chief" presents the whole "river chief system" activities in a minute with multiple scenes, transitions and angles. Y Zhou (2024) proposed that rapid scene switching can enhance the rhythm of the video, but if the switching is too frequent, it may reduce the viewing comfort of the audience.

(4) Elemental amplification

Elemental amplification involves focusing on details to highlight the characteristics of characters and events. After studying Breakfast China, Fan Yuanyuan found that the short documentary left behind large scenes, used more close ups and close-ups to show people and actions, paid attention to detail control, and presented breakfast such as steaming Fujian Saiki fried buns and fragrant Guangdong Chaoshan pig's blood soup on the small screen to truly show people's living conditions. The short video "Tribute to the People's Police" produced by Quanzhou Radio and Television Station in conjunction with Quanzhou Public Security Bureau contains close-up images of public security police using DNA technology for data comparison and analysis and research, highlighting the professionalism of the people's police and how technology can help criminal investigation work. Y Lin (2022) analyzed close-ups in short videos and found that enlarging detailed elements (such as food texture and architectural decoration) can effectively attract viewers' visual attention and enhance content memory.

(5) Element interaction

In the traditional media period, the picture elements are flat and single, newspaper pictures are a combination of colors, and TV reports use the normalized camera language and transitions to complete the narrative. In contrast, in the new media era, element interaction refers to the use of various visual elements that do not appear in isolation as a single symbol to increase the visual impact in a limited screen. For example, the characters in "The Day of Food" include a Persian cat endowed with a distinct personality in addition to the chef Jiang Laodao, who is the introducer at the beginning of the video, the spectator of the cooking process, and sometimes the taster of the final result. The short video does not set up human emotions, but expresses emotions from the cat's point of view, making the video easy and interesting, but real and natural. Shen Yang (2020) argues that in short video refinement production, the small depth-of-field images captured by the combination of large aperture and telephoto can obtain a sharper perspective and reflect the image texture and ingenuity as it is. MY Shedid (2022) found that in short videos, dynamic interactive scenes (such as the dialogue between natural elements and modern buildings) can enhance the audience's immersion through the integration of multi-modal information.

(6) Screen style

Screen style refers to the overall visual tone and atmosphere of short video, encompassing color grading, lighting, and cinematographic techniques. Different styles can convey specific moods and influence audience perception. Hao Xiaoyuan (2017) took One, Ershang and Pear Video as the research objects and found that the styles of One and Ershang are both inclined to fresh and literary style, and Pear Video presents a variety of styles due to its worldwide patron system. Shen Qingbin (2019) found that most of NHK's documentaries use plain means to pick up images and focus on the communication of live atmosphere. Compared with the BBC, where every shot is perfect, NHK's documentary images are more plain and simple, and low-key and subtle, so he believes that NHK's documentary images as a whole present a rustic style. In the urban documentary Jin Zhi Yang, subdued tones are used to adjust the relationship between light and shadow to outline the scene of the dignified and mysterious urban architectural complex of Taiyuan, Shanxi Province, highlighting the unique historical and humanistic atmosphere of Taiyuan. L Ma (2023) analyzed the picture styles in urban short videos and found that highly consistent and aesthetically rich visual styles are more likely to

establish audience's emotional attachment to video content. For example, fresh-styled videos are significantly more appealing to younger users than other genres.

In addition to the above six parts, scholars at home and abroad have also studied other aspects of picture content, such as multimedia technology, legal copyright, post-production, shooting composition, etc., but literature data are even scarcer. For example, Cui Guobin (2019) explored whether live sports event screens have originality in the sense of film works. Peng (2016) and others introduced the latest progress in video coding of screen content. Wang Zhen (2020), on the other hand, analyzed the principles that should be followed in filming current affairs news TV screens and discussed the filming techniques of different types of TV current affairs news. Zhang Jieliang et al. (2018) explored the method of video conference screen quality evaluation from both subjective and objective aspects.

In summary, this paper obtains city image short video data with geographical location markers, takes the research literature of domestic and foreign research scholars on short video images as a reference basis, combines the presentation patterns of city image propaganda on short video, including content themes, emotional bias, media sources, communication characteristics and dynamic assessment of overseas media, traditional mainstream media communication discourse insight, official media and local self-media synergistic development The content production characteristics of short video images are extracted and initially set as six imagery elements: unexpected events, emotional resonance, scene transformation, element amplification, element interaction, and picture style, as the research dimensions of the data visualization description framework of city image propaganda short video based on the microcube model.

3 Microcube model framework construction

3.1 Unexpected events - Sense of novelty

Taking the micro perspective as the analysis perspective, the video content is refined and a content microcube model is established to encapsulate the complete short video into multiple fragmented short videos according to the time interval, i.e., each small cube encapsulates the video content per unit of time, as shown in Figure 1(a).

Then an assignment operation is performed on each small cube. This operation contains multiple inputs a1, a2 ..... .an (technology elements, food elements, natural landscape elements, cityscape elements, people elements, animal elements, and other imagery elements), an output z, and multiple intermediate parameters (the number of parameters is comparable to the number of time intervals). Among them, a1, a2 ..... .an are set to exist equal to 1 and not exist equal to 0. The intermediate parameter values take values that vary in multiples. A directional arrow indicating the connection can be understood as follows: at the input side, the size of the transmitted signal is a, and there is a change parameter w in the middle of the end, and the signal becomes a*w after several changes.

Let the total duration of the short video be t. After n times of segmentation, the signal size at the output end becomes $a * \left(\frac{w_1}{n} + \frac{w_2}{n} + \cdots + \frac{w_n}{n}\right)$, i.e. $a * \frac{w}{n}$. If the range of $\left|\frac{a*w}{n} - a\right|$satisfies the threshold, the video content display is considered smooth. if the range exceeds the threshold, the video content display is considered transitive and contains the transitive element T. The event that triggers this transitive is called an unexpected event. Figure 1(b) shows the schematic diagram of the occurrence of unexpected events in short videos. 3-4s and 6-7s are both transitions time domain, and the transitions region is visualized by color, and its shades represent the degree of transitions.

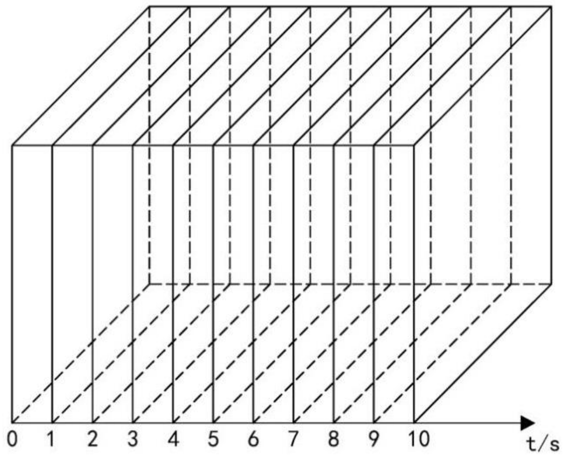

(a) (b)

Figure 1 Refinement analysis of the content presentation module

(a) content cube cut. (b) accidental event microcube visualization model

3.2 Emotional resonance - Sense of immersion

Short videos are in a sense emotional product in the process of communication. Experimentally, we designed an emotion grid chart with nine emotions including moved, praised, funny, happy, neutral, angry, sad, shocked and helpless, and used #EE6363, #EEDFCC, #FFA54F, #FFEC8B, #FFFFF0, #00F5FF, #63B8FF, #00FF7F and #CD69C9 as RGB color controls, respectively. as shown in Figure 2(a).

Then, the emotion grid map is color-matched with the content cube model (neutral to white, not identified as color system, and its fractional video number is set to N), and the result of matching is that the emotion marker is implemented to the content cube model, and the marker color is consistent with the RGB color type in the emotion grid map. Then the complete short video will be divided into multiple color-based fractional short videos A according to the time interval, i.e., each small cube encapsulates the emotional content per unit time. Among them, the color matching degree $M = \frac{A-N}{A}$, when M>30%, is strong in emotional substitution. Figure 2(b) is a schematic diagram of color matching degree in the city image video with M=50%, 2-4s, 7-8s, 8-9s, and three emotions of happy, shocked, and helpless, respectively.

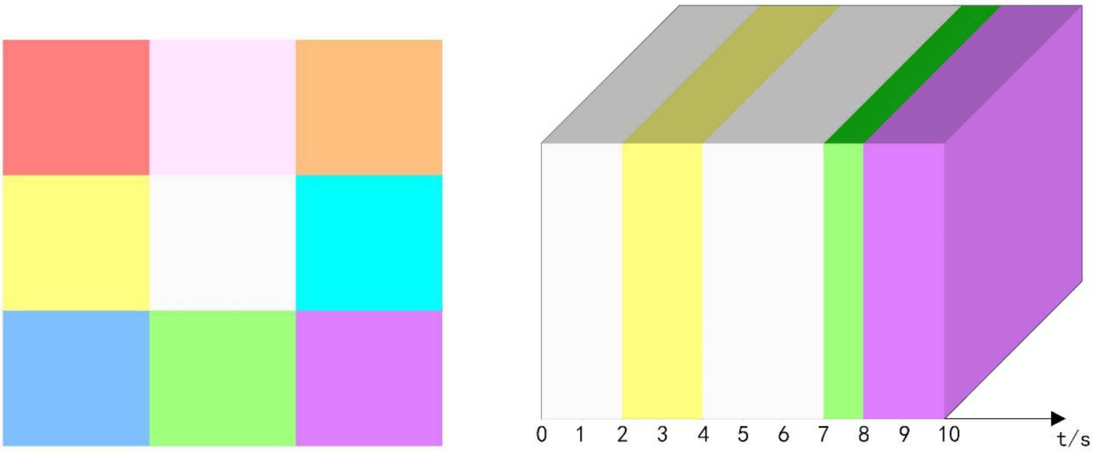

(a) (b)

Figure 2 Refinement analysis of emotional carryover

(a) emotional grid diagram. (b) color matching results

3.3 Scene transition - Sense of Following

Scene transitions imply the existence of a relatively large change in temporal or spatial relationships. Time is divided into broadcast time, event time, and narrative time. space is divided into physical space and psychological space. physical space is the display space of actual transitions, and psychological space is the space adjusted with the ups and downs of the storyline. The TS level transition scenes include T1, T2, T3, S1, S2 and Tn/Sn. Among them, T1, T2, and T3 represent the corresponding broadcast time conversion, event time conversion, and narrative time conversion. S1 and S2 represent the corresponding physical space conversion and mental space conversion. and Tn/Sn represents the hybrid conversion of time and space connection. The TS level conversion domain model describes the abstract static structure of the system, and only after matching the dynamic behavior can the complete representation of the system be completed.

In the TS level conversion scenario, two typical metrics, switching correctness and real-time, are selected. Switching correctness requires the following 2 conditions to be discerned: first, based on the completion of the short video to be able to enter successfully different scene states. second, traversing the frame state paths of all standard samples in the short video. Real-time metrics require not only the complete short video playback to respond within a determined time, but also to ensure the correctness of the response. In this scenario, once the relevant time constraint is violated, it will not only affect the efficiency of scene transition, but may also cause the coherence degree of video playback.

Analysis of scene conversion for the video after content refinement: let the total propagation time of the short video be t. After n times of segmentation, visualize each scene as a different color cube within the unit time, and the color switching represents the conversion of the scene, and let the number of scene switching be q. In Fig. 3(a) ~ Fig. 3(d), q is equal to 3, 2, 0, and 6, respectively.

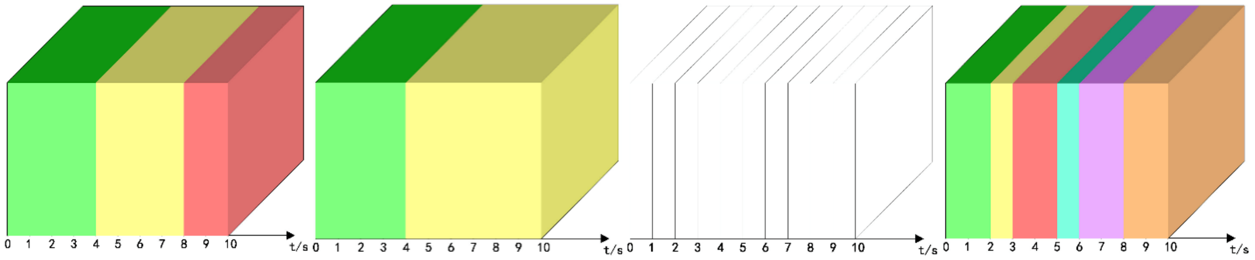

(a) (b) (c) (d)

Figure 3 Scene transition microcube visualization model (a)scene transitioned three times. (b) scene transitioned twice. (c) no scene transition. (d) scene transitioned six times

3.4 Amplification of elements - Sense of in-depth

In fact, for the short video platform, even the city image promotion is shown as "content production", which usually contains one or more imagery elements such as technology elements, food elements, natural landscape elements, urban landscape elements, people elements, animal elements, etc. We designed the imagery elements grid and used #00FF7F, #FFEC8B, #EE6363, #00F5FF, #EE82EE, #FFA54F as RGB color controls, as shown in Figure 4(a).

The video content is further refined to establish the tiny content cube model, then each fragmented short video is further divided into 100 sub-cubes equally, i.e., each sub-cube encapsulates the video content per unit time and per unit screen, as shown in Figure 4(b). Let each sub-cube be 1 unit, when cube cutting is performed on the short video sample data, one or more of the imagery elements may appear on the video screen

per unit of time. The percentage of occupied screens for each imagery element is quantitatively analyzed and visualized with different colors. Elements with screen occupancy greater than 30% and scene time greater than 3s are extracted and identified as amplified elements, set as E. Amplification of elements is relative to each other and there is no gradual change over time or other measure. Representation = f(e1, e2, . . , e3), where e is the elements that characterize the short video content. Figure 4(c) shows four amplification elements, where 0-4s are cityscape elements, 6-8s and 10-11s are food elements, and 8-9s and 13-14s are technology elements.

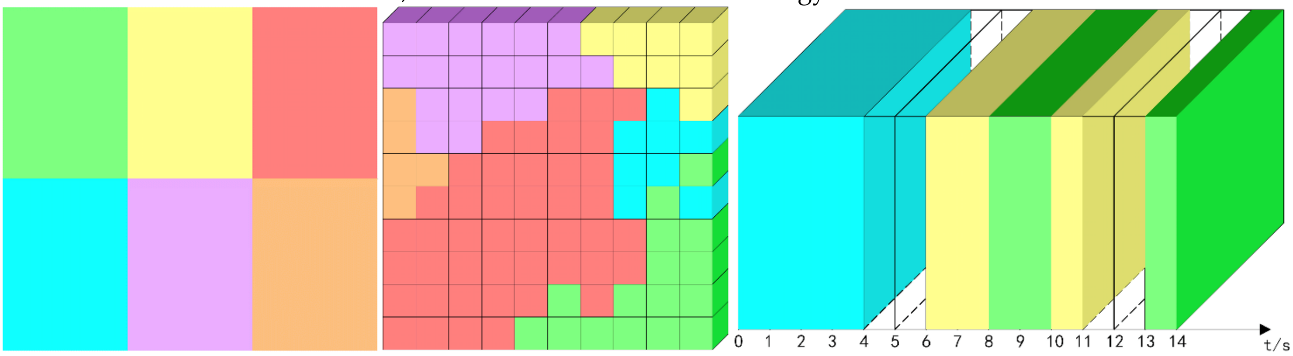

(a) (b) (c)

Figure 4 Refinement analysis of element amplification (a) color grid diagram of imagery elements. (b) sub-cube model. (c) this sample data contains 4 amplified elements

3.5 Element interaction - Sense of dynamism

The analysis of the amplified elements continues with further decomposition of elements of the same category. The decomposition is based on the principle of interaction, i.e., when there is interaction between elements of the same category, only then the element continues to be decomposed into multi-category interactive elements, and the original element is represented by OE and the decomposed element is represented by DE. The visualization color of DE is determined by the visualization color of OE as a sub-color family of OE. Let the total propagation time of the short video be t. After n times of segmentation, the microcubes with different color particles are visualized according to the element categories in each subcube per unit time, among which, the original element of technology OE technology is original green, the original element of food OE food is original yellow, the original element of natural landscape OE natural landscape is original red, the original element of urban landscape OE city is original cyan, and the character The original element OE characters is the original purple, and the original element OE animals is the original orange. When the imagery element is judged to be an interactive element, OE technology is decomposed into OE technology 1 + OE technology 2, OE cuisine is decomposed into OE cuisine 1 + OE cuisine 2, OE natural landscape is decomposed into OE natural landscape 1 + OE natural landscape 2, OE cityscape is decomposed into OE cityscape 1 + OE cityscape 2, OE character is decomposed into OE character 1 + OE character 2, and OE animal is decomposed into OE animal1+OE animal2, as shown in Figure 5(a).

Let the total propagation time of the short video be t. After n times of segmentation, the different sub-cubes per unit time are decomposed into multiple micro-cubes with different colors, as shown in Figure 5(b). Firstly, the amplified elements are judged according to the method described in 4.4, and then the decomposition analysis of the elements is performed on the amplified elements, and the color decomposition is recorded, and Figure 5(c) demonstrates the decomposition rate d=70%.

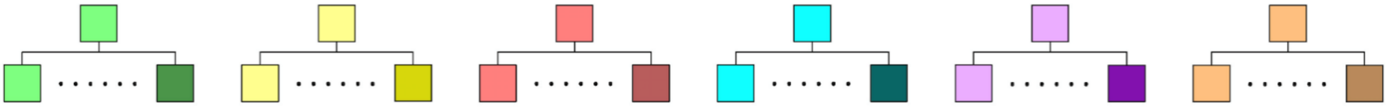

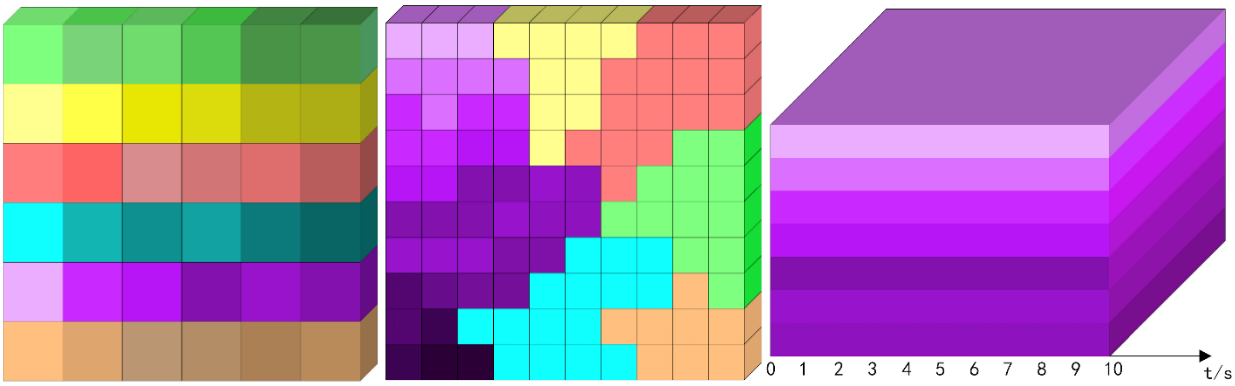

(a) (b) (c)

Figure 5 Refinement analysis of element interactions (a) color grid diagram of the decomposition of imagery elements. (b) microcube model of character element interactions. (c) decomposition of 7 interactions of character elements

3.6 Screen style - Main color

Each image in the video can be abstracted into three forms: point, line and surface, and the effective presentation of these forms are the elements. The above decomposition of different elements has been carried out, and the decomposed elements are the basic framework to form the microcube model. However, the video screen is often more than a simple combination of individual elements, and sometimes involves an effective merging of elements to form the style of the overall video screen. A grid diagram of the main colors of the screen style was designed, and #0000FF, #BEBEBE, #FFFACD, #6495ED, and #FFC0CB were used as RGB color controls for dark, gray, light, medium, and bright tones, respectively, as shown in Figure 6(a). Figure 6(b) shows five main colors of the screen, among which, 0-2s and 4-5s are bright tones, 2-4s are gray tones, 5-7s are dark tones, 7-8s are light tones, and 8-10s are medium tones.

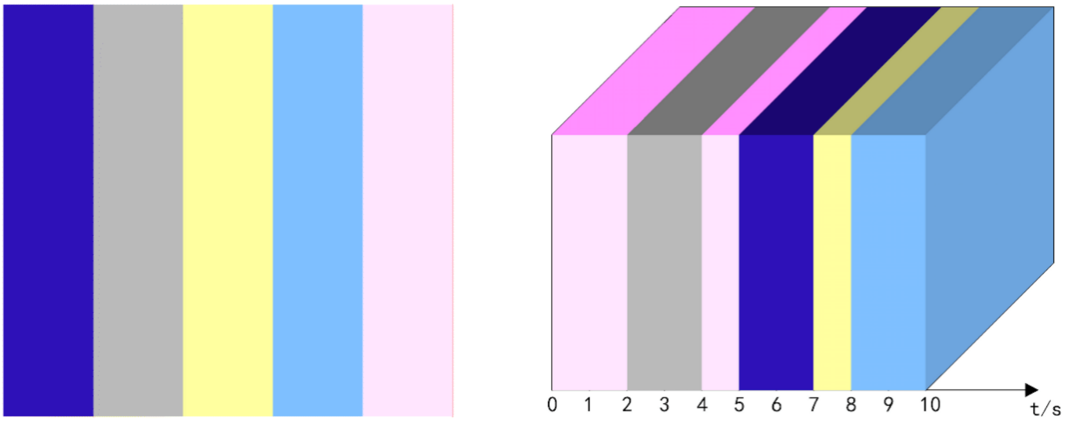

(a) (b)

Figure 6 Refinement analysis of picture styles (a)color grid diagram of primary colors. (b) visual framework of 5 primary colors

4 Analysis of cases

There are three levels in the short video communication effect rating index model: the criterion level, the sub-criterion level and the solution level. The criterion layer is based on the hotness, breadth and depth of communication. the sub-criteria layer sets the second-level indicators based on the media hotness, fan hotness, search hotness, reputation of each network platform, content acceptance, cross-domain linkage, crowd reach, thematic distinctness and thematic diversity. the solution layer corresponds to the

sub-criteria layer and refines the relevant indicators. After applying the index model to evaluate the communication effect of the short videos of Beijing city image propaganda, the absolute hotness (H) concept is used to calculate the hotness value of the short videos of online celebrities using a one-year time period as the time period of attention: H=(xCa+yCb+zCc)(1+Cd). Where H denotes the absolute hotness value. Ca denotes the value calculated by the propagation hotness criterion layer. Cb denotes the value calculated by the propagation breadth criterion layer. Cc denotes the value calculated by the propagation breadth criterion layer. Cd denotes the account quality of the video publisher (depending on the account quality of the video publisher, the value is between 0 and 1). x and y denote the factor weights (depending on the importance of the factor, the value is between 0 and 1). . Accordingly, the city image promotion short videos in different heat ranges are categorized. Appendix I shows the design model of short video hotness index system.

Based on the hotness value of the short videos of Netflix, this experiment selected a total of 1200 short videos of popular city image propaganda short videos from the TikTok platform with likes >1w and retweets >0.8w and non-popular city image propaganda short videos with likes <1w and retweets <0.8w within the second half of 2019, and a total of 20,668 video screenshots were obtained after the screen draw seconds per second, and ensure that the video content includes geolocation tags and a clear city promotion theme. To ensure the authenticity and representativeness of the video content, the research team manually reviewed all selected videos and cross-verified them with the metadata provided by the platform, such as upload date, tag and post account number, to eliminate possible noise data (In the obtained videos, all content is strictly in accordance with the data use policy, only for academic research, and does not involve personal privacy information). Table 1 shows the number of microcubes and the number of video screenshots per second in the framework of visualization description of city image promotion short video data based on microcubes model for popular cities and non-popular cities. Further, plot the relationship heat matrix for popular and non-popular cities in the following dimensions: the number of unexpected microcubes, number of unexpected frames, number of emotion resonance microcubes, number of positive emotion microcubes, number of negative emotion microcubes, number of emotion resonance frames, number of positive emotion frames, number of negative emotion frames, number of scene transition microcubes, number of scene transition frames, number of elemental amplification microcubes, number of elemental amplification frames, number of microcubes with at least two amplification elements, number of frames with at least two amplification elements, number of elemental interaction microcubes, number of elemental interaction frames, number of gray tone microcubes, number of gray tone frames, number of dark tone microcubes, number of dark tone frames, number of bright-toned microcubes, number of bright-toned frames, number of mid-toned microcubes, number of mid-toned frames, number of light-toned microcubes, and number of light-toned frames. The relationship heat matrices are plotted in terms of the number of frames, the number of bright-tone microcube models, the number of bright-tone pumping frames, the number of mid-tone microcube models, the number of mid-tone pumping frames, the number of light-tone microcube models, and the number of light-tone pumping frames, as shown in Figure 7, each using the same

emerald green color space, but using a different color mapping. The greener the color represents the stronger the correlation between the two factors, and is dark green when both are the same element (as shown in the diagonal of the matrix), indicating a 100% correlation. The redder the color means the less correlation between the two factors. Comparing the two heat matrix plots, it is found that there are significantly more green color blocks in popular cities (a) than in non-popular cities (b), and the green color concentration is greater. while there are significantly more red color blocks in non-popular cities (b) than in popular cities (a), "more than the number of microcube models of both elements" The number of "microcubes with more than two elements" is negatively correlated with nearly half of the other elements, and the number of brightly colored second frames is significantly negatively correlated with the number of brightly colored second frames. The above analysis shows that there is a correlation between the intention elements of short videos from popular cities, i.e., there may be multiple intention elements in the same picture, while the intention elements of short videos from non-popular cities have a weaker relationship with each other, and the intention elements in the video picture are relatively single.

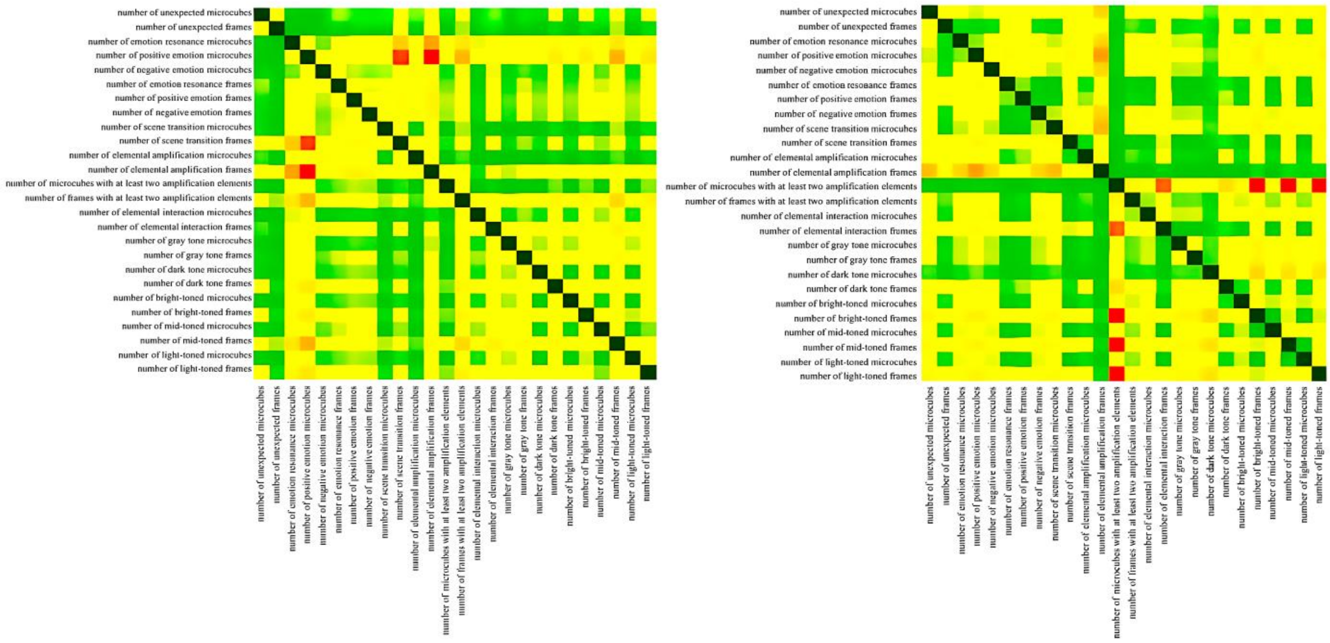

(a) (b)

Figure 7 Relationship heat matrix diagram of short video screen visualization relationship heat matrix display of sample data for popular cities. (b) relationship heat matrix display of sample data for non-popular cities

Due to space limitation, only 144 city popular short video microcubes models with 2393 city popular short video screenshots and 123 city non-popular short video microcubes models with 1834 city non-popular short video screenshots are shown here. To clearly demonstrate that the microcube model-based descriptive framework for visualizing city image promotion short video data can be used to perform descriptive analysis of content abstraction of popular and non-popular city short videos occurring in a time series, a stack of microcube models is used for presentation.

Table 1 Overview of the microcube model constructed for popular/non-popular cities

popular cities	Beijing	Chen gdu	Guang zhou	Nan jing	N an ni ng	Shang hai	Sh en zh en	Wu han	Xi an	Zh ang jiaji e	Chan g sha	Chon g qing	To tal
Number of microcube models	12	12	11	13	13	10	13	12	13	11	11	13	14 4
Number of video frames in seconds	216	205	205	232	21 6	149	22 0	186	21 0	154	182	218	23 93

non-popular cities	Alasha nmeng	Bao ding	Bei jing	Chen gdu	Fo sh an	Guang zhou	Ha ng zh ou	Shan ghai	Sh en zh en	Xia n	Zheng zhou	Chon gqing	To tal
Number of microcube models	13	13	9	7	11	10	11	10	10	9	10	10	12 3
Number of video frames in seconds	211	182	136	107	15 7	140	16 4	154	17 0	135	131	147	18 34

4.1 Visualization results of unexpected events of short videos based on microcube model

Based on the display data, the number of microcube models containing unexpected events and the number of video pumping second frames were recorded as shown in the following table.

Table 2 Number of microcube models containing unexpected events and number of video pumping seconds frames

	Popular city image short video	Non-popular city image short video
Number of microcube models containing element T	106	39
Number of video frames in seconds	408	123

The number of microcube models containing element T in the popular city image videos is 106, and the number of video second draws is 408, while the number of microcube models containing element T in the non-popular city image videos is 39, and the number of video second draws is 123.

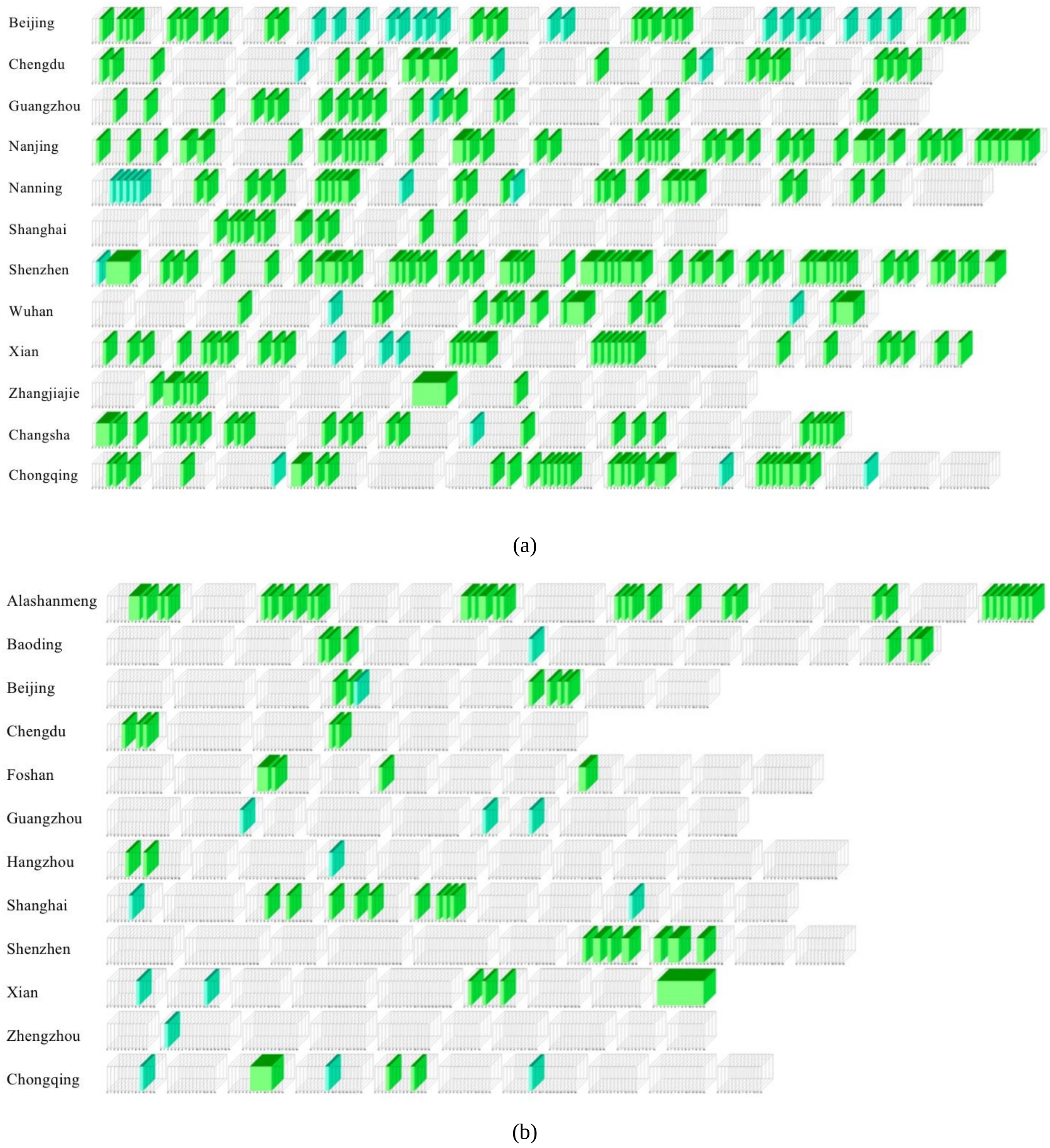

Figure 8 Accidental event stacking graph based on microcube model sample case of popular city image short video. (b) sample case of non-popular city image short video

4.2 Visualization results of emotional resonance of short videos based on microcube model

Based on the display data, the number of microcube models containing the nine emotions and the number of video pumping second frames were recorded as shown in the table below.

Table 3 The number of microcube models containing emotional elements and the video pumping second frames

	Popular city image short video	Non-popular city image short video
Number of microcube models containing emotional elements	69	35
Color matching of total model M1	47.92%	28.46%
Color matching of total emotion M2	9.21%	5.41%
Number of microcube models involving positive emotions	47	32
Color matching of total model M1	32.64%	26.02%
Color matching of total emotion M2	61.84%	91.43%
Number of microcubes models involving negative emotions	29	5
Color matching of total model M1	20.14%	4.07%
Color matching of total emotion M2	38.16%	13.51%
Number of microcubes models involving both positive and negative emotions	7	2
Color matching of total model M1	4.86%	1.63
Color matching of total emotion M2	9.21%	5.41%

The number of microcubes containing emotional elements in the urban popular short videos was 69, and the color matching degree of the total model M1=47.92%. Among them, the number of microcubes involving positive emotions is 47, the color matching degree of the total model M1=32.64%, the color matching degree of the total emotion M2=61.84%. the number of microcubes involving negative emotions is 29, the color matching degree of the total model M1=20.14%, the color matching degree of the total emotion M2=38.16%. the number of microcubes involving both positive and negative emotions is 7. The number of microcubes involving both positive and negative emotions is 7, the color matching degree of total models M1=4.86%, and the color matching degree of total emotions M2=9.21%.

The number of microcubes containing emotional elements in the non-popular city image videos is 35, and the color matching degree of the total model is M=28.46%. Among them, the number of microcubes involving positive emotions is 32, the color matching degree of total models M1=26.02%, the color matching degree of total emotions M2=91.43%. the number of microcubes involving negative emotions is 5, the color matching degree of total models M1=4.07%, the color matching degree of total emotions M2=13.51%. the number of microcubes involving both positive and negative emotions The number of microcubes involving both positive and negative emotions was 2, the color matching degree of the total model was M1=1.63%, and the color matching degree of the total emotion was M2=5.41%.

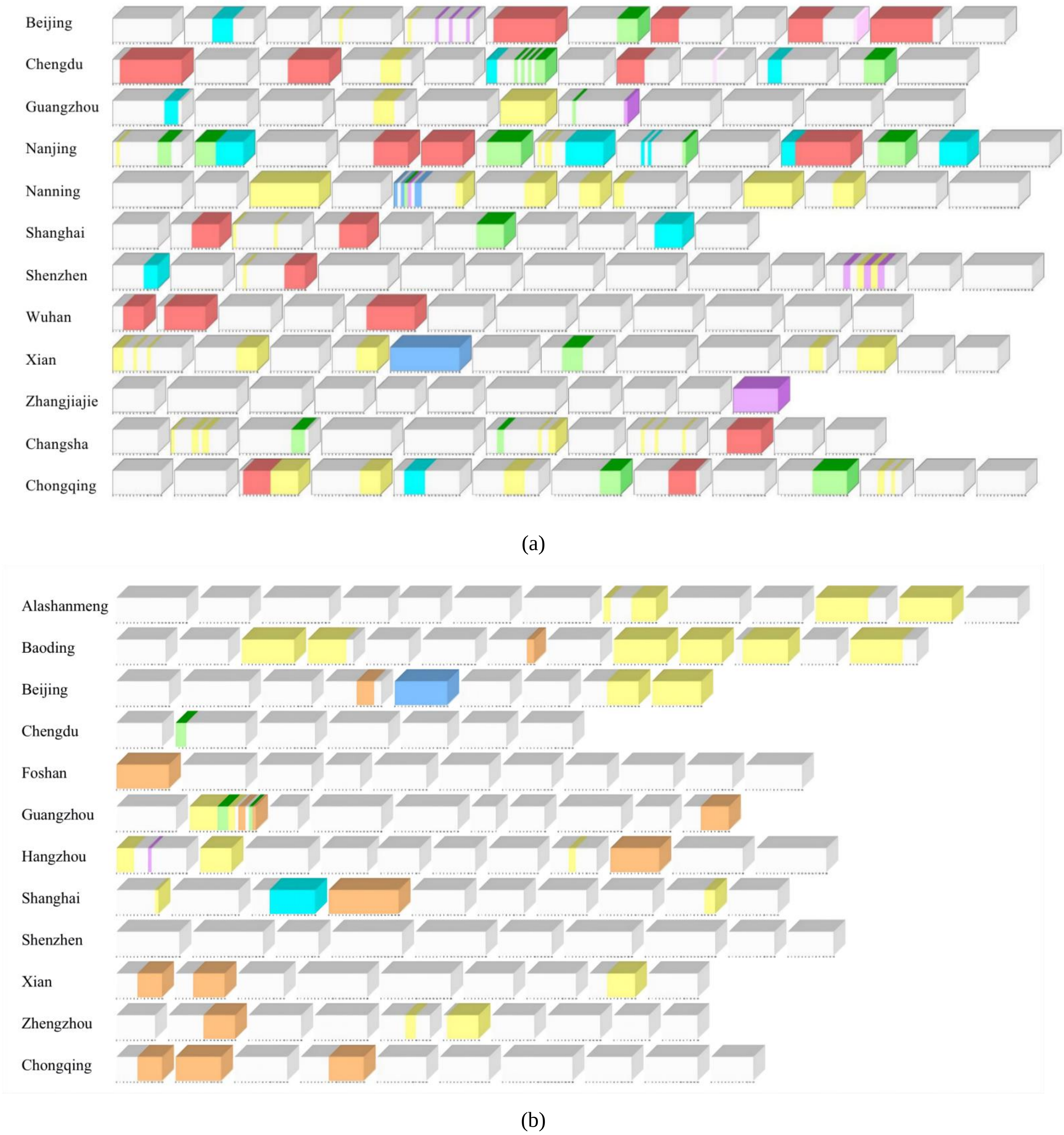

Figure 9 Emotional resonance stacking diagram based on microcube model (a) sample case of popular city image short video. (b) sample case of non-popular city image short video

4.3 Visualization results of scene transition of short videos based on microcube model

Based on the display data, the number of microcube models containing scene transitions and the number of video pumping seconds frames were recorded as shown in the table below.

Table 4 Number and percentage of microcube models containing scene transformation

	Popular city image short video	Non-popular city image short video
Number/proportion of microcube models containing element q	91/67.36%	26/21.14%
Number of microcube models with q=2/percentage	14/10.37%	3/2.44%
Number of microcube models with q=3/percentage	18/13.33%	7/5.69%
Number of microcube models with q=4/percentage	26/19.26%	6/4.88%
Number of microcube models with q=5/percentage	13/9.63%	5/4.07%
Number of microcube models with q=6/percentage	8/5.93%	1/0.81%
Number of microcube models with q=7/percentage	8/5.93%	2/1.63%
Number of microcube models with q=8/percentage	3/2.22%	0/0%

The number of microcube models containing element q in popular city image videos is 91, accounting for 67.36%. among them, the number of microcube models with q=2 is 14. the number of microcube models with q=3 is 18. the number of microcube models with q=4 is 26. the number of microcube models with q=5 is 13. the number of microcube models with q=6 is 8. the number of microcube models with q=7 is The number of microcubes is 8. the number of microcubes with q=8 is 3.

The number of microcube models containing element q in the non-popular city image videos is 26, accounting for 21.14%. among them, the number of microcube models with q=2 is 3. the number of microcube models with q=3 is 7. the number of microcube models with q=4 is 6. the number of microcube models with q=5 is 5. the number of microcube models with q=6 is 1. the number of microcube models with q=7 is 2. The number of models is 2.

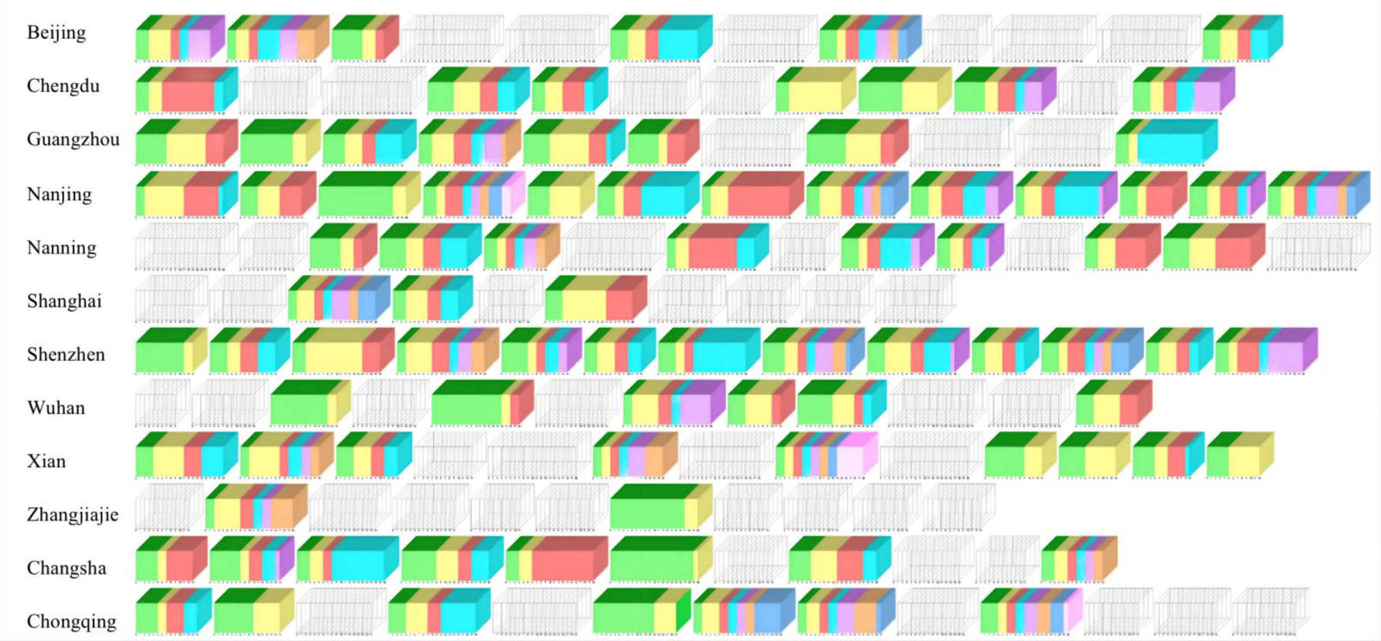

(a)

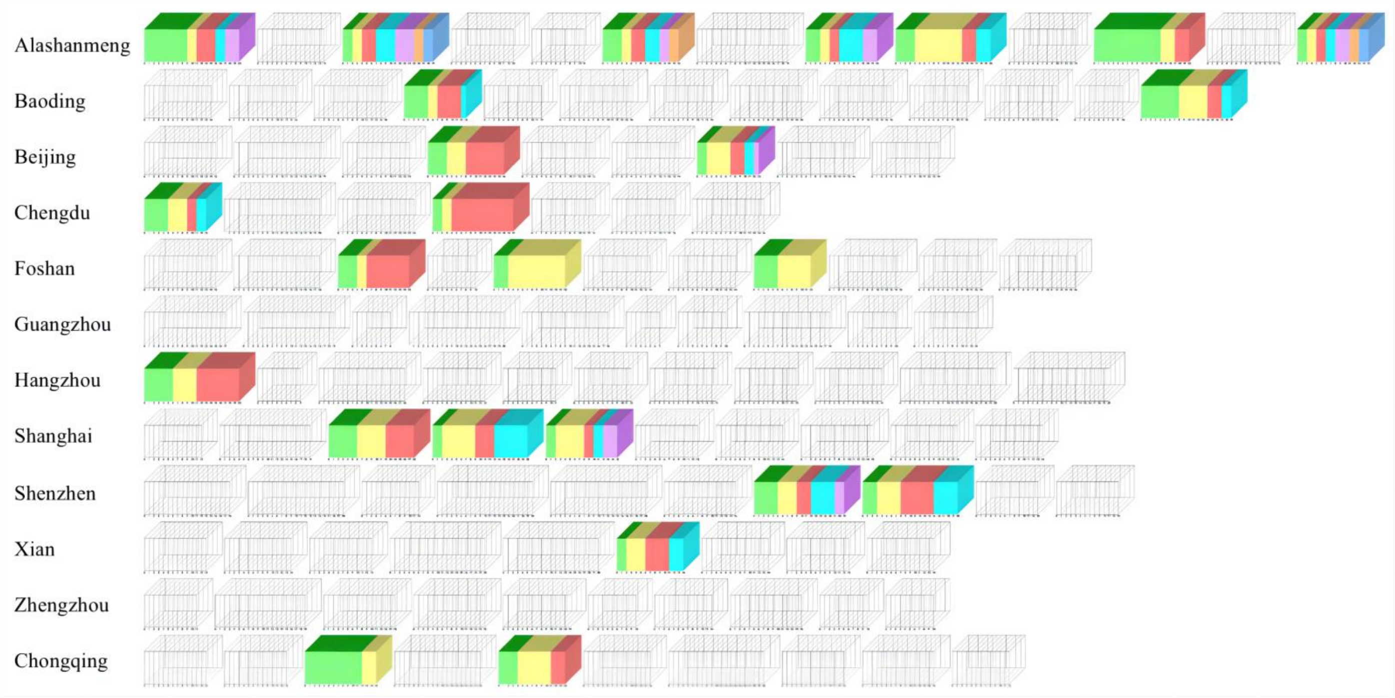

(b)

Figure 10 Scene transformation stacking diagram based on microcube mode sample case of popular city image short video. (b) sample case of non-popular city image short video

4.4 Visualization results of amplification of elements based on microcube model

Based on the display data, the number of microcube models containing the six types of imagery elements and the number of video pumping seconds frames were recorded as shown in the table below.

Table 5 Number of microcube models containing intentional elements

	Popular city image short video	Non-popular city image short video
Number of microcubes containing imagery elements	141	116
Number of microcubes with one type of imagery	93	104
Number of microcubes with two or more types of imagery	48	12
Number of microcubes with technological elements	3	0
Number of microcubes with food elements	33	0
Number of microcubes with natural landscape elements	21	6
Number of microcubes with urban landscape elements	74	25
Number of microcubes with human elements	66	89
Number of microcubes with animal elements	4	0

The number of microcubes containing imagery elements in popular city image videos is 141, the number of microcubes containing one type of imagery elements is 93, and the number of microcubes containing two or more types of imagery elements is 48. Among them, the number of microcubes involving technology elements is 3, the number of microcubes involving food elements is 33, the number of microcubes involving natural landscape elements is 21, the number of microcubes involving urban landscape elements

is 74, the number of microcubes involving people elements is 66, and the number of microcubes involving animal elements is 4.

The number of microcubes containing imagery elements in the non-popular city image videos is 116, the number of microcubes containing one type of imagery elements is 104, and the number of microcubes containing two or more types of imagery elements is 12. Among them, the number of microcubes for urban landscape elements is 25, the number of microcubes for people elements is 89, and the number of microcubes for natural landscape elements is 6.

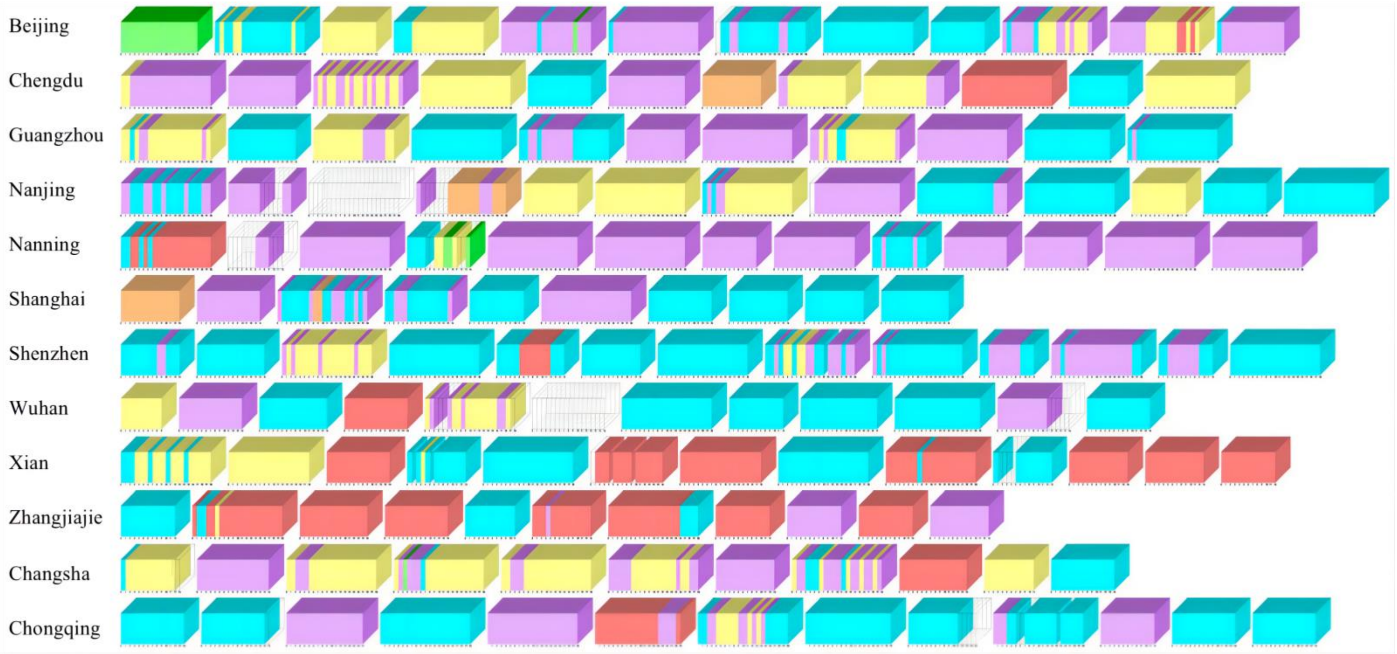

(a)

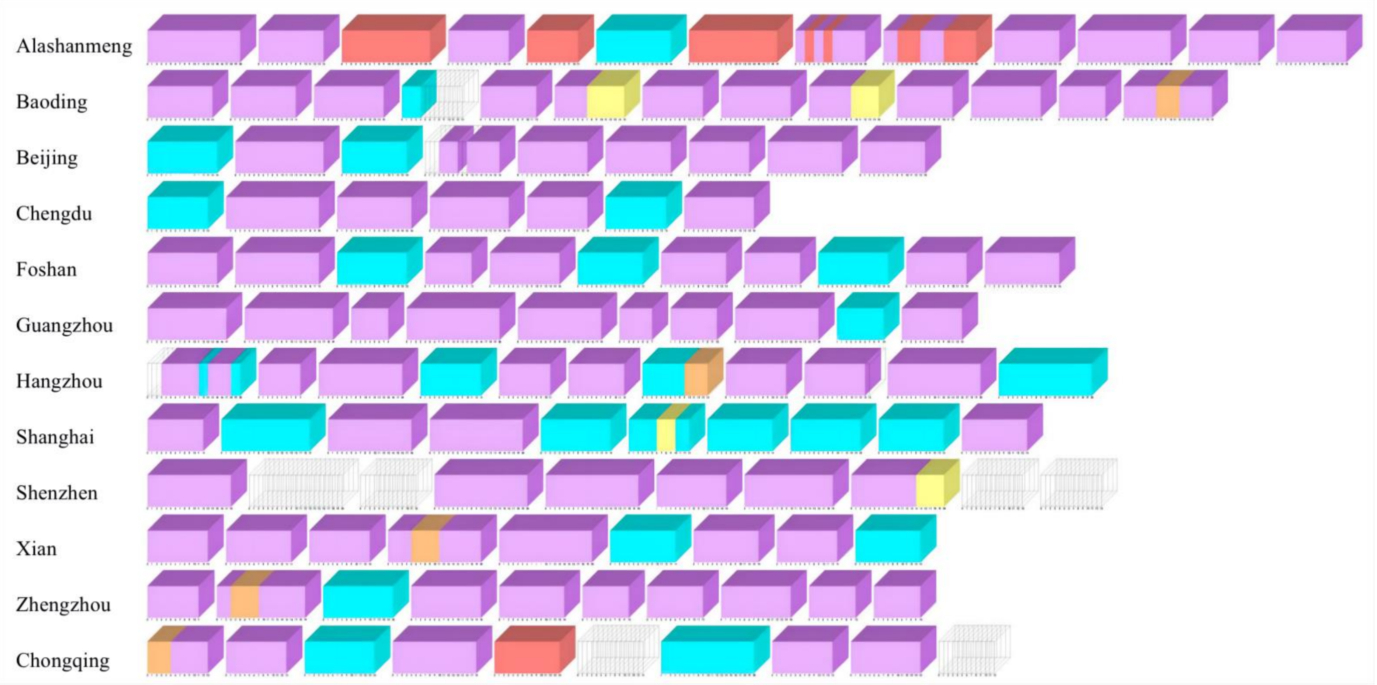

(b)

Figure 11 Amplified stacking diagram of elements based on microcube model sample case of popular city image short video. (b) sample case of non-popular city image short video

4.5 Visualization results of element interaction of short movies based on microcube model

Based on the display data of element interactions, the number of microcube models containing six types of imagery element interactions and the number of video pumping second frames were recorded as shown in the following table.

Table 6 Number of microcube models containing element interactions and number of video pumping seconds frames

	Popular city image short video	Non-popular city image short video
Number of microcube models with element interaction	17	9
Video draw seconds frames (sheets)	150	89
Number of microcube models with dynamic interaction of elements of a certain type of imagery = 2	10	8
Video second draws (sheets)	96	67
Number of microcube models with dynamic interactive subjects > 2 for a certain type of imagery elements	7	2
Video second draws (sheets)	54	22

The number of microcubes containing interactive elements in the popular city image videos is 17, and the number of video frames is 150. Among them, the number of microcubes with dynamic interactive elements equal to 2 is 10, and the number of video frames is 96. the number of microcubes with dynamic interactive elements greater than 2 is 7, and the number of video frames is 54.

The number of microcubes containing interactive elements in the non-popular city image videos is 9, and the number of video frames is 89. Among them, the number of microcubes with dynamic interactive elements of a certain type is equal to 2 is 8, and the number of video frames is 67. the number of microcubes with dynamic interactive elements of a certain type is greater than 2 is 2, and the number of video frames is 22.

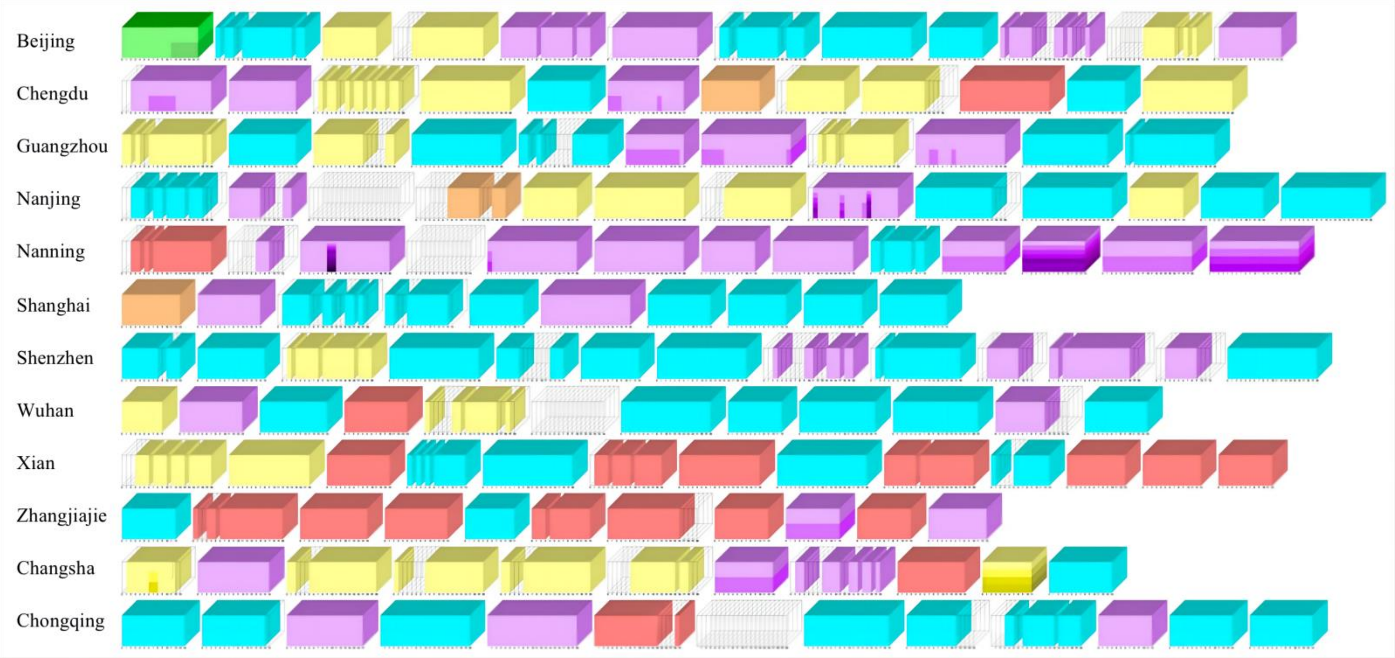

(a)

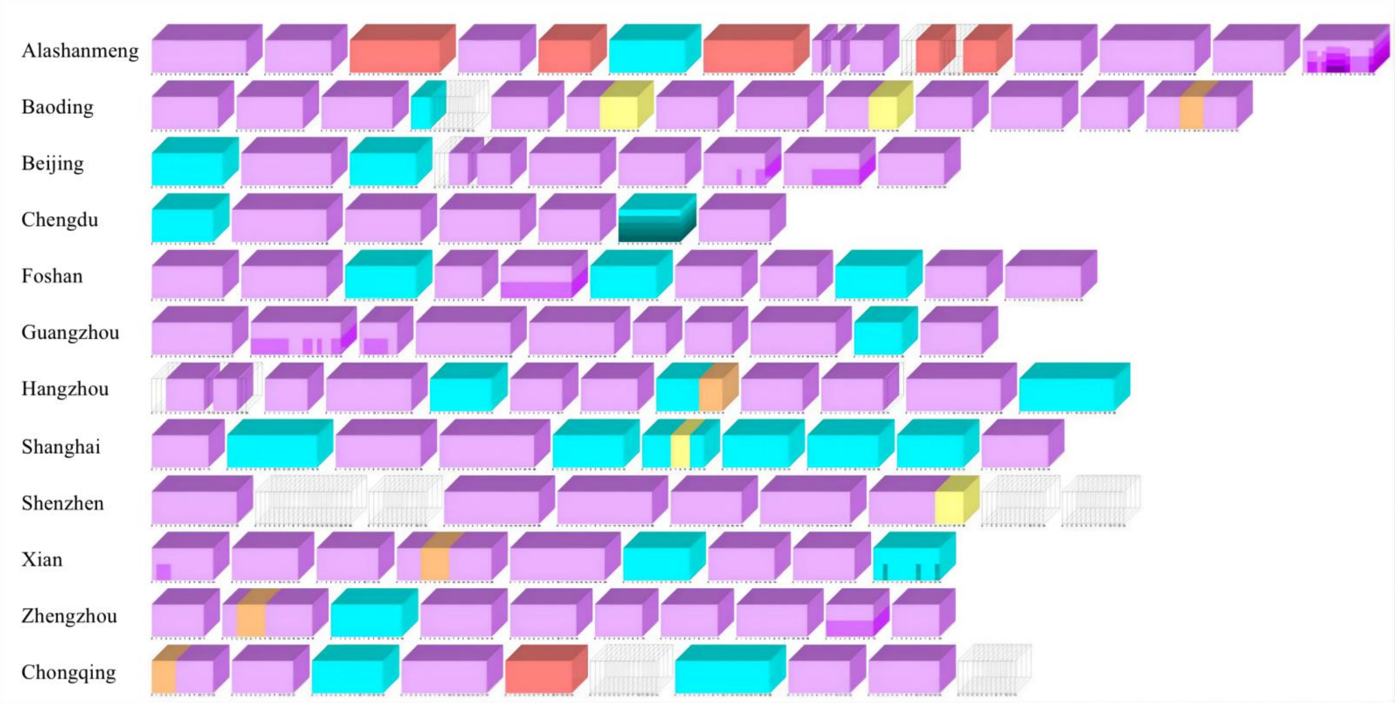

(b)

Figure 12 Element interaction stacking diagram based on microcube model (a) sample case of popular city image short video. (b) sample case of non-popular city image short video

4.6 Visualization results of short video screen style based on the microcube model

Based on the display data, the number of microcube models containing 5 types of hues and the number of video pumping seconds frames were recorded as shown in the table below.

Table 7 Number of microcube models containing unexpected events and number of video pumping seconds frames

	Popular city image short video	Non-popular city image short video
Number of microcube models with gray tones	31	25
Number of video frames in seconds (sheets)	75	26
Number of microcube models containing dark tones	43	11
Video frames (sheets)	391	128
Number of microcube models with bright tones	42	42
Video frames (pictures)	369	556
Number of microcube models with mid-tone colors	74	31
Video frames (pictures)	931	452
Number of microcubes with light tones	47	31
Video frames (sheets)	429	428

The number of microcubes with gray tones in the popular city image videos is 31, and the number of video frames is 75. the number of microcubes with dark tones is 43, and the number of video frames is 391. the number of microcubes with bright tones is 42, and the number of video frames is 369. the number of microcubes with medium tones is 74, and the number of video frames is 931. and the number of microcubes with light tones

is 47, and the number of video frames is 429. The number of microcube models in lighter tones is 47 and the number of video frames is 429.

The non-popular city image videos contain 25 gray-toned microcubes and 26 video frames, 11 dark-toned microcubes and 128 video frames, 42 bright-toned microcubes and 556 video frames, 31 medium-toned microcubes and 452 video frames, and light-toned microcubes and video frames. The number of light-toned microcubes is 31 and the number of video frames is 429.

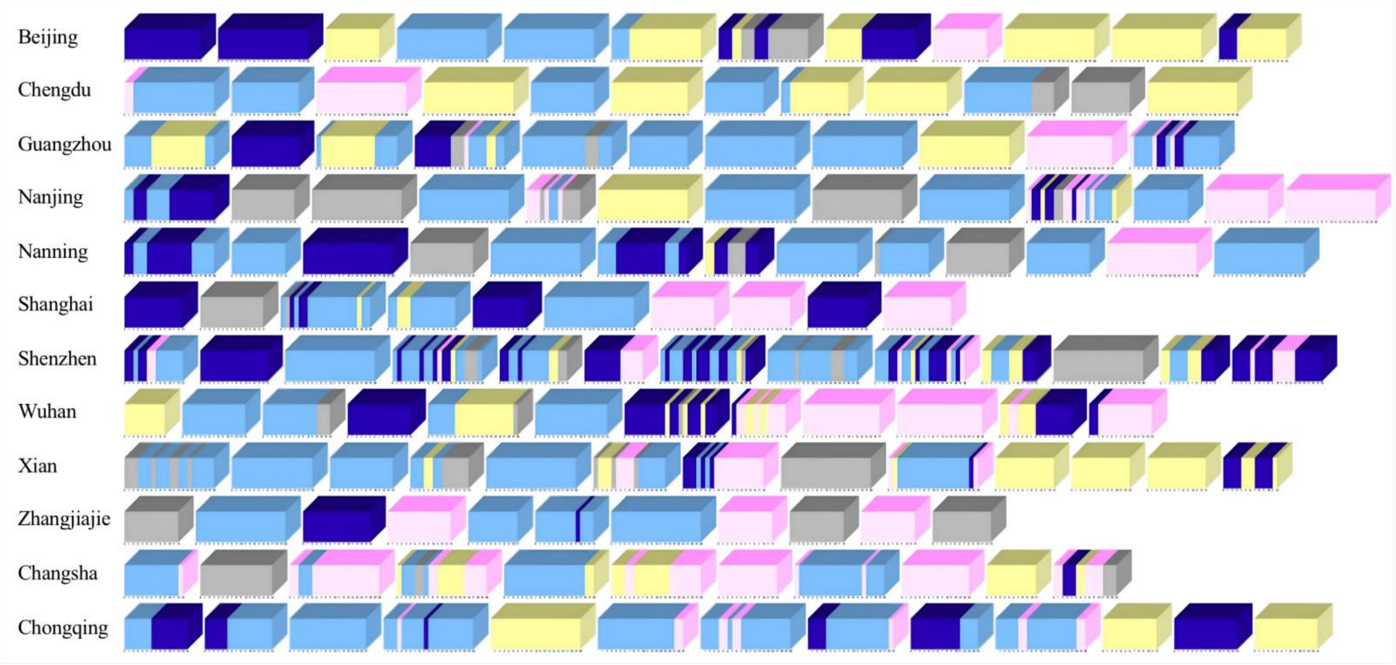

(a)

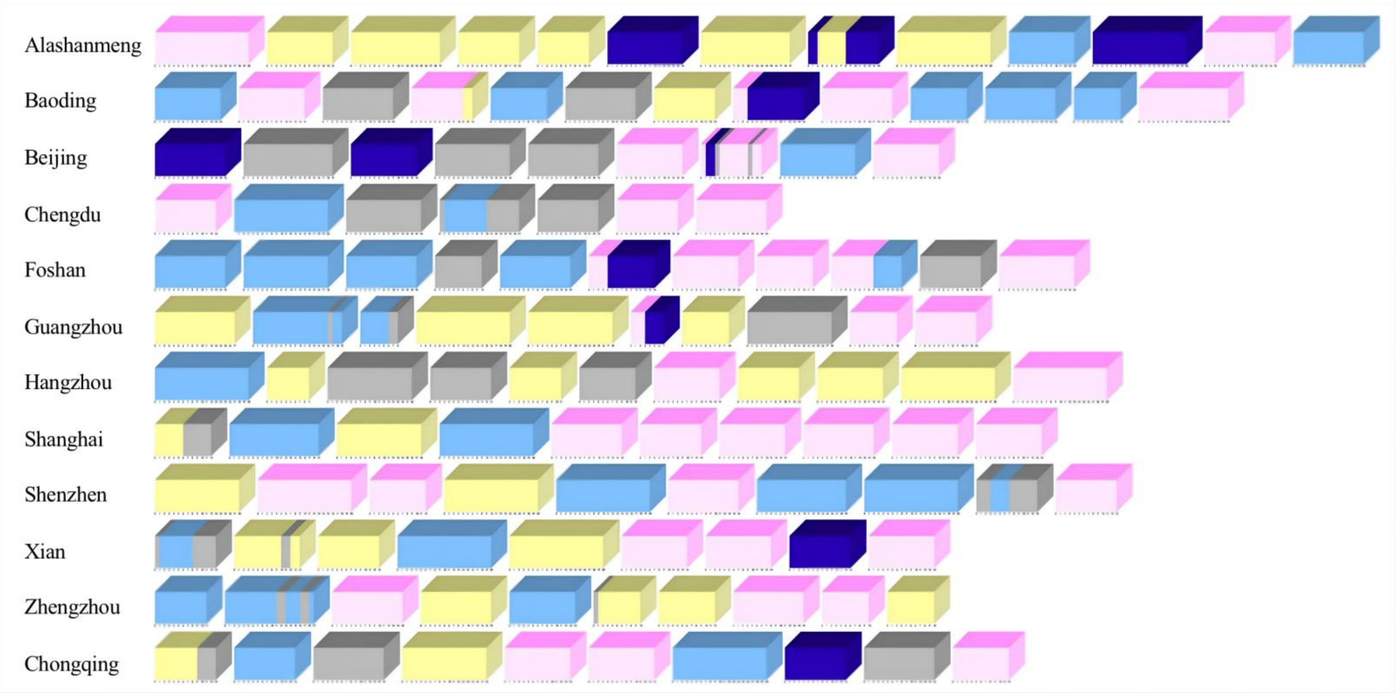

(b)

Figure 13 Screen style stacking diagram based on microcube model sample case of popular city image short video. (b) sample case of non-popular city image short video

6 Conclusion and Discussion

Based on the microcube model analysis framework of TikTok platform data, this study explores the relationship between the content characteristics of short videos and the publicity effect of city images. The research results can not only reveal the influence mechanism of the content structure of short videos, but also provide practical guidance for the production of short videos of urban propaganda, especially in the context of strengthening the communication effect of urban brands. The research shows that: (1) the characteristics of short video content significantly affect the publicity effect of city image: dynamic features (such as unexpected events, scene switching) and static features (such as element amplification and picture style) in short video content have an important impact on the communication effect. For example, short videos with unexpected events are more likely to attract viewers' attention than short videos without unexpected events. Content with positive emotional resonance significantly increased the willingness to watch and share short videos. This suggests that creators can improve the appeal and reach of short videos by optimizing these features. (2) Emotional resonance is positively correlated with popularity: the emotional expression of short videos directly affects its communication effect. The higher the proportion of positive emotion (such as happiness, touching), the higher the popularity of the short video. Negative emotions (such as sadness and anger) have a lower number of microcubes and tend to be less hot. This shows that the city image publicity should pay attention to the expression of positive emotions to enhance the attractiveness of short videos. (3) Reasonable scene switching and diversified elements can enhance the communication effect: the number and logic of scene switching in short videos have a significant impact on the audience's viewing experience. Too little scene switching will lead to monotonous content, while too much will increase the burden of viewing. In addition, multi-element interaction (such as the combination of natural landscapes and human activities) can enhance the visual impact and increase the audience's interest in the image of the city.

Although this study provides a new perspective and tool for short video content analysis, it also has the following limitations: (1) Limitations of data samples: This study mainly focuses on short city image videos on the TikTok platform as the analysis object, and the sample sources are relatively concentrated, which fails to fully cover diverse cultural contexts. Future research can be extended to other platforms (such as Instagram, YouTube) and more international cities to verify the universality of the model. (2) Lack of user behavior data: This study mainly focuses on the relationship between the content characteristics of short videos and the popularity of dissemination, but does not include the audience behavior data (such as click rate, viewing duration and interactivity) for analysis. This may limit the full understanding of the effects of short video transmission.

Funding

Supported by Key Laboratory of Spatial Data Mining & Information Sharing of Ministry of Education, Fuzhou University (No.2023LSDMIS02)

Think Tank for the Construction of Ecological Barrier in the Upper Reaches of the Yangtze and Yellow Rivers in Sichuan Province: Visual analysis of multi-level spatiotemporal data fused with multimodal information (No.202209)

Anhui Cultural Tourism Innovative Development Research Institute: Research on smart tourism innovation based on big data -- building a new scenario of cultural tourism and technology integration (No.ACTK2022YB01)

The funders had no role in study design, data collection and analysis, decision to publish, or preparation of the manuscript.

Conflicts of Interest

The authors declare no conflict of interest.

Data Availability Statement

The collection and analysis method complied with the terms and conditions for the source of the data. All data underlying the findings described in their manuscript to be freely available to other researchers.

References

1. Huang Feng. An analysis of the image characteristics and filming techniques of short video feature reports[J]. New Media Research,2021,7(16):33-35.
2. Qin Feng, Zhou Yujin. Mobile short video content production and optimization strategy based on user comments[J]. China TV,2022(06):92-96.
3. Li M , Mohamed S , Xu H ,et al.DIALOGUE OR MONOLOGUE: A STUDY ON PUBLIC PARTICIPATION IN URBAN IMAGE COMMUNICATION BASED ON SOCIAL MEDIA[J].World conference on media and mass communication, 2023.DOI:10.17501/24246778.2023.7103.
4. Cheng Qianqian. Story, immersion and interaction: the realization path of effective dissemination of short videos of red documents in libraries[J]. Sichuan Library Journal,2022(04):75-79.
5. Huang Xiaoyin,Qiu Zihao. Technology Empowerment and Emotional Interaction:A Study of Visual Music Dissemination on Jieyin Platform[J]. Journal of Southwest University for Nationalities (Humanities and Social Sciences Edition),2019,40(08):156-161.
6. Chen Xintong. Exploring the success of the short food video "Japanese Food"[J]. Modern Communication,2020(10):122-123.
7. Su Qiushi, Liu Caizhi. Research on the application of transition technology in film and television post-production[J]. Computer Knowledge and Technology,2017,13(12):181-184.
8. Wang B .The Role of Data Fusion in Contemporary Urban Image Design and Communication in the Context of Big Data[J].Applied Mathematics and Nonlinear Sciences, 2024, 9(1).DOI:10.2478/amns-2024-2517.
9. Sun Zhenhu,Zhang Yue,Wei Linlu. Transition - the "secret weapon" of smooth video expression [J]. Journalism and Writing, 2020(03):105-108.
10. Yan Hanmei. Analysis of short video production skills[J]. Western Radio and Television, 2022,43(07):69-71.
11. Zhou Y , Wang Z , Zheng S ,et al.Optimization of automated garbage recognition model based on ResNet-50 and weakly supervised CNN for sustainable urban development[J].Alexandria Engineering Journal, 2024, 108:415-427.DOI:10.1016/j.aej.2024.07.066.
12. Chen Xin. Research on short video production innovation in the context of new media [J]. Western Radio and Television,2019(11):15+19.
13. Wang Xiang. The influence of picture language and detail processing on the narrative of TV investigative reporting [J]. News Knowledge,2012(12):79-81.
14. Fan Yuanyuan. Breakfast China: A new journey in the creation of short food documentaries[J]. Media Forum,2020,3(11):10-11+14.
15. Yang Jiesong. The use of camera language in mainstream media short video products[J]. News Front,2022(14):113-115.
16. Xu Jian. Research on the visual communication of short videos on Weibo [D]. Zhengzhou University,2019.
17. Fan Jingting. Analysis of the creative techniques of food documentaries--Taking "Eclipse" as an example[J]. Audiovisual,2019(02):42-43.
18. Yan L , Guofeng H , Miaochao C .Design and Communication of City Brand Image Based on Big Data and Personalized Recommendation System[J].Journal of Function Spaces, 2022, 2022(Pt.9).
19. Shen Yang. Refinement of short video production in the new media environment[J]. News Outpost,2020(01):50-51.
20. Shedid M Y , Hefnawy N H .Historic Preservation as a Tool for City Branding Case Study: Khedivial Cairo[J]. 2022.DOI:10.1007/978-3-030-74482-3_23.
21. Hao Xiaoyuan. Content features and problems of mobile short videos[J]. Communication and Copyright,2017(03):85-87.
22. Shen Qingbin. Analysis of the "simplicity" of NHK documentary images[J]. Audiovisual,2019(12):142-143.
23. Pan Lu. Analysis of the creation of urban documentaries in the short video era[J]. Film Literature,2021(23):45-47.
24. Lin M , Qiming Y .Research on the Application of Short Video in City Image Display[J].International Journal of Science and Engineering Applications, 2023.DOI:10.7753/ijsea1201.1042.
25. Cui Guobin. The selection of originality criteria for live sports event screen [J]. Journal of Soochow University (Law Edition),2019,6(04):1-12.
26. Peng W H, Walls F G, Cohen R A, et al. Overview of screen content video coding: technologies, standards, and beyond[J]. IEEE Journal on Emerging and Selected Topics in Circuits and Systems, 2016, 6(4): 393-408.
27. Wang Zhen. Exploring the practice of shooting current affairs news television screen[J]. News Culture Construction, 2020(06):51-52.
28. Zhang Jieliang, Jiang Weifang, Wang Xin, Zhao Liang. Review of video conference screen quality evaluation methods[A]. China Command and Control Society. Proceedings of the Sixth China Command and Control Conference (Previous Book) [C]. Chinese Command and Control Society:Chinese Command and Control Society,2018:5.

A framework for visualizing and describing city image promotion short video data based on microcube model

Jing He[1,a*], Zhuoluo Yang[2,b] and Jiayi Zhu[1,c]

[1] Institute for Advanced Studies in Humanities and Social Science, Beihang University, Beijing 100183, China
[2] State Key Laboratory of Media Convergence and Communication, Communication University of China, Beijing 100024, China
*Corresponding author: [a]Email: 18610560816@163.com
[b]Email: 287773664@qq.com
[c]Email: bhhejing@buaa.edu.cn

Abstract: Background: The rapid development of media technology and media environment provides rich resources and convenient ways to shape the image of the city. Short video has become an important help to shape the image of the city and build the city brand. How to use short video to shape the image of the city is the key link of urban construction. How: In order to realize comprehensive analysis of short video content, this paper collected a total of 20668 video screenshots for the Douyin platform as data samples, and used Python programming language and Pandas and Matplotlib libraries to process and visualize the data. From the microscopic point of view, a short video data analysis framework of city image promotion based on microcube model is constructed. Objective: To reveal the relationship between video content features and popularity by quantitative and visual methods, and to provide reference for optimizing urban brand promotion strategies. Conclusion: (1) The microcube model shows strong flexibility and applicability in short video content analysis, and can help reveal the complex relationship between short video content characteristics and communication effect. (2) Unexpected events and emotional resonance are key factors in the attractiveness and communication effect of short videos. Reasonable design of scene switching and element interaction significantly enhances the visual impact of short videos.Background: City image carries the heavy responsibility of spreading the city's popularity, reputation and prosperity. The rapidly developing media technology and media environment provide rich resources and convenient ways to build city image, and short videos have now become an important push to shape city image and build city brand. How to use short videos to shape city image is a key link in city construction. Methods: From the microscopic perspective, a short video data analysis framework of city image promotion based on microcube model was constructed. The framework takes dynamic and static characteristics as evaluation indicators to analyze the key elements of city image publicity in short video and the influence mechanism of the communication effect. Objective: To reveal the relationship between video content characteristics and popularity through quantitative and visual methods, so as to provide reference for optimizing urban brand promotion strategies. Conclusion: 20,668 video screenshots from TikTok platform were selected for verification, and it was concluded that the visualization and description framework of city image promotion short video data based on microcube model is feasible and applicable for analyzing city IP image.

Methodology: From a micro perspective, we construct a data analysis framework of short videos for city image promotion based on the micro-cube model. With dynamic features and static features as evaluation indicators, the video contents per unit time encapsulated within each microcube are analyzed, involving operations such as extracting imagery elements of microcubes, transforming geometry and contents of microcubes, combining and parameterizing. Objective: to reveal the relationship between the type of imagery element itself, the arrangement of imagery element position and the appearance time of the imagery element with the hotness of urban short

Formatted: Indent: Left 0.86 ch

video promotion, and to realize the gridded mapping of short video content in the digital world. Conclusion: 20,668 video screenshots from TikTok platform were selected for verification, and it was concluded that the visualization and description framework of city image promotion short video data based on microcube model is feasible and applicable for analyzing city IP image.

Keywords: City Image Promotion;, Short Video;, Micro Perspective;, Imagery Elements

1 Introduction

In the world, cCity image publicitypromotion has become an important issue in city management and economic development. Especially in today's rapid development of digital media, short video stands out among many communication methods in the field of new media, combining the "mirror" of the city in the form of fragmentation, uniqueness and fluidity, and rapidly spawning a variety of new Internet celebrity cities, which has great influence in attracting potential tourists, and also provides an important platform for the export of urban culture and international brand image. However, the performance of short video propaganda is obviously different in different cities, and there is a lack of systematic methods to evaluate the specific impact of video content on the visibility and communication effect of cities. Therefore, how to analyze short video content from the perspective of data and visualization and provide optimization suggestions for urban publicity strategies has become an important issue that needs to be explored. Most of the existing researches focus on the communication characteristics or creative skills of short video, and lack of quantitative content analysis to analyze the specific role of short video in shaping city image. For example, how to analyze short video content more scientifically and extract the key factors affecting the dissemination effect? Starting from microscopic content analysismicro perspective, this study proposes a short video analysis framework to explore the correlation between short video content features and communication effects. This analysis framework can not only provide ideas for the brand communication of Chinese cities, but also provide reference for the optimization practice of urban image publicity under the background of globalization.Short videos stand out among the many communication methods in the new media field, with the characteristics of short, new and fast, putting together the "mirror image" of cities in a fragmented, unique and flowing form, and rapidly giving rise to various new types of net celebrity cities. Along with the short videos, which have created a host of net celebrity cities and hot spots, the interactive participation of mainstream media and citizens in the image and discourse of the city has led to a new way of shaping the city's image. Take Beijing as an example, how to land the short video of city image promotion in this capital city, which has a model role as a national cultural center for building a socialist cultural power, is not only important for Beijing's urban management and planning, cultural perception and tourism resources development, but also has a reference value and demonstration significance for the image shaping and promotion of other Chinese cities in adapting to the mediated society, and also provides an opportunity for China's and even the world's It also provides a Chinese solution for China's and even the world's urban culture construction that combines cultural heritage and contemporary development. Therefore, the optimization of short video communication of city image propaganda is an important part of city image construction that needs to be deepened and effectively developed.

Formatted: Font: (Default) Palatino Linotype, 10 pt

2 Related work

At present, domestic and foreign scholars mainly focus on the content production, communication characteristics, development status and ethical dilemmas of short videos,

and relatively little research is conducted based on the screen perspective of short videos. After reviewing and summarizing the existing related literature, it is found that short video screen research mainly focuses on six types.

(1) Unexpected events

The advent of the mobile Internet era means the advent of the era of attention economy, where short videos with novel content can get the audience's attention first and content that exceeds the audience's expectation can rapidly seize the market share~~ in advance~~. Unexpected events refer to unpredictable and unrepeatable scenes that are presented in short videos, becoming key highlights to attract more audience attention. Huang Feng (2021) suggests that the unexpected events in life are irreproducible and will be presented in the short video screen to become a great highlight and gain more attention from the ~~audience ;~~audience~~;~~. Qin Feng et al. (2022) found that differentiated video material, ups and downs, and innovative elements would reduce viewers' boredom by analyzing short video user comments. M Li et al. (2023) pointed out that content involving unexpected scenes (such as emergencies or unexpected turns) in videos has a much higher forwarding volume than that of ordinary videos. This "surprise effect" suggests that unexpected events play a significant role in attracting attention.

(2) Emotional resonance

Unlike traditional media, where "content is king", new media focus more on "people-oriented", attaching importance to people's emotional expression and emphasizing immersion experience. Emotional resonance involves evoking emotional connections with audience through video content, thus enhancing engagement and loyalty. Cheng Qianqian (2022) proposes that adding regional cultural elements of revolutionary base areas such as dialects in short videos of red documents can create a familiar cultural atmosphere for viewers, reinforce the sense of regional belonging while restoring history, and enhance viewers' immersion and sense of immersion~~;~~. Huang Xiaoyin (2019) argues that emotion is currently recognized as the basis for sustaining users and is the strongest link between users and the platform, whether it can inspire users' emotional resonance has also become an important indicator to judge whether media products have influence. As the head IP of food short videos, "Eclipse" , uses the cooking process of food to convey the attitude of "living well" and inspires users to identify with and "be in" the emotional level by constructing a specific daily life space. This will lead to the emotional resonance of users. B Wang (2024) found that short videos can significantly increase users' willingness to share by enhancing positive emotional expression, especially in content involving cultural identity and community belonging.

(3) Scene transition

The smallest unit of the video is the shot, and scene transition refers to~~the~~ techniques that enable smooth transitions between consecutive shots to ensure continuity of time and space~~to make the front and back two shots switch smoothly is the transition. In addition to grouping shots, transitions have the function of serving the video content.~~ . According to Sun Zhenhu (2020), t~~he purpose of transitions is to shift time and space, so that the scenes are separated while maintaining a coherent and smooth transition of time and space. T~~he reasonable use of transitions can make the content presentation rational and the story elaboration clear, so that the audience can easily understand and accept it~~;~~. Yan Hanmei (2022) introduces three types of techniques to achieve transitions from the perspective of screen editing: fluent editing, matching editing and jump cutting, and selects appropriate editing techniques to meet the best viewing experience of viewers according to different short video production needs. The reasonable use of transitions can also make the news report free from traditional forms. For example, the short video of "One Day Patrol of Civil River Chief" presents the whole "river chief system" activities in a minute with multiple scenes, transitions and angles. Y Zhou (2024) proposed that rapid scene switching can enhance the rhythm of the video, but if the switching is too frequent, it may reduce the viewing comfort of the audience.

(4) Elemental amplification

Elemental amplification involves focusing on details to highlight the characteristics of characters and events. The essence of details is "amplification", by capturing details, the characteristics of characters and events can be highlighted. After studying Breakfast China, Fan Yuanyuan found that the short documentary left behind large scenes, used more close ups and close-ups to show people and actions, paid attention to detail control, and presented breakfast such as steaming Fujian Saiki fried buns and fragrant Guangdong Chaoshan pig's blood soup on the small screen to truly show people's living conditions. The short video "Tribute to the People's Police" produced by Quanzhou Radio and Television Station in conjunction with Quanzhou Public Security Bureau contains close-up images of public security police using DNA technology for data comparison and analysis and research, highlighting the professionalism of the people's police and how technology can help criminal investigation work. Y Lin (2022) analyzed close-ups in short videos and found that enlarging detailed elements (such as food texture and architectural decoration) can effectively attract viewers' visual attention and enhance content memory.

(5) Element interaction

In the traditional media period, the picture elements are flat and single, newspaper pictures are a combination of colors, and TV reports use the normalized camera language and transitions to complete the narrative. In contrast, in the new media era, element interaction short videos are paired withrefers to the use of various visual elements that do not appear in isolation as a single symbol to increase the visual impact in a limited screen. For example, the characters in "The Day of Food" include a Persian cat endowed with a distinct personality in addition to the chef Jiang Laodao, who is the introducer at the beginning of the video, the spectator of the cooking process, and sometimes the taster of the final result. The short video does not set up human emotions, but expresses emotions from the cat's point of view, making the video easy and interesting, but real and natural. Shen Yang (2020) argues that in short video refinement production, the small depth-of-field images captured by the combination of large aperture and telephoto can obtain a sharper perspective and reflect the image texture and ingenuity as it is. MY Shedid (2022) found that in short videos, dynamic interactive scenes (such as the dialogue between natural elements and modern buildings) can enhance the audience's immersion through the integration of multi-modal information.

(6) Screen style

Screen style refers to the overall visual tone and atmosphere of short video, encompassing color grading, lighting, and cinematographic techniques. Different styles can convey specific moods and influence audience perception. Hao Xiaoyuan (2017) took One, Ershang and Pear Video as the research objects and found that the styles of One and Ershang are both inclined to fresh and literary style, and Pear Video presents a variety of styles due to its worldwide patron system;. Shen Qingbin (2019) found that most of NHK's documentaries use plain means to pick up images and focus on the communication of live atmosphere. Compared with the BBC, where every shot is perfect, NHK's documentary images are more plain and simple, and low-key and subtle, so he believes that NHK's documentary images as a whole present a rustic style. In the urban documentary Jin Zhi Yang, subdued tones are used to adjust the relationship between light and shadow to outline the scene of the dignified and mysterious urban architectural complex of Taiyuan, Shanxi Province, highlighting the unique historical and humanistic atmosphere of Taiyuan. L Ma (2023) analyzed the picture styles in urban short videos and found that highly consistent and aesthetically rich visual styles are more likely to establish audience's emotional attachment to video content. For example, fresh-styled videos are significantly more appealing to younger users than other genres.

In addition to the above six parts, scholars at home and abroad have also studied other aspects of picture content, such as multimedia technology, legal copyright, post-production, shooting composition, etc., but literature data are even scarcer. For

example, Cui Guobin (2019) explored whether live sports event screens have originality in the sense of film works;. Peng (2016) and others introduced the latest progress in video coding of screen content;. Wang Zhen (2020), on the other hand, analyzed the principles that should be followed in filming current affairs news TV screens and discussed the filming techniques of different types of TV current affairs news;. Zhang Jieliang et al. (2018) explored the method of video conference screen quality evaluation from both subjective and objective aspects.

In summary, this paper obtains city image short video data with geographical location markers, takes the research literature of domestic and foreign research scholars on short video images as a reference basis, combines the presentation patterns of city image propaganda on short video, including content themes, emotional bias, media sources, communication characteristics and dynamic assessment of overseas media, traditional mainstream media communication discourse insight, official media and local self-media synergistic development The content production characteristics of short video images are extracted and initially set as six imagery elements: unexpected events, emotional resonance, scene transformation, element amplification, element interaction, and picture style, as the research dimensions of the data visualization description framework of city image propaganda short video based on the microcube model.

3 Microcube model framework construction

3.1 Unexpected events - Sense of novelty

Taking the micro perspective as the analysis perspective, the video content is refined and a content microcube model is established to encapsulate the complete short video into multiple fragmented short videos according to the time interval, i.e., each small cube encapsulates the video content per unit of time, as shown in Figure 1(a).

Then an assignment operation is performed on each small cube. This operation contains multiple inputs a1, a2 ..... .an (technology elements, food elements, natural landscape elements, cityscape elements, people elements, animal elements, and other imagery elements), an output z, and multiple intermediate parameters (the number of parameters is comparable to the number of time intervals). Among them, a1, a2 ..... .an are set to exist equal to 1 and not exist equal to 0. The intermediate parameter values take values that vary in multiples. A directional arrow indicating the connection can be understood as follows: at the input side, the size of the transmitted signal is a, and there is a change parameter w in the middle of the end, and the signal becomes a*w after several changes.

Let the total duration of the short video be t. After n times of segmentation, the signal size at the output end becomes $a*\left(\frac{w_1}{n}+\frac{w_2}{n}+\cdots+\frac{w_n}{n}\right)$, i.e. $a*\frac{w}{n}$. If the range of $\left|\frac{a*w}{n}-a\right|$ satisfies the threshold, the video content display is considered smooth;. if the range exceeds the threshold, the video content display is considered transitive and contains the transitive element T. The event that triggers this transitive is called an unexpected event. Figure 1(b) shows the schematic diagram of the occurrence of unexpected events in short videos. 3-4s and 6-7s are both transitions time domain, and the transitions region is visualized by color, and its shades represent the degree of transitions.

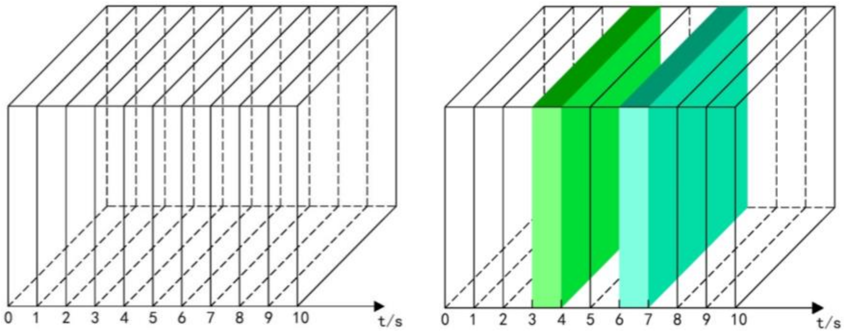

(a) (b)

Figure 1 Refinement analysis of the content presentation module

(a) content cut~~;~~. (b) accidental event microcube visualization model

Formatted: Indent: Left: 0"

3.2 Emotional resonance - Sense of immersion

Short videos are in a sense emotional product~~s~~ in the process of communication. Experimentally, we designed an emotion grid chart with nine emotions including moved, praised, funny, happy, neutral, angry, sad, shocked and helpless, and used #EE6363, #EEDFCC, #FFA54F, #FFEC8B, #FFFFF0, #00F5FF, #63B8FF, #00FF7F and #CD69C9 as RGB color controls, respectively. as shown in Figure 2(a).

Then, the emotion grid map is color-matched with the content cube model (neutral to white, not identified as color~~ color~~ system, and its fractional video number is set to N), and the result of matching is that the emotion marker is implemented to the content cube model, and the marker color is consistent with the RGB color type in the emotion grid map. Then the complete short video will be divided into multiple color-based fractional short videos A according to the time interval, i.e., each small cube encapsulates the emotional content per unit time. Among them, the color matching degree $M = \frac{A-N}{A}$, when M>30%, is strong in emotional substitution. Figure 2(b) is a schematic diagram of color matching degree in the city image video with M=50%, 2-~~s 3s, 3~~ 4s, ~~4-5~~7-8s, ~~7-8~~8-9s, and three emotions of happy, shocked, and helpless, respectively.

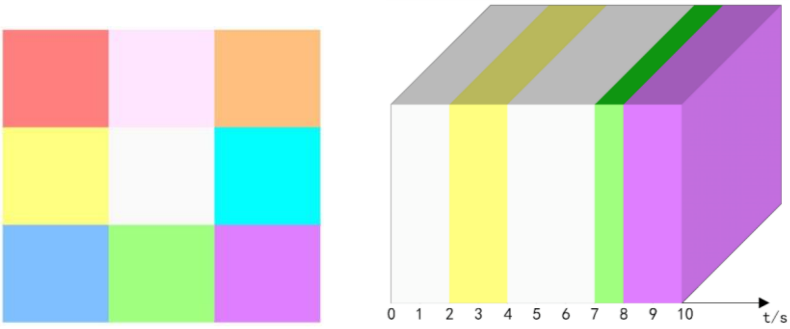

(a) (b)

Figure 2 Refinement analysis of emotional carryover

(a) emotional grid diagram;, (b) color matching results

3.3 Scene transition - Sense of Following

Scene transitions imply the existence of a relatively large change in temporal or spatial relationships. Time is divided into broadcast time, event time, and narrative time;, space is divided into physical space and psychological space;, physical space is the display space of actual transitions, and psychological space is the space adjusted with the ups and downs of the storyline. The TS level transition scenes include T1, T2, T3, S1, S2 and Tn/Sn. Among them, T1, T2, and T3 represent the corresponding broadcast time conversion, event time conversion, and narrative time conversion;, S1 and S2 represent the corresponding physical space conversion and mental space conversion;, and Tn/Sn represents the hybrid conversion of time and space connection. The TS level conversion domain model describes the abstract static structure of the system, and only after matching the dynamic behavior can the complete representation of the system be completed.

In the TS level conversion scenario, two typical metrics, switching correctness and real-time, are selected. Switching correctness requires the following 2 conditions to be discerned: first, based on the completion of the short video to be able to enter successfully different scene states;, second, traversing the frame state paths of all standard samples in the short video. Real-time metrics require not only the complete short video playback to respond within a determined time, but also to ensure the correctness of the response. In this scenario, once the relevant time constraint is violated, it will not only affect the efficiency of scene transition, but may also cause the coherence degree of video playback.

Analysis of scene conversion for the video after content refinement: let the total propagation time of the short video be t. After n times of segmentation, visualize each scene as a different color cube within the unit time, and the color switching represents the conversion of the scene, and let the number of scene switching be q. In Fig. 3(a) ~ Fig. 3(d), q is equal to 3, 2, 0, and 6, respectively.

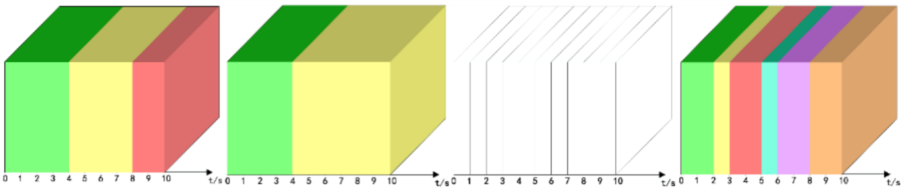

(a) (b) (c) (d)

Figure 3 Scene transition microcube visualization model (a)scene transitioned three times;, (b) scene transitioned twice;, (c) no scene transition;, (d) scene transitioned six times

Formatted: MDPI_5.1_figure_caption

3.4 Amplification of elements - Sense of in-depth

In fact, for the short video platform, even the city image promotion is shown as "content production", which usually contains one or more imagery elements such as technology elements, food elements, natural landscape elements, urban landscape elements, people elements, animal elements, etc. We designed the imagery elements grid and used #00FF7F, #FFEC8B, #EE6363, #00F5FF, #EE82EE, #FFA54F as RGB color controls, as shown in Figure 4(a).

The video content is further refined to establish the tiny content cube model, then each fragmented short video is further divided into 100 sub-cubes equally, i.e., each

sub-cube encapsulates the video content per unit time and per unit screen, as shown in Figure 4(b). Let each sub-cube be 1 unit, when cube cutting is performed on the short video sample data, one or more of the imagery elements may appear on the video screen per unit of time. The percentage of occupied screens for each imagery element is quantitatively analyzed and visualized with different colors. Elements with screen occupancy greater than 30% and scene time greater than 3s are extracted and identified as amplified elements, set as E. Amplification of elements is relative to each other and there is no gradual change over time or other measure. Representation = f(e1, e2, . . , e3), where e is the elements that characterize the short video content. Figure 4(c) shows four amplification elements, where 0-4s are cityscape elements, 6-8s and 10-11s are food elements, and 8-9s and 13-14s are technology elements.

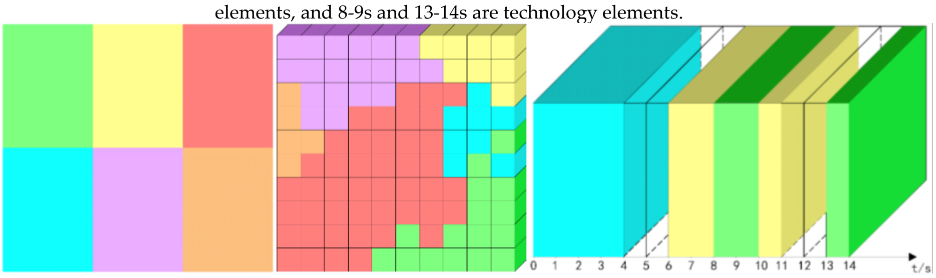

(a) (b) (c)

Figure 4 Refinement analysis of element amplification (a) color grid diagram of imagery elements;, (b) sub-cube model;, (c) this sample data contains 4 amplified elements

Formatted: MDPI_5.1_figure_caption

3.5 Element interaction - Sense of dynamism

The analysis of the amplified elements continues with further decomposition of elements of the same category. The decomposition is based on the principle of interaction, i.e., when there is interaction between elements of the same category, only then the element continues to be decomposed into multi-category interactive elements, and the original element is represented by OE and the decomposed element is represented by DE. The visualization color of DE is determined by the visualization color of OE as a sub-color family of OE. Let the total propagation time of the short video be t. After n times of segmentation, the microcubes with different color particles are visualized according to the element categories in each subcube per unit time, among which, the original element of technology OE technology is original green, the original element of food OE food is original yellow, the original element of natural landscape OE natural landscape is original red, the original element of urban landscape OE city is original cyan, and the character The original element OE characters is the original purple, and the original element OE animals is the original orange. When the imagery element is judged to be an interactive element, OE technology is decomposed into OE technology 1 + OE technology 2, OE cuisine is decomposed into OE cuisine 1 + OE cuisine 2, OE natural landscape is decomposed into OE natural landscape 1 + OE natural landscape 2, OE cityscape is decomposed into OE cityscape 1 + OE cityscape 2, OE character is decomposed into OE character 1 + OE character 2, and OE animal is decomposed into OE animal1+OE animal2, as shown in Figure 5(a).

Let the total propagation time of the short video be t. After n times of segmentation, the different sub-cubes per unit time are decomposed into multiple micro-cubes with different colors, as shown in Figure 5(b). Firstly, the amplified elements are judged according to the method described in 4.4, and then the decomposition analysis of the

elements is performed on the amplified elements, and the color decomposition is recorded, and Figure 5(c) demonstrates the decomposition rate d=70%.

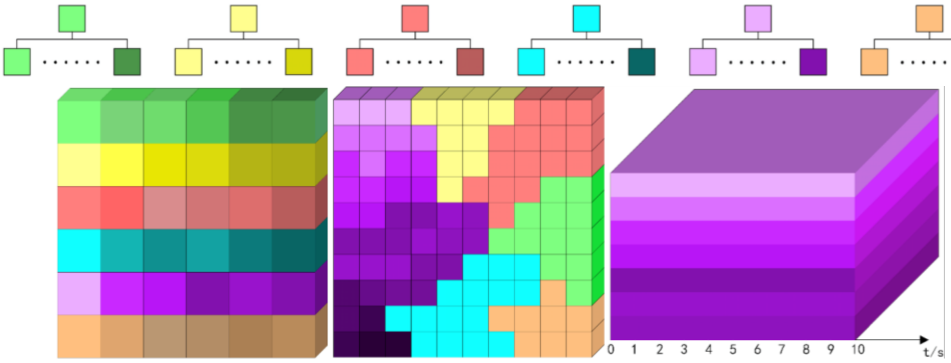

(a) (b) (c)

Figure 5 Refinement analysis of element interactions (a) color grid diagram of the decomposition of imagery elements;, (b) microcube model of character element interactions;, (c) decomposition of 7 interactions of character elements

Formatted: MDPI_5.1_figure_caption

3.6 Screen style - Main color

Each image in the video can be abstracted into three forms: point, line and surface, and the effective presentation of these forms are the elements. The above decomposition of different elements has been carried out, and the decomposed elements are the basic framework to form the microcube model. However, the video screen is often more than a simple combination of individual elements, and sometimes involves an effective merging of elements to form the style of the overall video screen. A grid diagram of the main colors of the screen style was designed, and #0000FF, #BEBEBE, #FFFACD, #6495ED, and #FFC0CB were used as RGB color controls for dark, gray, light, medium, and bright tones, respectively, as shown in Figure 6(a). Figure 6(b) shows five main colors of the screen, among which, 0-2s and 4-5s are bright tones, 2-4s are gray tones, 5-7s are dark tones, 7-8s are light tones, and 8-10s are medium tones.

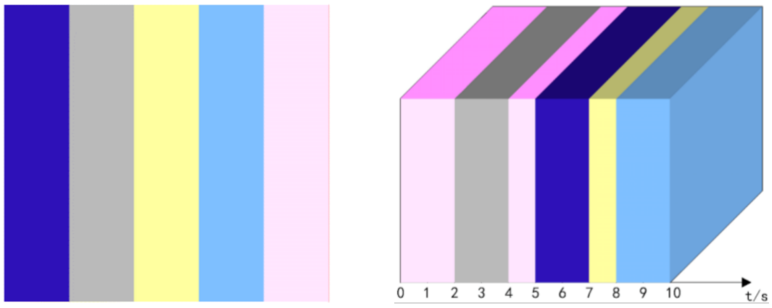

(a) (b)

Figure 6 Refinement analysis of picture styles (a)color grid diagram of primary colors;, (b) visual framework of 5 primary colors

Formatted: Indent: Left: 0"

4 Analysis of cases

There are three levels in the short video communication effect rating index model: the criterion level, the sub-criterion level and the solution level. The criterion layer is based on the hotness, breadth and depth of communication, the sub-criteria layer sets the second-level indicators based on the media hotness, fan hotness, search hotness, reputation of each network platform, content acceptance, cross-domain linkage, crowd reach, thematic distinctness and thematic diversity, the solution layer corresponds to the sub-criteria layer and refines the relevant indicators. After applying the index model to evaluate the communication effect of the short videos of Beijing city image propaganda, the absolute hotness (H) concept is used to calculate the hotness value of the short videos of online celebrities using a one-year time period as the time period of attention: H=(xCa+yCb+zCc)(1+Cd). Where H denotes the absolute hotness value, Ca denotes the value calculated by the propagation hotness criterion layer, Cb denotes the value calculated by the propagation breadth criterion layer, Cc denotes the value calculated by the propagation breadth criterion layer, Cd denotes the account quality of the video publisher (depending on the account quality of the video publisher, the value is between 0 and 1), x and y denote the factor weights (depending on the importance of the factor, the value is between 0 and 1). . Accordingly, the city image promotion short videos in different heat ranges are categorized. Appendix I shows the design model of short video hotness index system.

Based on the hotness value of the short videos of Netflix, this experiment selected a total of 1200 short videos of popular city image propaganda short videos from the TikTok platform with likes >1w and retweets >0.8w and non-popular city image propaganda short videos with likes <1w and retweets <0.8w within the second half of 2019, and a total of 20,668 video screenshots were obtained after the screen draw seconds per second, and ensure that the video content includes geolocation tags and a clear city promotion theme. To ensure the authenticity and representativeness of the video content, the research team manually reviewed all selected videos and cross-verified them with the metadata provided by the platform, such as upload date, tag and post account number, to eliminate possible noise data (In the obtained videos, all content is strictly in accordance with the data use policy, only for academic research, and does not involve personal privacy information). Table 1 shows the number of microcubes and the number of video screenshots per second in the framework of visualization description of city image promotion short video data based on microcubes model for popular cities and non-popular cities. Further, plot the relationship heat matrix for popular and non-popular cities in the following dimensions: the number of unexpected microcubes, number of unexpected frames, number of emotion resonance microcubes, number of positive emotion microcubes, number of negative emotion microcubes, number of emotion resonance frames, number of positive emotion frames, number of negative emotion frames, number of scene transition microcubes, number of scene transition frames, number of elemental amplification microcubes, number of elemental amplification frames, number of microcubes with at least two amplification elements, number of frames with at least two amplification elements, number of elemental interaction microcubes, number of elemental interaction frames, number of gray tone microcubes, number of gray tone frames, number of dark tone microcubes, number of dark tone frames, number of bright-toned microcubes, number of bright-toned frames, number of mid-toned microcubes, number of mid-toned frames, number of light-toned microcubes, and number of light-toned frames. The relationship heat matrices are plotted in terms of the number of frames, the number of bright-tone microcube models, the number of bright-tone pumping frames, the number of mid-tone microcube models, the number of mid-tone pumping frames, the number of light-tone microcube models, and the number of light-tone pumping frames, as shown in Figure 7, each using the same

emerald green color space, but using a different color mapping. The greener the color represents the stronger the correlation between the two factors, and is dark green when both are the same element (as shown in the diagonal of the matrix), indicating a 100% correlation. The redder the color means the less correlation between the two factors. Comparing the two heat matrix plots, it is found that there are significantly more green color blocks in popular cities (a) than in non-popular cities (b), and the green color concentration is greater;, while there are significantly more red color blocks in non-popular cities (b) than in popular cities (a), "more than the number of microcube models of both elements" The number of "microcubes with more than two elements" is negatively correlated with nearly half of the other elements, and the number of brightly colored second frames is significantly negatively correlated with the number of brightly colored second frames. The above analysis shows that there is a correlation between the intention elements of short videos from popular cities, i.e., there may be multiple intention elements in the same picture, while the intention elements of short videos from non-popular cities have a weaker relationship with each other, and the intention elements in the video picture are relatively single.

Formatted: Font: (Default) Palatino Linotype

Formatted: MDPI_3.1_text

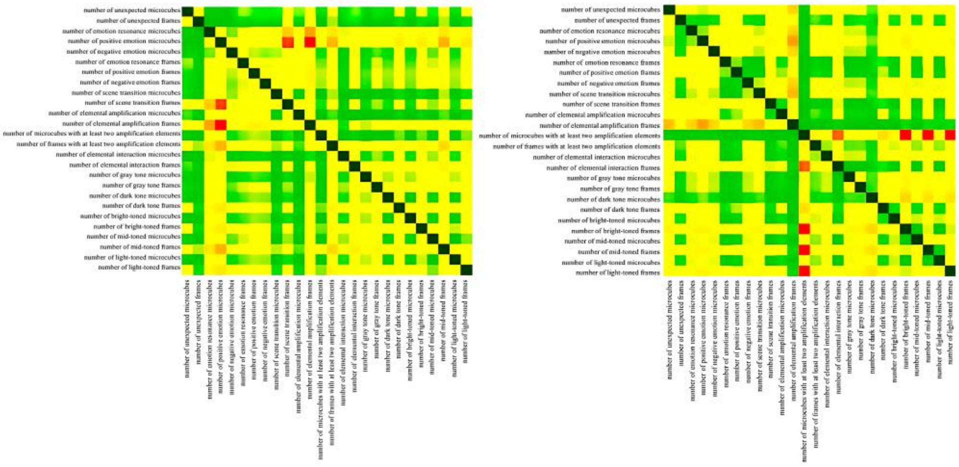

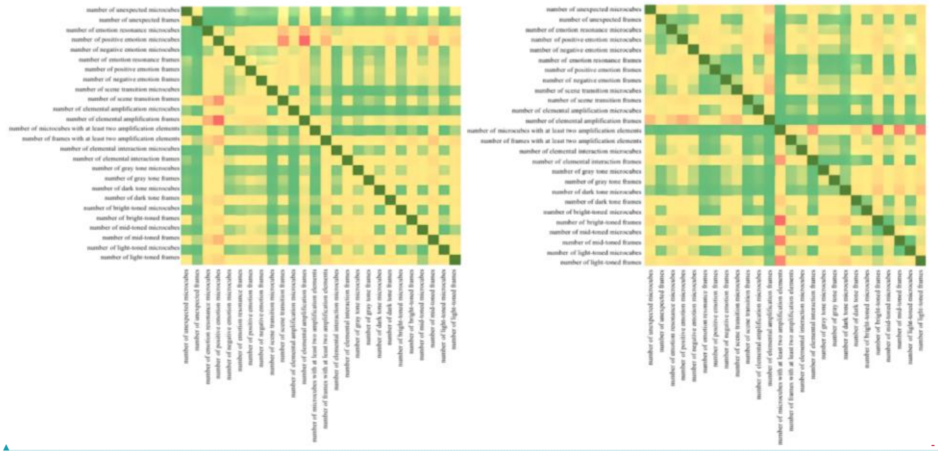

(a) (b)

Figure 7 Relationship heat matrix diagram of short video screen visualization relationship heat matrix display of sample data for popular cities;. (b) relationship heat matrix display of sample data for non-popular cities

Due to space limitation, only 144 city popular short video microcubes models with 2393 city popular short video screenshots and 123 city non-popular short video microcubes models with 1834 city non-popular short video screenshots are shown here. To clearly demonstrate that the microcube model-based descriptive framework for visualizing city image promotion short video data can be used to perform descriptive analysis of content abstraction of popular and non-popular city short videos occurring in a time series, a stack of microcube models is used for presentation.

Table 1 Overview of the microcube model constructed for popular/non-popular cities

popular cities	Beijing	Chengdu	Guangzhou	Nanjing	Nanning	Shanghai	Shenzhen	Wuhan	Xian	Zhangjiajie	Changsha	Chongqing	Total
Number of microcube models	12	12	11	13	13	10	13	12	13	11	11	13	144
Number of video frames in seconds	216	205	205	232	216	149	220	186	210	154	182	218	2393

non-popular cities	Alashanmeng	Baoding	Beijing	Chengdu	Foshan	Guangzhou	Hangzhou	Shanghai	Shenzhen	Xian	Zhengzhou	Chongqing	Total
Number of microcube models	13	13	9	7	11	10	11	10	10	9	10	10	123

Formatted: Font: (Default) Times New Roman Regular

Formatted: MDPI_5.1_figure_caption

Number of video frames in seconds	211	182	136	107	157	140	164	154	170	135	131	147	1834

4.1 Visualization results of unexpected events of short videos based on microcube model

Based on the display data, the number of microcube models containing unexpected events and the number of video pumping second frames were recorded as shown in the following table.

Table 2 Number of microcube models containing unexpected events and number of video pumping seconds frames

	Popular city image short video	Non-popular city image short video
Number of microcube models containing element T	106	39
Number of video frames in seconds	408	123

The number of microcube models containing element T in the popular city image videos is 106, and the number of video second draws is 408, while the number of microcube models containing element T in the non-popular city image videos is 39, and the number of video second draws is 123.

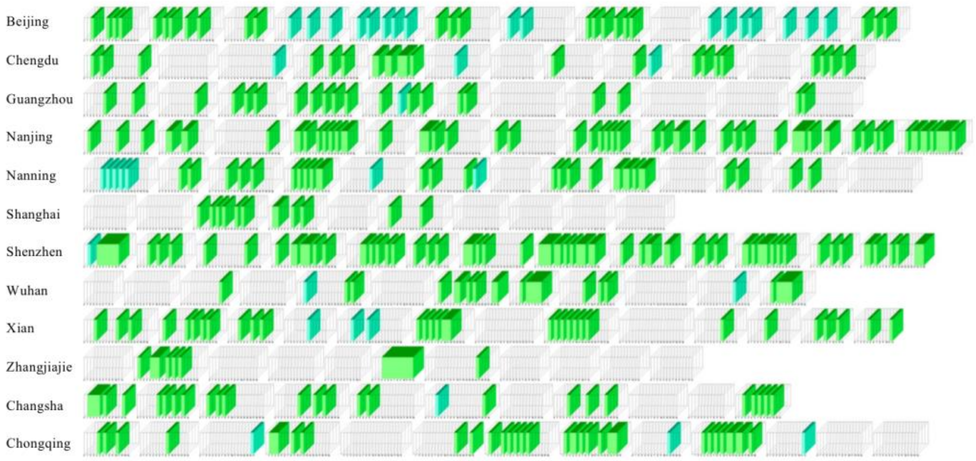

(a)

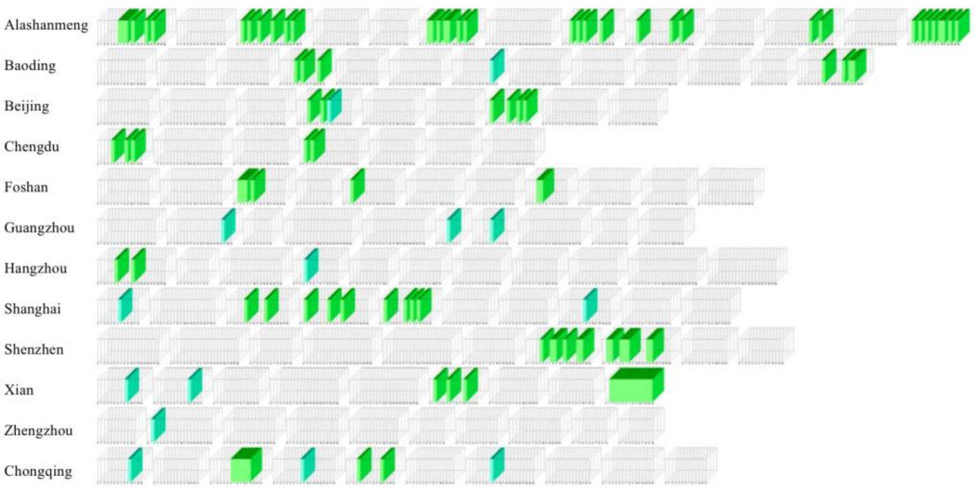

(b)

Figure 8 Accidental event stacking graph based on microcube model sample case of popular city image short video;. (b) sample case of non-popular city image short video

Formatted: MDPI_5.1_figure_caption

4.2 Visualization results of emotional resonance of short videos based on microcube model

Based on the display data, the number of microcube models containing the nine emotions and the number of video pumping second frames were recorded as shown in the table below.

Table 3 The number of microcube models containing emotional elements and the video pumping second frames

	Popular city image short video	Non-popular city image short video
Number of microcube models containing emotional elements	69	35
Color matching of total model M1	47.92%	28.46%
Color matching of total emotion M2	9.21%	5.41%
Number of microcube models involving positive emotions	47	32
Color matching of total model M1	32.64%	26.02%
Color matching of total emotion M2	61.84%	91.43%
Number of microcubes models involving negative emotions	29	5
Color matching of total model M1	20.14%	4.07%
Color matching of total emotion M2	38.16%	13.51%
Number of microcubes models involving both positive and negative emotions	7	2
Color matching of total model M1	4.86%	1.63
Color matching of total emotion M2	9.21%	5.41%

The number of microcubes containing emotional elements in the urban popular short videos was 69, and the color matching degree of the total model M1=47.92%. Among them, the number of microcubes involving positive emotions is 47, the color matching degree of the total model M1=32.64%, the color matching degree of the total emotion M2=61.84%;, the number of microcubes involving negative emotions is 29, the color matching degree of the total model M1=20.14%, the color matching degree of the total emotion M2=38.16%;, the number of microcubes involving both positive and negative emotions is 7. The number of microcubes involving both positive and negative emotions is 7, the color matching degree of total models M1=4.86%, and the color matching degree of total emotions M2=9.21%.

The number of microcubes containing emotional elements in the non-popular city image videos is 35, and the color matching degree of the total model is M=28.46%. Among them, the number of microcubes involving positive emotions is 32, the color matching degree of total models M1=26.02%, the color matching degree of total emotions M2=91.43%;, the number of microcubes involving negative emotions is 5, the color matching degree of total models M1=4.07%, the color matching degree of total emotions M2=13.51%;, the number of microcubes involving both positive and negative emotions The number of microcubes involving both positive and negative emotions was 2, the color matching degree of the total model was M1=1.63%, and the color matching degree of the total emotion was M2=5.41%.

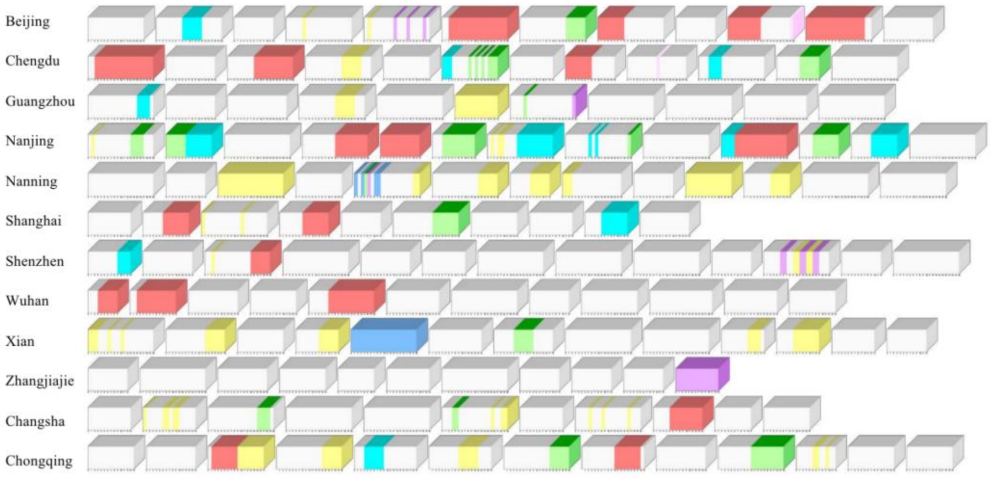

(a)

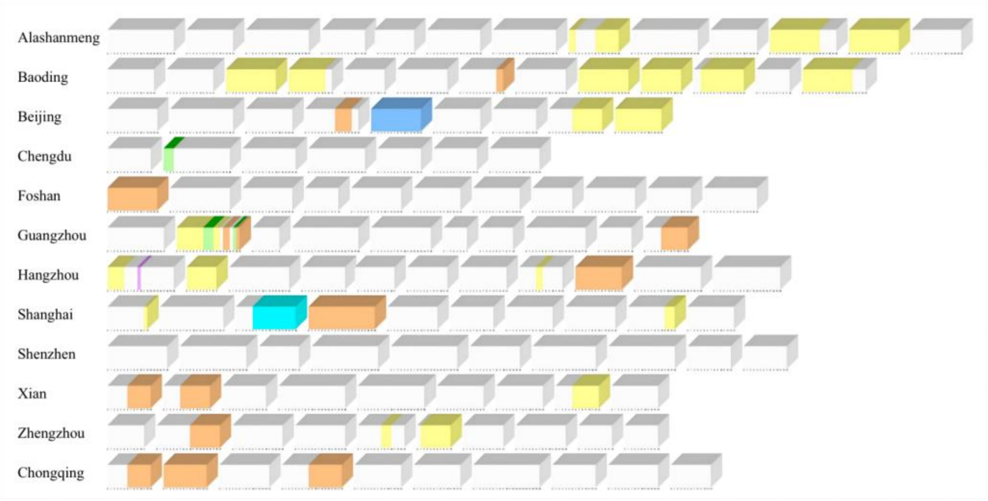

(b)

Figure 9 Emotional resonance stacking diagram based on microcube model (a) sample case of popular city image short video;, (b) sample case of non-popular city image short video

4.3 Visualization results of scene transition of short videos based on microcube model

Based on the display data, the number of microcube models containing scene transitions and the number of video pumping seconds frames were recorded as shown in the table below.

Table 4 Number and percentage of microcube models containing scene transformation

	Popular city image short video	Non-popular city image short video
Number/proportion of microcube models containing element q	91/67.36%	26/21.14%
Number of microcube models with q=2/percentage	14/10.37%	3/2.44%
Number of microcube models with q=3/percentage	18/13.33%	7/5.69%
Number of microcube models with q=4/percentage	26/19.26%	6/4.88%
Number of microcube models with q=5/percentage	13/9.63%	5/4.07%
Number of microcube models with q=6/percentage	8/5.93%	1/0.81%
Number of microcube models with q=7/percentage	8/5.93%	2/1.63%
Number of microcube models with q=8/percentage	3/2.22%	0/0%

The number of microcube models containing element q in popular city image videos is 91, accounting for 67.36%;, among them, the number of microcube models with q=2 is 14;, the number of microcube models with q=3 is 18;, the number of microcube models with q=4 is 26;, the number of microcube models with q=5 is 13;, the number of microcube models with q=6 is 8;, the number of microcube models with q=7 is The number of microcubes is 8;, the number of microcubes with q=8 is 3.

The number of microcube models containing element q in the non-popular city image videos is 26, accounting for 21.14%;, among them, the number of microcube

Formatted: Indent: Left: 0"

Formatted: Font: 10 pt, Italic

models with q=2 is 3;, the number of microcube models with q=3 is 7;, the number of microcube models with q=4 is 6;, the number of microcube models with q=5 is 5;, the number of microcube models with q=6 is 1;, the number of microcube models with q=7 is 2. The number of models is 2.

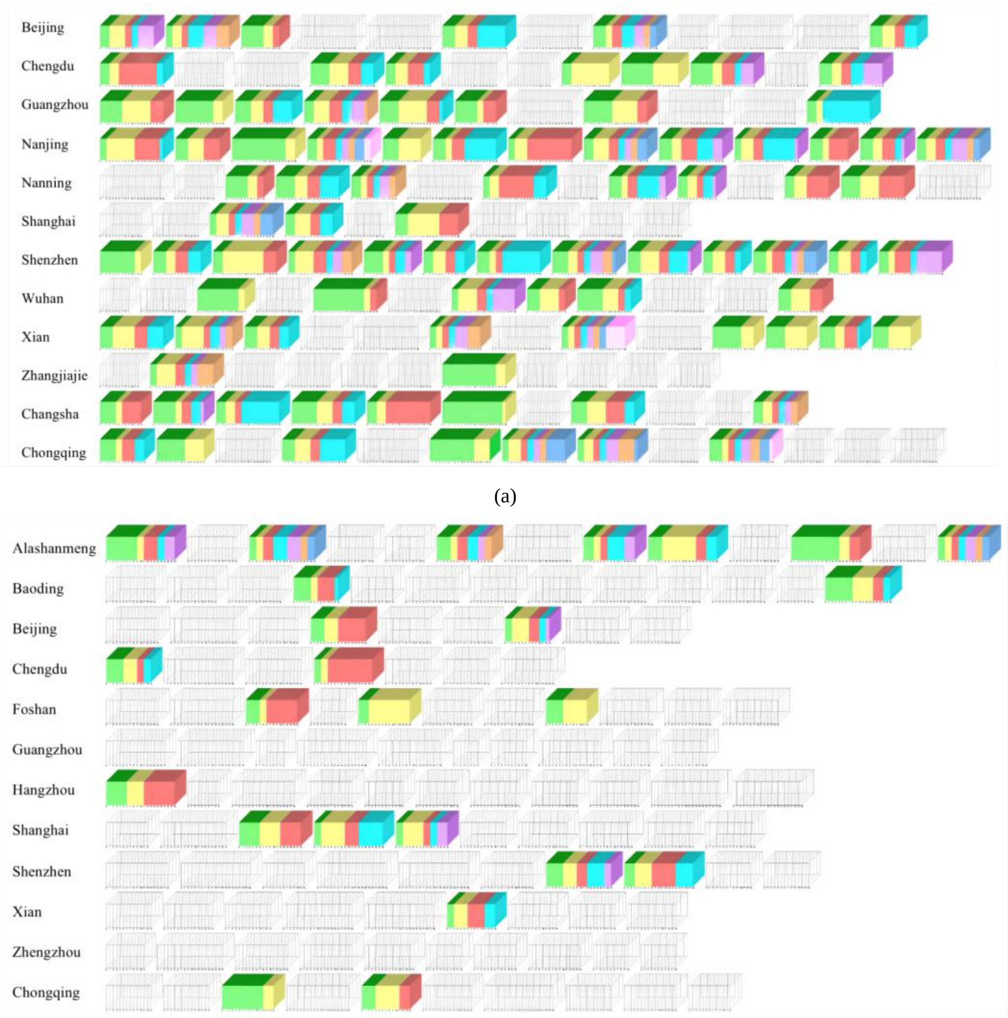

Figure 10 Scene transformation stacking diagram based on microcube mode sample case of popular city image short video;, (b) sample case of non-popular city image short video

Formatted: MDPI_5.1_figure_caption

4.4 Visualization results of amplification of elements based on microcube model

Based on the display data, the number of microcube models containing the six types of imagery elements and the number of video pumping seconds frames were recorded as shown in the table below.

Table 5 Number of microcube models containing intentional elements

	Popular city image short video	Non-popular city image short video
Number of microcubes containing imagery elements	141	116
Number of microcubes with one type of imagery	93	104
Number of microcubes with two or more types of imagery	48	12
Number of microcubes with technological elements	3	0
Number of microcubes with food elements	33	0
Number of microcubes with natural landscape elements	21	6
Number of microcubes with urban landscape elements	74	25
Number of microcubes with human elements	66	89
Number of microcubes with animal elements	4	0

The number of microcubes containing imagery elements in popular city image videos is 141, the number of microcubes containing one type of imagery elements is 93, and the number of microcubes containing two or more types of imagery elements is 48. Among them, the number of microcubes involving technology elements is 3, the number of microcubes involving food elements is 33, the number of microcubes involving natural landscape elements is 21, the number of microcubes involving urban landscape elements is 74, the number of microcubes involving people elements is 66, and the number of microcubes involving animal elements is 4.

The number of microcubes containing imagery elements in the non-popular city image videos is 116, the number of microcubes containing one type of imagery elements is 104, and the number of microcubes containing two or more types of imagery elements is 12. Among them, the number of microcubes for urban landscape elements is 25, the number of microcubes for people elements is 89, and the number of microcubes for natural landscape elements is 6.

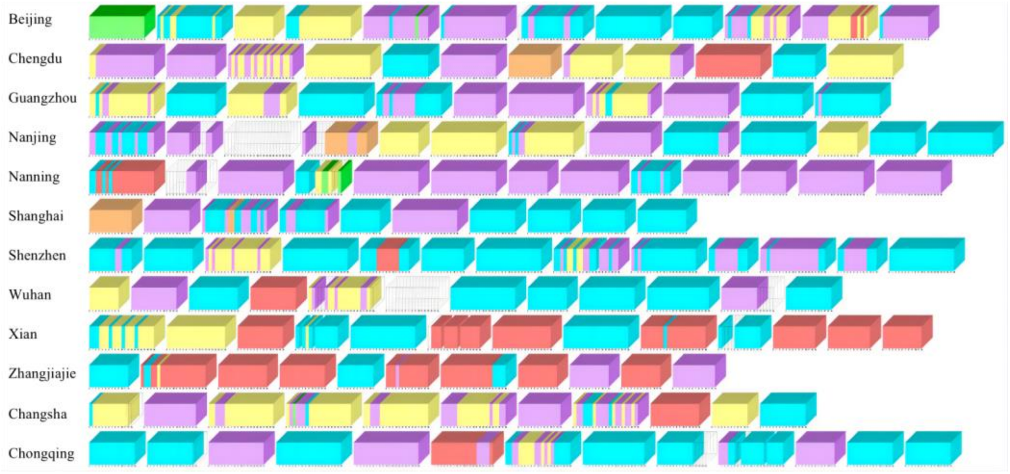

(a)

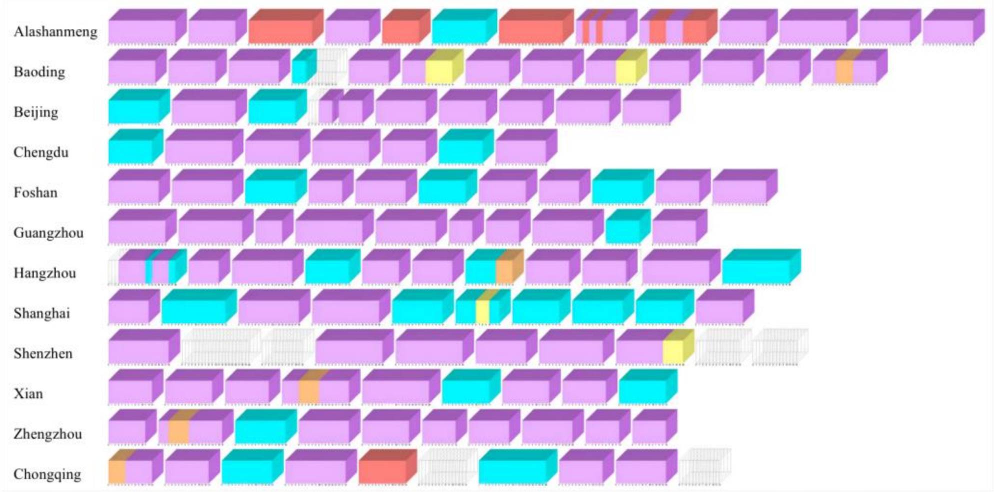

(b)

Figure 11 Amplified stacking diagram of elements based on microcube model sample case of popular city image short video;, (b) sample case of non-popular city image short video

Formatted: MDPI_5.1_figure_caption

4.5 Visualization results of element interaction of short movies based on microcube model

Based on the display data of element interactions, the number of microcube models containing six types of imagery element interactions and the number of video pumping second frames were recorded as shown in the following table.

Table 6 Number of microcube models containing element interactions and number of video pumping seconds frames

	Popular city image short video	Non-popular city image short video
Number of microcube models with element interaction	17	9
Video draw seconds frames (sheets)	150	89
Number of microcube models with dynamic interaction of elements of a certain type of imagery = 2	10	8
Video second draws (sheets)	96	67
Number of microcube models with dynamic interactive subjects > 2 for a certain type of imagery elements	7	2
Video second draws (sheets)	54	22

The number of microcubes containing interactive elements in the popular city image videos is 17, and the number of video frames is 150. Among them, the number of microcubes with dynamic interactive elements equal to 2 is 10, and the number of video

frames is 96;, the number of microcubes with dynamic interactive elements greater than 2 is 7, and the number of video frames is 54.

The number of microcubes containing interactive elements in the non-popular city image videos is 9, and the number of video frames is 89. Among them, the number of microcubes with dynamic interactive elements of a certain type is equal to 2 is 8, and the number of video frames is 67;, the number of microcubes with dynamic interactive elements of a certain type is greater than 2 is 2, and the number of video frames is 22.

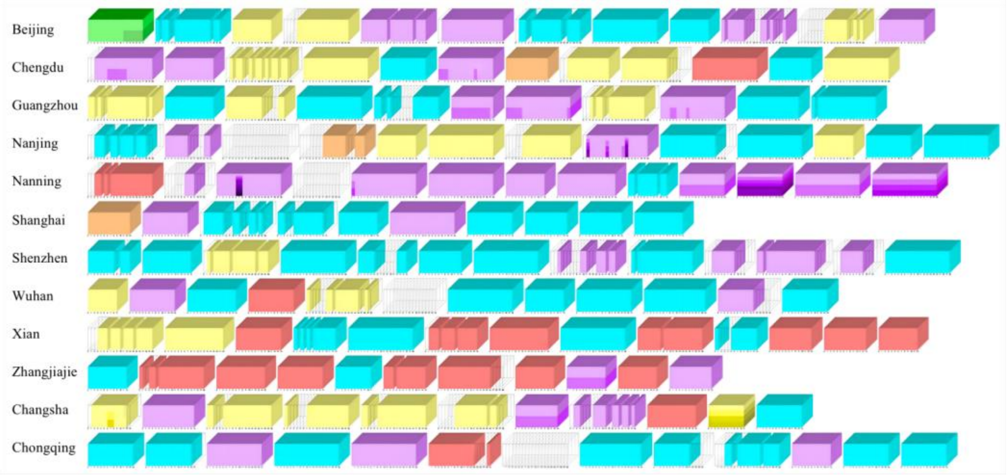

(a)

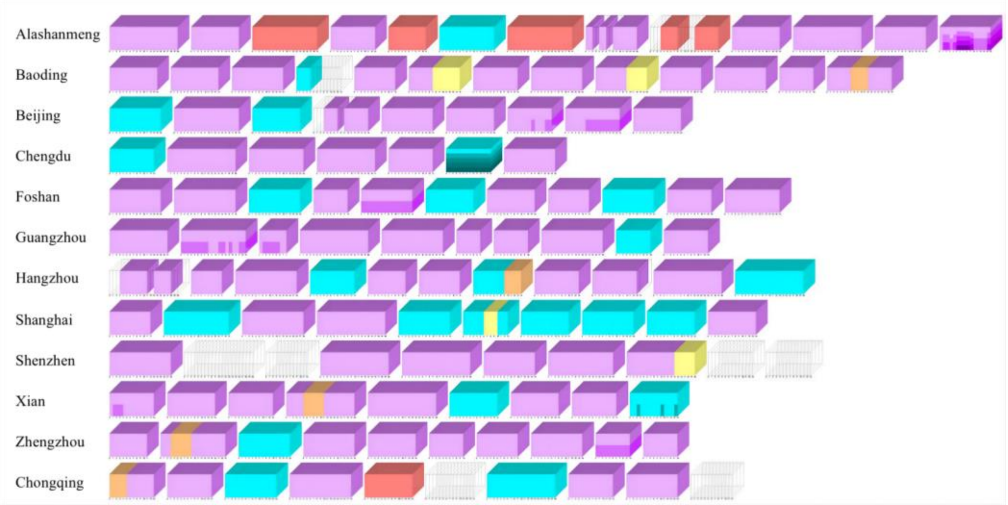

(b)

Figure 12 Element interaction stacking diagram based on microcube model (a) sample case of popular city image short video;, (b) sample case of non-popular city image short video

Formatted: MDPI_5.1_figure_caption, Indent: First line: 0"

4.6 Visualization results of short video screen style based on the microcube model

Based on the display data, the number of microcube models containing 5 types of hues and the number of video pumping seconds frames were recorded as shown in the table below.

Table 7 Number of microcube models containing unexpected events and number of video pumping seconds frames

	Popular city image short video	Non-popular city image short video
Number of microcube models with gray tones	31	25
Number of video frames in seconds (sheets)	75	26
Number of microcube models containing dark tones	43	11
Video frames (sheets)	391	128
Number of microcube models with bright tones	42	42
Video frames (pictures)	369	556
Number of microcube models with mid-tone colors	74	31
Video frames (pictures)	931	452
Number of microcubes with light tones	47	31
Video frames (sheets)	429	428

The number of microcubes with gray tones in the popular city image videos is 31, and the number of video frames is 75;, the number of microcubes with dark tones is 43, and the number of video frames is 391;, the number of microcubes with bright tones is 42, and the number of video frames is 369;, the number of microcubes with medium tones is 74, and the number of video frames is 931;, and the number of microcubes with light tones is 47, and the number of video frames is 429. The number of microcube models in lighter tones is 47 and the number of video frames is 429.

The non-popular city image videos contain 25 gray-toned microcubes and 26 video frames, 11 dark-toned microcubes and 128 video frames, 42 bright-toned microcubes and 556 video frames, 31 medium-toned microcubes and 452 video frames, and light-toned microcubes and video frames. The number of light-toned microcubes is 31 and the number of video frames is 429.

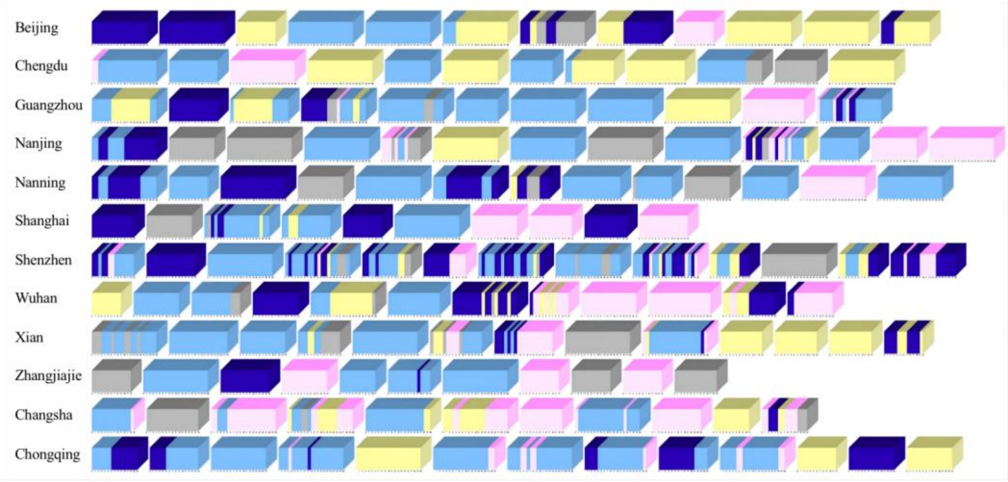

(a)

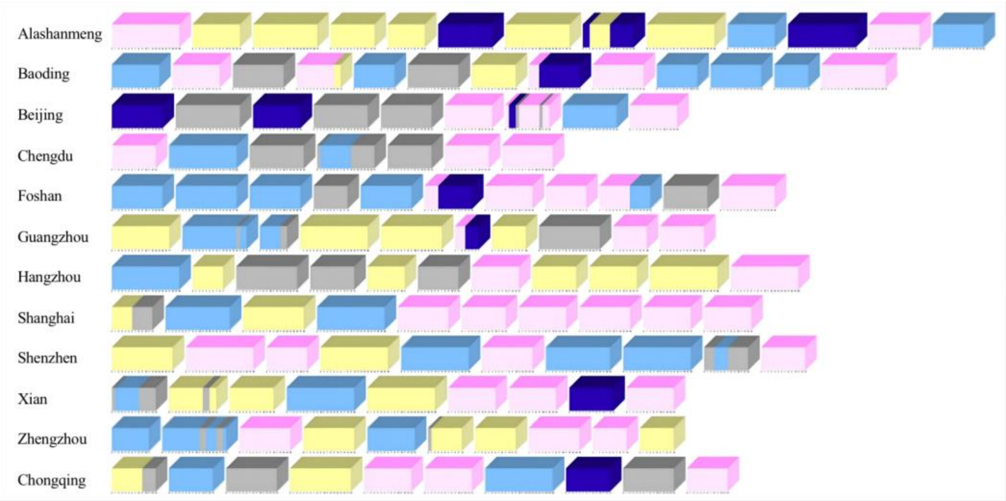

(b)

Figure 13 Screen style stacking diagram based on microcube model sample case of popular city image short video;. (b) sample case of non-popular city image short video

Formatted: MDPI_5.1_figure_caption

6 Conclusion and Discussion

Based on the microcube model analysis framework of TikTok platform data, this study explores the relationship between the content characteristics of short videos and the publicity effect of city images. The research results can not only reveal the influence mechanism of the content structure of short videos, but also provide practical guidance for the production of short videos of urban propaganda, especially in the context of strengthening the communication effect of urban brands. The research shows that: (1) the characteristics of short video content significantly affect the publicity effect of city image: dynamic features (such as unexpected events, scene switching) and static features (such as element amplification and picture style) in short video content have an important impact on the communication effect. For example, short videos with unexpected events are more likely to attract viewers' attention than short videos without unexpected events. Content with positive emotional resonance significantly increased the willingness to watch and share short videos. This suggests that creators can improve the appeal and reach of short videos by optimizing these features. (2) Emotional resonance is positively correlated with popularity: the emotional expression of short videos directly affects its communication effect. The higher the proportion of positive emotion (such as happiness, touching), the higher the popularity of the short video;. Negative emotions (such as sadness and anger) have a lower number of microcubes and tend to be less hot. This shows that the city image publicity should pay attention to the expression of positive emotions to enhance the attractiveness of short videos. (3) Reasonable scene switching and diversified elements can enhance the communication effect: the number and logic of scene switching in short videos have a significant impact on the audience's viewing experience. Too little scene switching will lead to monotonous content, while too much will increase the burden of viewing. In addition, multi-element interaction (such as the

combination of natural landscapes and human activities) can enhance the visual impact and increase the audience's interest in the image of the city.

Formatted: Indent: First line: 0", Left 0.86 ch

Although this study provides a new perspective and tool for short video content analysis, it also has the following limitations: (1) Limitations of data samples: This study mainly focuses on short city image videos on the TikTok platform as the analysis object, and the sample sources are relatively concentrated, which fails to fully cover diverse cultural contexts. Future research can be extended to other platforms (such as Instagram, YouTube) and more international cities to verify the universality of the model. (2) Lack of user behavior data: This study mainly focuses on the relationship between the content characteristics of short videos and the popularity of dissemination, but does not include the audience behavior data (such as click rate, viewing duration and interactivity) for analysis. This may limit the full understanding of the effects of short video transmission.It is verified through a large number of experiments that unexpected events, emotional resonance, scene transformation, element amplification, element interaction and picture style in short videos all play an important role in shaping the image of net celebrity cities.

(1) Unexpected events are one of the important dividing criteria between net celebrity cities and non net celebrity cities.

The Internet era is the era of attention economy, and the fundamental purpose of accidental events triggering is to stimulate users' novelty and gain their attention. The urban landscape is often treated as the scene where the short video idea occurs rather than the main content of the short video. Most of the short videos related to Netflix cities have coherent scenes and unexpected twists in the narrative, including unexpected events, while the short video scenes of non Netflix cities have flat narratives and no suspense. Compared with short videos with highly similar content, unexpected events can quickly grab the audience's attention and become the symbolic carrier of city image dissemination.

(2) Emotional resonance is the second important dividing criterion between netflix cities and non netflix cities.

The media scene has existential value only after the audience's emotion enters, and the short video can realize the real communication value only after it gets the audience's inner approval. When describing the city image, non Netflix cities regard the city image as an objective information and hardly integrate personalized experience in the process of information production. Such a promotional video narrative is too grand and lacks the interaction between people and the city, which is the core of city life, so that the audience's emotion remains neutral. Netflix cities, on the other hand, integrate individual life experiences and even personal emotions into city image shaping, producing unique city symbols to be presented on short video platforms. This often triggers the emotional factor of other users, which in turn triggers the spatial practice of others, and people construct their own "imaginary city", and the city image is formed.

(3) A reasonable number of scene transitions is the third important criterion that separates netflix cities from non netflix cities.

Different scenes have different tactile sensations, and the reasonable use of transitions can create a comfortable viewing experience for users, with a clear story line and logical content, so that users can be in the scene. Scene transitions help bring users into the virtual experience. A reasonable number of scene transitions is more likely to increase the probability of the video becoming a hit. An unreasonable number of transitions will increase the burden of users' attention, and the content will be vague and the video will be lack of memory and boring. In detail, transitions are divided into tactless transitions and tactful transitions. The tactless transition refers to the natural transition of the camera to connect the two contents, using the direct switch of the screen to transition, including the same subject transition, similar subject transition, subjective camera transition, etc.. Technique transition is the use of special effects or animation

effects to complete the switch between scenes, forming a clear paragraph level, such as fade in and fade out, superimposed, etc.. Transitions are related to the quality of the video content, and they should be more easily perceived by the audience under the premise of the logical development of the story.

(4) The attribute of amplifying elements is the fourth important demarcation criterion between Netflix cities and non-Netflix cities.

Amplifying the picture elements is to give the audience a strong visual impact. The propaganda attributes of the enlarged elements of the net-celebrity cities are clear, while the attributes of the elements of the non-net-celebrity cities are not clear enough. In the case, the enlarged elements in both the popular city image short video and the non-popular city image short video are food, and the geographical and category matching of this food element, the food element of the popular city has a clear attribute and is unforgettable; while the food element of the non-popular city has an unclear memory point. The fundamental reason for the element amplification after the attribute matching triggers attention is that these elements tend to focus on social hot elements, social stubborn elements, lost elements, label elements, etc. And amplifying elements combined with network bursts for precise promotion will achieve twice the effect with half the effort. The amplification of elements in the city image promotion film can make the city very expressive and the city image more three-dimensional, and then can be distinctive and unique among similar cities.

(5) The decomposition rate of the amplified elements is the fifth important criterion that separates the net-worthy cities from the non-net-worthy cities.

The fundamental purpose of element interaction is to bring the dynamic form of elements to the extreme, bringing users a fresher visual experience while always stimulating their visual nerves. For short videos where the amplifying elements are all character elements, the decomposed elements of Netflix City reflect the interaction between characters. Taking a couple as an example, from meeting to hugging, the squares composing the original characters overlap from no overlap to a certain degree of overlap at the moment of hugging, and the visualization color also changes from OE characters to DE characters 1+DE characters 2, bringing a dynamic sense of vision. In contrast, the decomposed elements of non-Netflix cities are relatively low, the extreme example is in the form of pictures coherent into short videos, with no interaction between elements from beginning to end, and the static visual sense brought in the dynamic presentation of short videos is not enough to impress the viewers. The interaction between elements makes the video easy and interesting, so that the city image publicity short video is no longer a serious narrative propaganda, opening up a new channel for the expression of video emotion, different interactions often carry different emotions or different views of expression, reasonable interaction of elements will increase the probability of user favorite.

(6) The type of screen style is the sixth important dividing criterion between netflix cities and non-netflix cities.

At present, most of the contents of short videos define the style of videos from users' needs, and meet users' tastes with flirtatious and playful tones, which also leads to a uniform and uninspired style of short videos. Therefore, the fundamental purpose of constructing a distinctive short video style is to let users have a unique memory point of the city. Most of the short videos of city images of Netflix cities focus on aesthetic pursuit, with elaborate composition and exquisite variety in the control of tones and scenery, each frame can become a landscape with full texture, and the whole picture is very ritualistic. The city image short video of non-net red cities is often a simple accumulation of original video material, or a pile of special effects, and the picture is often hard to see. The city image short video with its own style can not only meet users' visual expectation and give them visual enjoyment, but also guide their aesthetics and enhance their memory of the city image short video, and then internalize the city image in their hearts.

Funding

Supported by Key Laboratory of Spatial Data Mining & Information Sharing of Ministry of Education, Fuzhou University (No.2023LSDMIS02)

Think Tank for the Construction of Ecological Barrier in the Upper Reaches of the Yangtze and Yellow Rivers in Sichuan Province: Visual analysis of multi-level spatiotemporal data fused with multimodal information (No.202209)

Anhui Cultural Tourism Innovative Development Research Institute: Research on smart tourism innovation based on big data -- building a new scenario of cultural tourism and technology integration (No.ACTK2022YB01)

The funders had no role in study design, data collection and analysis, decision to publish, or preparation of the manuscript.

Conflicts of Interest

The authors declare no conflict of interest.

Data Availability Statement

The collection and analysis method complied with the terms and conditions for the source of the data. All data underlying the findings described in their manuscript to be freely available to other researchers.

Formatted: Font color: Red

Formatted: Font: Bold, (Asian) Chinese (PRC)

Formatted: Font:

Formatted: Indent: Left: 1.81"

Formatted: MDPI_3.1_text

References

1. Huang Feng. An analysis of the image characteristics and filming techniques of short video feature reports[J]. New Media Research,2021,7(16):33-35.

2. Qin Feng, Zhou Yujin. Mobile short video content production and optimization strategy based on user comments[J]. China TV,2022(06):92-96.

~~2.~~3. Li M , Mohamed S , Xu H ,et al.DIALOGUE OR MONOLOGUE: A STUDY ON PUBLIC PARTICIPATION IN URBAN IMAGE COMMUNICATION BASED ON SOCIAL MEDIA[J].World conference on media and mass communication, 2023.DOI:10.17501/24246778.2023.7103.

~~3.~~4. Cheng Qianqian. Story, immersion and interaction: the realization path of effective dissemination of short videos of red documents in libraries[J]. Sichuan Library Journal,2022(04):75-79.

~~4.~~5. Huang Xiaoyin,Qiu Zihao. Technology Empowerment and Emotional Interaction:A Study of Visual Music Dissemination on Jieyin Platform[J]. Journal of Southwest University for Nationalities (Humanities and Social Sciences Edition),2019,40(08):156-161.

~~5.~~6. Chen Xintong. Exploring the success of the short food video "Japanese Food"[J]. Modern Communication,2020(10):122-123.

7. Su Qiushi, Liu Caizhi. Research on the application of transition technology in film and television post-production[J]. Computer Knowledge and Technology,2017,13(12):181-184.

~~6.~~8. Wang B .The Role of Data Fusion in Contemporary Urban Image Design and Communication in the Context of Big Data[J].Applied Mathematics and Nonlinear Sciences, 2024, 9(1).DOI:10.2478/amns-2024-2517.

~~7.~~9. Sun Zhenhu,Zhang Yue,Wei Linlu. Transition - the "secret weapon" of smooth video expression [J]. Journalism and Writing, 2020(03):105-108.

10. Yan Hanmei. Analysis of short video production skills[J]. Western Radio and Television, 2022,43(07):69-71.

~~8.~~11. Zhou Y , Wang Z , Zheng S ,et al.Optimization of automated garbage recognition model based on ResNet-50 and weakly supervised CNN for sustainable urban development[J].Alexandria Engineering Journal, 2024, 108:415-427.DOI:10.1016/j.aej.2024.07.066.

~~9.~~12. Chen Xin. Research on short video production innovation in the context of new media [J]. Western Radio and Television,2019(11):15+19.

~~10.~~13. Wang Xiang. The influence of picture language and detail processing on the narrative of TV investigative reporting [J]. News Knowledge,2012(12):79-81.

~~11.~~14. Fan Yuanyuan. Breakfast China: A new journey in the creation of short food documentaries[J]. Media Forum,2020,3(11):10-11+14.

~~12.~~15. Yang Jiesong. The use of camera language in mainstream media short video products[J]. News Front,2022(14):113-115.

~~13.~~16. Xu Jian. Research on the visual communication of short videos on Weibo [D]. Zhengzhou University,2019.

17. Fan Jingting. Analysis of the creative techniques of food documentaries--Taking "Eclipse" as an example[J]. Audiovisual,2019(02):42-43.

~~14.~~18. Yan L , Guofeng H , Miaochao C .Design and Communication of City Brand Image Based on Big Data and Personalized Recommendation System[J].Journal of Function Spaces, 2022, 2022(Pt.9).

19. Shen Yang. Refinement of short video production in the new media environment[J]. News Outpost,2020(01):50-51.

~~15.~~20. Shedid M Y , Hefnawy N H .Historic Preservation as a Tool for City Branding Case Study: Khedivial Cairo[J]. 2022.DOI:10.1007/978-3-030-74482-3_23.
~~16.~~21. Hao Xiaoyuan. Content features and problems of mobile short videos[J]. Communication and Copyright,2017(03):85-87.
~~17.~~22. Shen Qingbin. Analysis of the "simplicity" of NHK documentary images[J]. Audiovisual,2019(12):142-143.
23. Pan Lu. Analysis of the creation of urban documentaries in the short video era[J]. Film Literature,2021(23):45-47.
~~18.~~24. Lin M , Qiming Y .Research on the Application of Short Video in City Image Display[J].International Journal of Science and Engineering Applications, 2023.DOI:10.7753/ijsea1201.1042.
~~19.~~25. Cui Guobin. The selection of originality criteria for live sports event screen [J]. Journal of Soochow University (Law Edition),2019,6(04):1-12.
~~20.~~26. Peng W H, Walls F G, Cohen R A, et al. Overview of screen content video coding: technologies, standards, and beyond[J]. IEEE Journal on Emerging and Selected Topics in Circuits and Systems, 2016, 6(4): 393-408.
~~21.~~27. Wang Zhen. Exploring the practice of shooting current affairs news television screen[J]. News Culture Construction, 2020(06):51-52.
~~22.~~28. Zhang Jieliang, Jiang Weifang, Wang Xin, Zhao Liang. Review of video conference screen quality evaluation methods[A]. China Command and Control Society. Proceedings of the Sixth China Command and Control Conference (Previous Book) [C]. Chinese Command and Control Society:Chinese Command and Control Society,2018:5.

Review Comments to the Author

Please use the space provided to explain your answers to the questions above. You may also include additional comments for the author, including concerns about dual publication, research ethics, or publication ethics. (Please upload your review as an attachment if it exceeds 20,000 characters)

Reviewer #1:

Authors attempt to work on a very interesting topic regarding showcasing cities for the purpose of popularity and prosperity however the real problem is not clear as to why this work is necessary.

Response: Thank the reviewers for their careful reading and valuable comments on this article. We recognize that there is room for improvement in clarifying the context and necessity of the study. In the process of revision, we re-sorted out the problem consciousness and goal of the research, further supplemented the specific description of the research background and the potential application value of this research in the analysis of short video content of urban image promotion.

It is recommended that authors should remove the sections indicated in abstract but only retain the logical structure. Also, the introduction section looks shallow and the background to this study requires more robust introduction section reflecting the real problem with global relevance for proper perspective.

Response: Thanks to the reviewers for their pertinent suggestions on this paper, especially for the guidance on simplifying the content of the abstract and perfecting the background of the introduction. We recognize that the current summary is somewhat redundant in the description of technical details, which may reduce the clarity of the core logical structure. According to the recommendations of the reviewers, we have deleted technical expressions in the revision and highlighted the logical thread in order to more directly demonstrate the research objectives and significance. In addition, as for the problems in the introduction, we are deeply aware that the original text lacks in the breadth and depth of the research background, especially in the context of globalization, the expression of relevance still needs to be strengthened. To this end, we supplement the status quo and challenges of global urban brand communication, and combine the communication characteristics of short video, a new medium, to clarify the practical significance and scope of application of this study. It is hoped that these modifications can better reflect the overall logic and research value of the paper.

It is important to reflect on how such framework could affect tourism either positively or negatively due to the fact that some cities are dream land to many globally and the moment they view such videos it could impact their decisions in both ways hence the framework must consider this effect.

Response: Thanks to the reviewers for their suggestions on this paper, especially the concern that the research framework may influence travel decisions. We recognize that in the context of globalization, the potential positive and negative effects of short video content on the tourism industry deserve further discussion. In the revision, we have added relevant background information in the introduction, clearly pointing out the two-way possibilities of short videos in attracting tourists and affecting the image of the city. At the same time, in the discussion part, the specific analysis of the influence of the framework on tourism decision-making is added, including the possibility of positive and negative effects and countermeasures.

The source of the TikTok video is missing even though authors stated that they were verified. Where were this data stored for the purpose of this research among other data integrity concerns.

Response: Thank the reviewers for their attention to the source and completeness of the data in this paper. These issues are crucial to the credibility of the research, and we have made the following improvements in the revision: In the part of methodology, we detailed the collection and verification process of TikTok video data, including screening criteria, verification mechanism and denoising methods, to ensure the authenticity and representativeness of the data, and added specific measures for data storage and integrity protection.

While commending the analysis of cases and extensive review carried out by authors figures e.g. figure 7 is not clear enough to relate with as much as readership requires great improvement. It is recommended that authors should device an alternative means of representing this fact.

Response: Figure 7 has been reuploaded and improved to clearly label the variable meanings of each column and row, while ensuring good readability in both digital and print media by increasing color contrast and labeling.

For clarity it is important for the authors to discuss results separately from conclusion as it looks mixed up and vague thereby making it difficult to see in clear terms what achievement is recorded by this work.

Response: Thanks to the reviewers for their meticulous feedback on the structure of this paper. We recognize that mixing "results" with "conclusions" can affect readers' understanding of research findings and summaries. To this end, during the revision process, we reorganized the paper to include a separate "results" section to elaborate on specific findings based on the data analysis; At the same time, the "conclusion" part is adjusted to summarize the significance, practical application, limitations and future direction of the research.

Grammatical flow should be checked by authors for consistency.

Response: Thanks to the reviewers for their valuable comments on the grammatical coherence of this paper. We are deeply aware of the importance of grammatical accuracy and fluency in the presentation of academic articles. Based on your suggestions, we have made checked by authors for consistency to the full text.

Reviewer #2:

A framework for visualizing and describing city image promotion short video data based on microcube model.
Reviewed by: Riyaz Abro.
Observations (If appropriate & Applicable).

• Abstract:

o Which software was used to analyse the data, include timeframe and time limit of the data that from what time and period the data was collected.

Response: Thanks to the reviewers for their valuable suggestions on the summary. We recognize that clarity about the software tools used for data analysis and the time frame of the data helps improve transparency and reproducibility. In response to your suggestions, we have supplemented the data sources and research methods in the Summary and Methods section. Regarding the software used for data analysis, we utilized Python with pandas for data manipulation, scikit-learn for statistical analysis, and matplotlib for visualization. Additionally, deep learning techniques based on PyTorch were employed. Detailed descriptions related to the data are provided in Chapter 4 and Table 1.

• Introduction:

o Already given variables in keywords are different than the defined ones

Response: "City image publicity" has been changed to "city image promotion", and "microscopic content analysis" have been modified to "Micro Perspective".

• Literature Review:

o Given variables must be defined (if possible).

Response: We have revised the manuscript to include precise definitions of the key variables, such as "unexpected events," "emotional resonance," "scene transition," "elemental amplification," "element interaction," and "screen style." These definitions have been integrated into the literature review section to enhance clarity and provide a better understanding of their role in our research framework.

• Precise Conclusion:

o Limitation must be defined, hypotheses must be generated separately, however, paper is very much innovative and be published with minor correction as suggested and if deemed appropriate.

Response: Thank you very much for your insightful comments regarding the need for a

precise conclusion. Based on your suggestions, we have further refined the "Conclusion and Discussion" section to clearly define the limitations of our study and have also generated the hypotheses separately. By doing so, we aim to provide a more structured and clear presentation of our research contributions.

Reviewer #3:

1. Explain the immersion methos or techniques based on micro cube model

Response: The immersion method based on the microcube model aims to analyze and enhance the viewer's immersive experience by breaking down short video content into multiple small content cube units. This model refines the video content by segmenting, assigning, and visualizing key elements within the short video, thereby exploring the impact of these elements on audience emotions and engagement.

2. Also explain how all the parameters are interrelated with each other

Response: The parameters identified in this study—unexpected events, emotional resonance, scene transition, element amplification, element interaction, and screen style—are deeply interrelated, working together to enhance the effectiveness of short videos in promoting city images.

A video that starts with an unexpected event can use emotional resonance to keep viewers engaged, turning their initial curiosity into a deeper emotional connection. Effective use of scene transitions combined with amplified elements can enhance the narrative clarity and visual impact, making the content more memorable. A well-defined screen style enhances element interactions by creating a cohesive visual experience that resonates with viewers, making the city image more distinctive. Each variable influences the others. For example, an unexpected event can be more impactful if accompanied by an emotionally resonant scene, amplified elements, and smooth transitions. Together, these parameters create a holistic effect, making the short video more engaging, memorable, and effective in conveying a city's image. The absence of any one variable can reduce the overall effectiveness, as each plays a crucial role in maintaining viewer interest and enhancing the video's communicative power.

3.One absence of parameter will affect the performance of the short videos

Response: Yes, the absence of any parameter will impact the overall performance of the short video. Including unexpected events in short videos can spark curiosity and quickly capture the audience's interest. Emotional resonance is a key factor in maintaining audience engagement, as emotion-driven content is critical for successful video dissemination. Proper scene transitions help maintain the video's pacing and narrative coherence, allowing viewers to immerse themselves more easily. Element amplification aims to capture viewers' attention through visual impact. Element interaction enhances the video's fun and dynamic presentation, making it more engaging and livelier. A unique screen style helps a video stand out among numerous others, creating a distinct memory point.

Reviewer #4:

This paper uses the Microcube model framework to calculate the unit video content and analyze the packaging time in each microcube, which provides a novel perspective for city image promotion and has certain research value, but there are still the following shortcomings, which I hope to improve:

1. Literature review part of the literature, mainly focused on China literature research, the lack of a broader field and country, the review of relevant research results. In view of the globalization background of short video and urban communication and promotion, the research results of international scholars in the fields of short video content and communication mode should be introduced appropriately. This will help to enhance the global perspective and academic depth of this study, and more comprehensively demonstrate the applicability and innovation of the study on a global scale.

Response: Thanks to the reviewers for their suggestions on the improvement of relevant work parts of this paper. We recognize that the addition of new academic research to each research topic not only improves the depth of the literature review, but also enhances the academic and

theoretical support of the paper. According to your suggestions, we have added references from relevant global scholars' research results in six dimensions, namely, accident, emotional resonance, scene switching, element amplification, element interaction, and picture style.

2. Short videos are divided into six categories, but no specific classification basis is provided. The current classification may not fully cover the diversity of short videos, especially when the differences in content and communication targets increase. It is suggested to provide clearer classification standards and consider how to incorporate other types of video content into the classification system to enhance the scientificity and applicability of classification.

Response: We have revised the manuscript and provided detailed definitions of the six imagery elements. These elements are not intended for classifying short videos but were selected due to their significant impact on short video content. Therefore, they serve as the core research dimensions in the analysis framework for city image promotion short videos. These elements were derived from a comprehensive analysis of existing literature and empirical data, allowing us to better reveal the influence of short video content characteristics on city image promotion.

3. The correspondence between color and concept needs to be explained: the study used color to distinguish different elements when discussing short video content, but did not explain in detail how the color corresponds to specific elements.

Response: In the manuscript, we described how specific elements are represented using colors. For example, experimentally, we designed an emotion grid chart with nine emotions: moved, praised, funny, happy, neutral, angry, sad, shocked, and helpless. We used #EE6363, #EEDFCC, #FFA54F, #FFEC8B, #FFFFF0, #00F5FF, #63B8FF, #00FF7F, and #CD69C9 as RGB color controls, respectively, as shown in Figure 2(a). We designed an imagery elements grid with six elements: technology elements, food elements, natural landscape elements, urban landscape elements, people elements, animal elements. We used #00FF7F, #FFEC8B, #EE6363, #00F5FF, #EE82EE, #FFA54F as RGB color controls, as shown in Figure 4(a). And a grid diagram of the main colors of the screen style was designed, and #0000FF, #BEBEBE, #FFFACD, #6495ED, and #FFC0CB were used as RGB color controls for dark, gray, light, medium, and bright tones, respectively, as shown in Figure 6(a).

4. There is a lack of transparency in the process of constructing the micro-cube model. The micro-cube model is one of the core methods in this study, but at present, the description of the model construction process is abstract, and the existing algorithm (data slicing? Or is it an optimization algorithm for slicing? Or OLAP and multidimensional data modeling?) or self-construction algorithm, the paper does not explain in detail, and the listed algorithms lack detailed explanation. In order to improve the transparency and reproducibility of the model, I hope the author can explain it in detail and provide specific procedures, formulas and data support for the model construction. This will facilitate colleagues to better understand and verify the operability and effectiveness of the model.

Response: Thank you for the your suggestion. The model construction process has been addressed in the manuscript. Specifically, we adopt a micro perspective to refine the video content by establishing a content microcube model that utilizes data slicing techniques to segment the complete short video into multiple fragmented units based on time intervals. Each microcube encapsulates the content per unit of time, as illustrated in Figure 1(a).

5. Scientific methods in the process of short video classification: At present, short video classification methods lack systematic scientific basis and rely on subjective judgment, which may affect the accuracy of classification and the rigor of research results. It is suggested that a more systematic classification method should be introduced in the classification process.

Response: Thank you to the reviewer for your valuable suggestions on this paper. To improve efficiency, we used deep learning techniques for short video classification. However, the focus of this study is on the application of the microcube model, rather than exploring how to use deep learning techniques for video classification. Therefore, the classification process is not elaborated in the manuscript, and only the classification results are presented. We hope the reviewer understands the primary focus and direction of our research. Once again, thank you for your suggestions.
