# Supplementary material for: A framework for visualizing and describing city image promotion short video data based on microcube model
Source: PLoS One. 2025 Apr 10;20(4):e0317883. doi: 10.1371/journal.pone.0317883 (PMC11984742; doi:10.1371/journal.pone.0317883)
Supplement: Attachment — Submitted filename: Response.docx [file pone.0317883.s003.docx]

Review Comments to the Author

Please use the space provided to explain your answers to the questions above. You may also include additional comments for the author, including concerns about dual publication, research ethics, or publication ethics. (Please upload your review as an attachment if it exceeds 20,000 characters)

Reviewer #1:

Authors attempt to work on a very interesting topic regarding showcasing cities for the purpose of popularity and prosperity however the real problem is not clear as to why this work is necessary.
**Response:** Thank the reviewers for their careful reading and valuable comments on this article. We recognize that there is room for improvement in clarifying the context and necessity of the study. In the process of revision, we re-sorted out the problem consciousness and goal of the research, further supplemented the specific description of the research background and the potential application value of this research in the analysis of short video content of urban image promotion.

It is recommended that authors should remove the sections indicated in abstract but only retain the logical structure. Also, the introduction section looks shallow and the background to this study requires more robust introduction section reflecting the real problem with global relevance for proper perspective.
**Response:** Thanks to the reviewers for their pertinent suggestions on this paper, especially for the guidance on simplifying the content of the abstract and perfecting the background of the introduction. We recognize that the current summary is somewhat redundant in the description of technical details, which may reduce the clarity of the core logical structure. According to the recommendations of the reviewers, we have deleted technical expressions in the revision and highlighted the logical thread in order to more directly demonstrate the research objectives and significance. In addition, as for the problems in the introduction, we are deeply aware that the original text lacks in the breadth and depth of the research background, especially in the context of globalization, the expression of relevance still needs to be strengthened. To this end, we supplement the status quo and challenges of global urban brand communication, and combine the communication characteristics of short video, a new medium, to clarify the practical significance and scope of application of this study. It is hoped that these modifications can better reflect the overall logic and research value of the paper.

It is important to reflect on how such framework could affect tourism either positively or negatively due to the fact that some cities are dream land to many globally and the moment they view such videos it could impact their decisions in both ways hence the framework must consider this effect.

**Response:** Thanks to the reviewers for their suggestions on this paper, especially the concern that the research framework may influence travel decisions. We recognize that in the context of globalization, the potential positive and negative effects of short video content on the tourism industry deserve further discussion. In the revision, we have added relevant background information in the introduction, clearly pointing out the two-way possibilities of short videos in attracting tourists and affecting the image of the city. At the same time, in the discussion part, the specific analysis of the influence of the framework on tourism decision-making is added, including the possibility of positive and negative effects and countermeasures.

The source of the TikTok video is missing even though authors stated that they were verified. Where were this data stored for the purpose of this research among other data integrity concerns.
**Response:** Thank the reviewers for their attention to the source and completeness of the data in this paper. These issues are crucial to the credibility of the research, and we have made the following improvements in the revision: In the part of methodology, we detailed the collection and verification process of TikTok video data, including screening criteria, verification mechanism and denoising methods, to ensure the authenticity and representativeness of the data, and added specific measures for data storage and integrity protection.

While commending the analysis of cases and extensive review carried out by authors figures e.g. figure 7 is not clear enough to relate with as much as readership requires great improvement. It is recommended that authors should device an alternative means of representing this fact.
**Response:** Figure 7 has been reuploaded and improved to clearly label the variable meanings of each column and row, while ensuring good readability in both digital and print media by increasing color contrast and labeling.

For clarity it is important for the authors to discuss results separately from conclusion as it looks mixed up and vague thereby making it difficult to see in clear terms what achievement is recorded by this work.

**Response:** Thanks to the reviewers for their meticulous feedback on the structure of this paper. We recognize that mixing "results" with "conclusions" can affect readers' understanding of research findings and summaries. To this end, during the revision process, we reorganized the paper to include a separate "results" section to elaborate on specific findings based on the data analysis; At the same time, the "conclusion" part is adjusted to summarize the significance, practical application, limitations and future direction of the research.

Grammatical flow should be checked by authors for consistency.

**Response:** Thanks to the reviewers for their valuable comments on the grammatical coherence of this paper. We are deeply aware of the importance of grammatical accuracy and fluency in the presentation of academic articles. Based on your suggestions, we have made checked by authors for consistency to the full text.

Reviewer #2:

A framework for visualizing and describing city image promotion short video data based on microcube model.
Reviewed by: Riyaz Abro.
Observations (If appropriate & Applicable).
• Abstract:
o Which software was used to analyse the data, include timeframe and time limit of the data that from what time and period the data was collected.

**Response:** Thanks to the reviewers for their valuable suggestions on the summary. We recognize that clarity about the software tools used for data analysis and the time frame of the data helps improve transparency and reproducibility. In response to your suggestions, we have supplemented the data sources and research methods in the Summary and Methods section. Regarding the software used for data analysis, we utilized Python with pandas for data manipulation, scikit-learn for statistical analysis, and matplotlib for visualization. Additionally, deep learning techniques based on PyTorch were employed. Detailed descriptions related to the data are provided in Chapter 4 and Table 1.

• Introduction:
o Already given variables in keywords are different than the defined ones

**Response:** "City image publicity" has been changed to "city image promotion", and "microscopic content analysis" have been modified to "Micro Perspective".

• Literature Review:
o Given variables must be defined (if possible).

**Response:** We have revised the manuscript to include precise definitions of the key variables, such as "unexpected events," "emotional resonance," "scene transition," "elemental amplification," "element interaction," and "screen style." These definitions have been integrated into the literature review section to enhance clarity and provide a better understanding of their role in our research framework.

• Precise Conclusion:
o Limitation must be defined, hypotheses must be generated separately, however, paper is very much innovative and be published with minor correction as suggested and if deemed appropriate.

**Response:** Thank you very much for your insightful comments regarding the need for a precise conclusion. Based on your suggestions, we have further refined the "Conclusion and Discussion" section to clearly define the limitations of our study and have also generated the hypotheses separately. By doing so, we aim to provide a more structured and clear presentation of our research contributions.

Reviewer #3:

1. Explain the immersion methos or techniques based on micro cube model

**Response:** The immersion method based on the microcube model aims to analyze and enhance the viewer's immersive experience by breaking down short video content into multiple small content cube units. This model refines the video content by segmenting, assigning, and visualizing key elements within the short video, thereby exploring the impact of these elements on audience emotions and engagement.

2. Also explain how all the parameters are interrelated with each other

**Response:** The parameters identified in this study—unexpected events, emotional resonance, scene transition, element amplification, element interaction, and screen style—are deeply interrelated, working together to enhance the effectiveness of short videos in promoting city images.

A video that starts with an unexpected event can use emotional resonance to keep viewers engaged, turning their initial curiosity into a deeper emotional connection. Effective use of scene transitions combined with amplified elements can enhance the narrative clarity and visual impact, making the content more memorable. A well-defined screen style enhances element interactions by creating a cohesive visual experience that resonates with viewers, making the city image more distinctive. Each variable influences the others. For example, an unexpected event can be more impactful if accompanied by an emotionally resonant scene, amplified elements, and smooth transitions. Together, these parameters create a holistic effect, making the short video more engaging, memorable, and effective in conveying a city’s image. The absence of any one variable can reduce the overall effectiveness, as each plays a crucial role in maintaining viewer interest and enhancing the video’s communicative power.

3.One absence of parameter will affect the performance of the short videos

**Response:** Yes, the absence of any parameter will impact the overall performance of the short video. Including unexpected events in short videos can spark curiosity and quickly capture the audience's interest. Emotional resonance is a key factor in maintaining audience engagement, as emotion-driven content is critical for successful video dissemination. Proper scene transitions help maintain the video's pacing and narrative coherence, allowing viewers to immerse themselves more easily. Element amplification aims to capture viewers' attention through visual impact. Element interaction enhances the video's fun and dynamic presentation, making it more engaging and livelier. A unique screen style helps a video stand out among numerous others, creating a distinct memory point.

Reviewer #4:

This paper uses the Microcube model framework to calculate the unit video content and analyze the packaging time in each microcube, which provides a novel perspective for city image promotion and has certain research value, but there are still the following shortcomings, which I hope to improve:
1. Literature review part of the literature, mainly focused on China literature research, the lack of a broader field and country, the review of relevant research results. In view of the globalization background of short video and urban communication and promotion, the research results of international scholars in the fields of short video content and communication mode should be introduced appropriately. This will help to enhance the global perspective and academic depth of this study, and more comprehensively demonstrate the applicability and innovation of the study on a global scale.

**Response:** Thanks to the reviewers for their suggestions on the improvement of relevant work parts of this paper. We recognize that the addition of new academic research to each research topic not only improves the depth of the literature review, but also enhances the academic and theoretical support of the paper. According to your suggestions, we have added references from relevant global scholars' research results in six dimensions, namely, accident, emotional resonance, scene switching, element amplification, element interaction, and picture style.

2. Short videos are divided into six categories, but no specific classification basis is provided. The current classification may not fully cover the diversity of short videos, especially when the differences in content and communication targets increase. It is suggested to provide clearer classification standards and consider how to incorporate other types of video content into the classification system to enhance the scientificity and applicability of classification.

**Response:** We have revised the manuscript and provided detailed definitions of the six imagery elements. These elements are not intended for classifying short videos but were selected due to their significant impact on short video content. Therefore, they serve as the core research dimensions in the analysis framework for city image promotion short videos. These elements were derived from a comprehensive analysis of existing literature and empirical data, allowing us to better reveal the influence of short video content characteristics on city image promotion.

3. The correspondence between color and concept needs to be explained: the study used color to distinguish different elements when discussing short video content, but did not explain in detail how the color corresponds to specific elements.

**Response:** In the manuscript, we described how specific elements are represented using colors. For example, experimentally, we designed an emotion grid chart with nine emotions: moved, praised, funny, happy, neutral, angry, sad, shocked, and helpless. We used #EE6363, #EEDFCC, #FFA54F, #FFEC8B, #FFFFF0, #00F5FF, #63B8FF, #00FF7F, and #CD69C9 as RGB color controls, respectively, as shown in Figure 2(a). We designed an imagery elements grid with six elements: technology elements, food elements, natural landscape elements, urban landscape elements, people elements, animal elements. We used #00FF7F, #FFEC8B, #EE6363, #00F5FF, #EE82EE, #FFA54F as RGB color controls, as shown in Figure 4(a). And a grid diagram of the main colors of the screen style was designed, and #0000FF, #BEBEBE, #FFFACD, #6495ED, and #FFC0CB were used as RGB color controls for dark, gray, light, medium, and bright tones, respectively, as shown in Figure 6(a).

4. There is a lack of transparency in the process of constructing the micro-cube model. The micro-cube model is one of the core methods in this study, but at present, the description of the model construction process is abstract, and the existing algorithm (data slicing? Or is it an optimization algorithm for slicing? Or OLAP and multidimensional data modeling? ) or self-construction algorithm, the paper does not explain in detail, and the listed algorithms lack detailed explanation. In order to improve the transparency and reproducibility of the model, I hope the author can explain it in detail and provide specific procedures, formulas and data support for the model construction. This will facilitate colleagues to better understand and verify the operability and effectiveness of the model.

**Response:** Thank you for the your suggestion. The model construction process has been addressed in the manuscript. Specifically, we adopt a micro perspective to refine the video content by establishing a content microcube model that utilizes data slicing techniques to segment the complete short video into multiple fragmented units based on time intervals. Each microcube encapsulates the content per unit of time, as illustrated in Figure 1(a).

5. Scientific methods in the process of short video classification: At present, short video classification methods lack systematic scientific basis and rely on subjective judgment, which may affect the accuracy of classification and the rigor of research results. It is suggested that a more systematic classification method should be introduced in the classification process.

**Response:** Thank you to the reviewer for your valuable suggestions on this paper. To improve efficiency, we used deep learning techniques for short video classification. However, the focus of this study is on the application of the microcube model, rather than exploring how to use deep learning techniques for video classification. Therefore, the classification process is not elaborated in the manuscript, and only the classification results are presented. We hope the reviewer understands the primary focus and direction of our research. Once again, thank you for your suggestions.
