# Supplementary material for: A framework for visualizing and describing city image promotion short video data based on microcube model
Source: PLoS One. 2025 Apr 10;20(4):e0317883. doi: 10.1371/journal.pone.0317883 (PMC11984742; doi:10.1371/journal.pone.0317883)
Supplement: Attachment — Submitted filename: Response_auresp_2.docx [file pone.0317883.s004.docx]

Review Comments to the Author

Please use the space provided to explain your answers to the questions above. You may also include additional comments for the author, including concerns about dual publication, research ethics, or publication ethics. (Please upload your review as an attachment if it exceeds 20,000 characters)

Reviewer #1: The feedback is noted and full data could be made available to the editor. Authors have done well in addressing most of the concerns raised.

**Response：**Thank you very much for your kind and encouraging feedback. We are pleased to hear that our revisions have addressed most of your concerns. Your constructive comments have significantly improved the clarity, rigor, and depth of our work. We deeply appreciate your time, effort, and valuable insights throughout the review process.

Reviewer #2: A framework for visualizing and describing city image promotion short video data based on microcube model.
Reviewed by: Riyaz Abro.
Observations (If appropriate & Applicable).
• Abstract:
o Which software was used to analyse the data, include timeframe and time limit of the data that from what time and period the data was collected.
• Introduction:
o Already given variables in keywords are different than the defined ones
• Literature Review:
o Given variables must be defined (if possible).
• Precise Conclusion:
o Limitation must be defined, hypotheses must be generated separately, however, paper is very much innovative and be published with minor correction as suggested and if deemed appropriate.

**Response：**Thank you very much for your meticulous review and valuable suggestions on our manuscript. Your insightful comments regarding the inclusion of data analysis software, data timeframe, and collection period in the abstract, alignment of keywords with defined variables in the introduction, clear definitions of variables in the literature review, and the separation of hypotheses and limitations in the conclusion have been extremely helpful in improving the quality of our work. We have carefully addressed your feedback by specifying the software used (Python, Pandas, Matplotlib) and the data collection timeframe (July 2019 to December 2019) in the abstract. In the introduction, we have ensured consistency between the keywords and the defined variables. The literature review now includes clear definitions of the core variables, each supported by relevant references. In the conclusion, we have separately outlined the hypotheses, clarified the study's limitations, and highlighted its innovative contributions. Once again, thank you for your recognition of our research and your constructive guidance. We hope the revised manuscript meets your expectations and look forward to your further review and feedback.

Reviewer #3: (No Response)

Reviewer #4: Partial Literature Review: The literature review section is somewhat superficial and does not sufficiently cover the current state of research on urban image promotion through short videos and the micro-cube model, both domestically and internationally. The range of selected references is narrow, failing to fully reflect the major research achievements and theoretical developments in this field. I recommend that the authors expand the literature review to include more comprehensive studies on urban image promotion through short videos, particularly focusing on theoretical and methodological discussions in this area. Furthermore, the literature on the application of the micro-cube model in other fields should also be reviewed to provide a more complete academic context for the study.

**Response：**Thank you for your thoughtful and constructive feedback on our manuscript. We sincerely appreciate your detailed suggestions regarding the Literature Review section. Your comments have highlighted critical areas where our work can be improved, and we have carefully addressed them in our revisions.

Insufficient Explanation of the Key Elements: The six key elements proposed as the core components of urban image promotion through short videos are not sufficiently justified in the manuscript. While the selection of these elements seems logical, there is a lack of adequate theoretical rationale and empirical support to demonstrate why these six elements are the most critical. I recommend that the authors further explore and reference relevant literature or empirical studies to support the importance of these elements in short video promotion. Additionally, the authors should clarify how these elements specifically influence the effectiveness of urban image communication.

**Response：**Thank you for your constructive feedback and for highlighting the need for a more comprehensive theoretical and empirical rationale for the six key elements—unexpected events, emotional resonance, scene transition, elemental amplification, element interaction, and screen style—proposed in our study on urban image promotion through short videos. We have carefully addressed your comments and made substantial revisions to clarify and justify the selection and importance of these elements.

Insufficient Details in the Micro-Cube Model Construction: The paper proposes a visual and quantitative research method, but the construction of the micro-cube model lacks clear and detailed presentation of the experimental data and model-building process. This makes the model construction process opaque and prevents other researchers from replicating or adopting this method in future studies. To improve the reproducibility of the research, I suggest that the authors provide a more detailed explanation of each step in the micro-cube model construction, including the sources of experimental data, data processing methods, specific algorithms used, and the implementation process. This would not only enhance the credibility of the research but also offer clearer methodological guidance for future studies.

**Response：**Thank you for your thoughtful and constructive feedback regarding the lack of clarity and transparency in the construction of the Micro-Cube Model. We fully acknowledge the importance of presenting a clear, replicable, and well-documented methodology to ensure the robustness and reproducibility of our research. In response to your suggestion, we have significantly revised the relevant section to provide a comprehensive overview of the model-building process, covering data sources, processing methods, algorithmic logic, and implementation steps in a streamlined and coherent narrative.

The Micro-Cube Model was developed to provide a quantitative and visual analytical framework for understanding the relationship between short video content characteristics and their effectiveness in urban image promotion. The dataset, consisting of 20,668 video screenshots, was collected from the Douyin platform over a six-month period (July 2019 to December 2019). Video samples were carefully screened to ensure they included clear geographic tags and an explicit urban branding theme. Irrelevant, duplicate, or noise-laden samples were excluded to maintain data integrity. Data collection adhered to platform usage policies, and metadata such as upload date, hashtags, and account identity were cross-verified for accuracy.

The dataset was processed using Python, with Pandas and Matplotlib libraries applied for data cleaning, organization, and visualization. The video timeline was segmented into micro time units, each represented by a Micro-Cube, functioning as the fundamental analytical unit. These cubes encapsulate video content at a granular level, allowing us to isolate and measure six core visual elements: unexpected events, emotional resonance, scene transition, elemental amplification, element interaction, and screen style. Each element was assigned binary values (1 for presence, 0 for absence), and color-coding techniques were applied for visualization. For example, emotional resonance was represented using a nine-color emotional grid, while scene transitions were mapped using temporal and spatial segmentation rules. The degree of element interaction was calculated by identifying overlapping or dynamically connected visual elements within each Micro-Cube.

The algorithmic logic of the Micro-Cube Model includes systematic rules for identifying and quantifying these visual elements. For instance, unexpected events were detected by analyzing content anomalies across consecutive frames, while emotional resonance was assessed using color-matching ratios within emotional grids. Scene transitions were quantified based on abrupt shifts in temporal or spatial dimensions, and elemental amplification was measured by the percentage of screen area occupied by prominent visual details. The dynamic interactions between visual elements were decomposed into sub-categories using color-based sub-coding techniques, allowing us to visualize how elements interact over time. Additionally, screen style was analyzed through a classification of primary color tones (e.g., bright, dark, neutral), mapped across time intervals to understand their distribution and impact on audience perception.

The implementation process followed a sequential workflow: (1) Data segmentation into time-based units, (2) Assignment of binary codes to visual elements, (3) Visualization of Micro-Cube distributions using heatmaps and time-series charts, and (4) Quantitative analysis of relationships between these visual elements and audience engagement metrics, such as likes, comments, and shares. By overlaying these data layers within the Micro-Cube visualization framework, we were able to reveal correlations and patterns that drive the effectiveness of short video content in promoting urban imagery.

Finally, we ensured that the Micro-Cube Model remains replicable and adaptable for other researchers. The detailed methodological descriptions, coupled with transparent documentation of data sources, processing workflows, and algorithmic principles, enable other scholars to replicate, validate, and extend our approach across different datasets and research contexts. Furthermore, while our study focuses on urban image promotion, the Micro-Cube Model is inherently flexible and can be applied to areas such as brand communication analysis, cultural content dissemination, and digital media optimization.

We believe these revisions address your concerns by providing a coherent, transparent, and reproducible overview of the Micro-Cube Model. The enhanced clarity in the data sources, processing techniques, algorithmic implementation, and visualization workflows ensures a stronger methodological foundation for our research while offering clear guidance for future studies.

Thank you again for your valuable suggestions, which have greatly improved the methodological transparency of our paper. We look forward to your further feedback.
